# External validation of the COLOFIT colorectal cancer risk prediction model in the Oxford-FIT dataset: the importance of population characteristics and clinically relevant evaluation metrics

**DOI:** 10.1186/s12916-025-04339-w

**Published:** 2025-08-27

**Authors:** Andres Tamm, Brian Shine, Tim James, Jaimie Withers, Hizni Salih, Theresa Noble, Kinga A. Várnai, James E. East, Gary Abel, Willie Hamilton, Colin Rees, Eva J. A. Morris, Jim Davies, Brian D. Nicholson

**Affiliations:** 1 Nuffield Department of Primary Care Health Sciences, University of Oxford, Oxford, UK; 2 Big Data Institute, University of Oxford, Oxford, UK; 3 NIHR Oxford Biomedical Research Centre, Oxford, UK; 4 Department of Clinical Biochemistry, John Radcliffe Hospital, Oxford University Hospitals NHS Foundation Trust, Oxford, UK; 5 NIHR Health Informatics Collaborative, Oxford University Hospitals NHS Foundation Trust, Oxford, UK; 6 Translational Gastroenterology and Liver Unit, John Radcliffe Hospital, University of Oxford, Oxford, UK; 7 Nuffield Department of Population Health, University of Oxford, Oxford, UK; 8 Department of Computer Science, University of Oxford, Oxford, UK; 9 Medical School, University of Exeter, University of Exeter, Exeter, UK; 10 Population Health Sciences Institute, Newcastle University, Newcastle upon Tyne, UK; 11 Department of Gastroenterology, South Tyneside and Sunderland NHS Foundation Trust, South Tyneside, UK

**Keywords:** Faecal immunochemical test, Colorectal cancer, Risk prediction, External validation, Primary care

## Abstract

**Background:**

A faecal immunochemical test (FIT) result ≥ 10 µg/g is recommended in the UK to triage patients with symptoms of colorectal cancer (CRC) in primary care for urgent cancer investigation. The COLOFIT model combining FIT results with demographics and blood tests was developed to reduce the proportion of people referred without CRC. This study aims to externally validate the COLOFIT using data from Oxford University Hospitals (OUH).

**Methods:**

FITs requested by GPs between January 2017 and February 2024 were extracted from the OUH Clinical Data warehouse. Adults with COLOFIT predictors and 180-day follow-up for CRC were included. External validation of the COLOFIT equation was conducted overall and for six independent time periods. Risk score thresholds where the model captured the same number of cancers as FIT ≥ 10 µg/g were estimated to understand the number of urgent referrals avoided.

**Results:**

A total of 51,477 individuals (659 CRC) were included; 6194 (12%) had FIT ≥ 10 µg/g. FIT positivity and testing volume increased over time, associated with a gradual change from testing lower-risk patients to including those with higher-risk symptoms. COLOFIT was poorly calibrated overall (observed/expected [O/E] ratio 1.52 with calibration slope 1.05), but calibration improved over time (up to O/E ratio 1.09 with calibration slope 1.05). COLOFIT reduced referrals by 8% overall without missing colorectal cancers compared to FIT ≥ 10 µg/g, but this varied from 23% reduction to 2% increase depending on the period evaluated.

**Conclusions:**

The potential benefit of COLOFIT varied depending on FIT testing rates, the proportion of FIT ≥ 10 µg/g, and the symptoms in the tested population. Adopting COLOFIT into current clinical practice demands, therefore, FIT positivity of at least 17% and CRC rates within 1.3–1.6%. Further validation in local and different populations would also be of significant value and help to maximise COLOFIT’s ability to improve diagnostic pathways.

**Supplementary Information:**

The online version contains supplementary material available at 10.1186/s12916-025-04339-w.

## Research in context

### Evidence before this study

Clinical guidelines recommend that symptomatic FIT-positive patients are investigated for possible colorectal cancer, but as only one in eleven FIT-positive patients have cancer, there is potential to reduce referrals for urgent colonic investigation. The COLOFIT model combining FIT with age, sex, platelet count, and mean cell volume was developed to estimate the risk of colorectal cancer. Internal validation showed COLOFIT had the potential to reduce the number of referrals for colonic investigation while capturing a similar number of cancers as FIT alone. 

### Added value of this study

In this external validation, using data from Oxfordshire, the COLOFIT model reduced referrals by 8% overall while capturing a similar number of cancers as FIT alone. However, COLOFIT performance varied significantly over time ranging from 23% reduction to 2% increase in referrals. COLOFIT performance was influenced by the rate of FIT testing in the population, the proportion of positive FITs, and the symptoms that triggered the FIT.

### Implications

COLOFIT has the potential to reduce the number of whole colon examinations without missing colorectal cancer diagnoses compared to FIT alone. Targeted validation is recommended prior to implementation using data from the local population to determine the COLOFIT risk threshold that is most likely to lead to a reduction in referrals without missing colorectal cancer diagnoses. Ongoing monitoring and validation over time would ensure that COLOFIT performance was optimised in line with changes in FIT use in the local population. COLOFIT can be implemented before local validation if data are not available to evaluate it, given that FIT positivity and CRC rate are near derivation data values, but the model may not reduce referrals and a small number of cancers may be missed.

## Background

The Faecal Immunochemical Test (FIT) is recommended for use in primary care to triage patients with symptoms of possible colorectal cancer (CRC) for urgent colonic investigation, often colonoscopy. In the English National Health Service (NHS), National Institute for Health and Care Excellence (NICE) clinical guidelines initially recommended that FIT was used to triage patients with ‘low-risk’ symptoms of colorectal cancer, importantly excluding rectal bleeding [1]. Over time, interim guidance issued during the COVID-19 pandemic and later national recommendations moved to incorporate ‘high-risk’ patients with rectal bleeding or blood in stool [2–5]. FIT results ≥ 10 µg of haemoglobin per gram of faeces commonly trigger referral [2], with approximately 90% sensitivity for CRC [6]. However, about 10 in 11 patients with a positive FIT do not have cancer [6] (see Additional File 1, [5–16]). This is problematic as colonoscopy is an invasive investigation with a small risk of serious complications [17], endoscopy resources are often limited, and the NHS service is struggling to meet targets [18]. Multivariable prediction models have been developed that combine FIT with routine data to reduce referrals without missing CRC diagnoses [19].

The COLOFIT models were developed in Nottingham, England, as part of the National Institute for Health and Care Research (NIHR) Health Technology Assessment (HTA) COLOFIT programme [20]. They were developed using FITs requested by general practitioners (GP) to triage symptomatic patients before referral in primary care. The COLOFIT models were derived using the multivariate fractional polynomial algorithm [21]. The variables included were informed by a systematic review [19]. The final logistic and Cox COLOFIT models included FIT, age, sex, platelet count (PLT) and mean cell volume (MCV) as predictors [20].

This manuscript describes the external validation of the COLOFIT Cox model using the Oxford University Hospitals FIT dataset (OUH-FIT). Targeted external validation ensures that prediction models are validated in a population that matches their intended use [22]. The Oxford FIT dataset represents a similar population of GP-requested FITs. We investigated whether COLOFIT can lead to a reduction in the number of patients testing positive compared to the FIT ≥ 10 µg/g threshold, while capturing the same number of cancers. We also report discrimination, calibration, and net benefit statistics [23]. We provide software to compute these and other common metrics and provide guidance on factors to consider when implementing FIT-based risk stratification in a new population.

## Methods

### Ethics statement

Data use was approved by the OUH Information Governance Team, and the study was conducted as a service evaluation (under audit number 9076 in the OUH governance system).

### Oxford university hospitals FIT dataset (OUH-FIT)

Clinical data were extracted from the OUH clinical data warehouse for patients with a GP FIT request between January 2017 and February 2024, 180-day follow-up, and no records of CRC before FIT. The resulting OUH-FIT dataset covers demographics, FITs, blood tests, hospital attendances, histopathology reports, and other items.

#### Inclusion and exclusion criteria

Patients were excluded if (1) their FIT was not ordered by GP, (2) they were less than 18 years old, (3) they did not have 180 days of follow-up after their first FIT, (4) they had records of CRC before their first FIT, or (5) PLT and MCV results were not available. Patients who had CRC but did not have 180 days of follow-up from their FIT test were excluded. Inclusion of these patients would lead to incorrect estimation of cancer prevalence (and potentially incorrect estimates of model calibration), due to patients without cancer always being excluded with less than 180 days of follow-up. Patients with a known CRC diagnosis after 180 days from FIT were considered as non-cancers, so that cancer cases would be defined within the same follow-up window in older and newer time periods, to avoid any potential bias in cancer prevalence when comparing model performance across time. A sensitivity analysis was conducted by setting the follow-up window to 365 days.

#### Target population

FIT was adopted in 2016 by OUH [15, 24] and was initially used for triaging primary care patients with low-risk symptoms following the NICE DG30 guidance [6, 25]. In response to COVID, the Thames Valley Cancer Alliance recommended in July 2020 that FITs be offered to all primary care patients with possible CRC, including high-risk patients [3].

#### Sample collection and analysis

Before July 2021, faecal samples were predominantly collected into stool pots; buffer devices that prevent sample degradation were introduced since July 2021. From April 2022, a comment was added to the FIT result when a test was done in stool pot, allowing exploration of the impact of switching to buffered devices on positive FIT results. Faecal haemoglobin was measured with the HM-JACKarc analyser (Hitachi Chemical Diagnostics Systems Co., Ltd).

#### FIT values

FITs requested by GPs were identified using location codes and request numbers. The date of sample collection was used as the FIT date; if not known, the date of receipt was used. The first FIT value was selected for each patient. Values below the limit of detection (1.3 µg/g [24]) were replaced with zero, and values above the limit of linearity (400 µg/g [24]) with 400. Before applying the model, values below 4 µg/g were replaced with 4 to match the minimum value in model derivation data.

#### Predictor variables

Age at FIT was computed with monthly precision. PLT and MCV records within [− 365 days, + 14 days] from the earliest FIT were extracted, results after cancer date excluded, and the result closest to FIT was retained.

#### Additional variables

GP reported symptoms were extracted from notes associated with the FIT result by expanding on a previously used keyword search [26] and validated by inspecting the results (see Additional File 2) [26, 27]. Common treatments and procedures for CRC were extracted using OPCS-4 codes as previously [28]. Cancer T-stage was extracted from pathology reports using a regex-algorithm with at least 91% sensitivity and 94% positive predictive value (PPV) for current explicit TNM stages in OUH-FIT data [29].

#### Outcome variable (colorectal cancer)

Individuals were considered to have CRC if they had an International Classification of Diseases, version 10 (ICD-10) diagnosis code for CRC (C18-C20), or a pathology report that described current CRC. Pathology reports describing CRC were identified using a regex algorithm that had approximately 97% sensitivity and 92% PPV for identifying CRC reports among all main pathology reports of CRC patients in OUH-FIT data [29]; false positives were removed semi-manually (see Additional File 2) [26, 27]. The date of cancer was chosen to be the earliest among inpatient and outpatient diagnosis codes, and the date of receipt of the report. If a CRC treatment occurred within 180 days before the diagnosis code or report, the treatment date was used as the CRC date. 88% of the included patients had histopathology reports (see Additional File 3).

#### Missing values

Complete case analysis was used as the PLT and MCV test results were missing for only 5.9% of individuals (for 1.5% of cancer patients).

### External validation pipeline

#### Model validation over time

Given changes in case-mix and sample collection, the external validation was conducted overall plus after dividing the study period into six time intervals to evaluate model performance over time. COLOFIT was applied as an equation.

#### Core performance metric

A key aim was to assess whether COLOFIT could reduce the number of urgent referrals while capturing the same number of cancers as FIT at the NICE-recommended threshold of 10 µg/g. A COLOFIT model risk score threshold was chosen that captures the same number of cancers as FIT. This threshold was computed across the entire study period and for each independent time period of the OUH-FIT dataset, and was also estimated externally on COLOFIT derivation data. The number of patients who tested positive was compared to the number of patients testing positive for FIT at the 10 µg/g threshold to compute the potential reduction in referrals.

#### Other validation metrics

Discrimination, calibration, and net benefit statistics were computed (see Additional File 4: Table S4) [23, 30–34]. When computing decision curves, we included a spline model that predicts cancer from FIT values in OUH-FIT data as a comparator to cover all possible FIT thresholds.

#### Recalibration

COLOFIT was recalibrated using three methods: constant multiplication of FIT values, quantile transformation of FIT values, and logistic recalibration (see Additional File 5). FIT value transformations indicate if miscalibration was driven by differences in FIT values. Recalibrated models were used in decision curve analyses.

#### Prospective simulation

To simulate real-world use, the model was applied to each period using the COLOFIT threshold selected using data from the previous period. The potential reduction in referrals and the percentage of cancers missed relative to FIT were then computed.

#### Confidence intervals

Bootstrap percentile intervals were computed over 1000 samples, and test statistic distributions were visually examined. In each sample, receiver-operating characteristic (ROC) curves were interpolated linearly and precision-recall curves according to Davis and Goadrich [31]. Reductions in referrals were considered statistically significant if the 95% bootstrap confidence intervals excluded zero. Negative values in confidence intervals indicate reductions in, and positive values increases in referrals.

#### Sensitivity analysis

A sensitivity analysis was conducted with 365-day follow-up as this was the follow-up period used in model derivation.

#### Sample size

The study includes nearly all GP FITs in Oxfordshire. Confidence interval half-width for the core metric (reduction in referrals) is less than 3.2 in all time periods, which seems sufficiently accurate.

#### Software

Analysis was conducted in Python 3.9, primarily using *pandas* [35] and *scikit-learn* [36]. Code is available at https://github.com/tammandres/fitval.

### Patient and public involvement (PPI)

PPI were involved during model development [20]. The topic of FIT-based prediction models was discussed with the Oxford Primary Care Health Sciences cancer PPI group, where participants emphasised not missing other bowel diseases when models are optimised for CRC detection.

## Results

### Patient population

#### OUH-FIT dataset

**Fig. 1:**
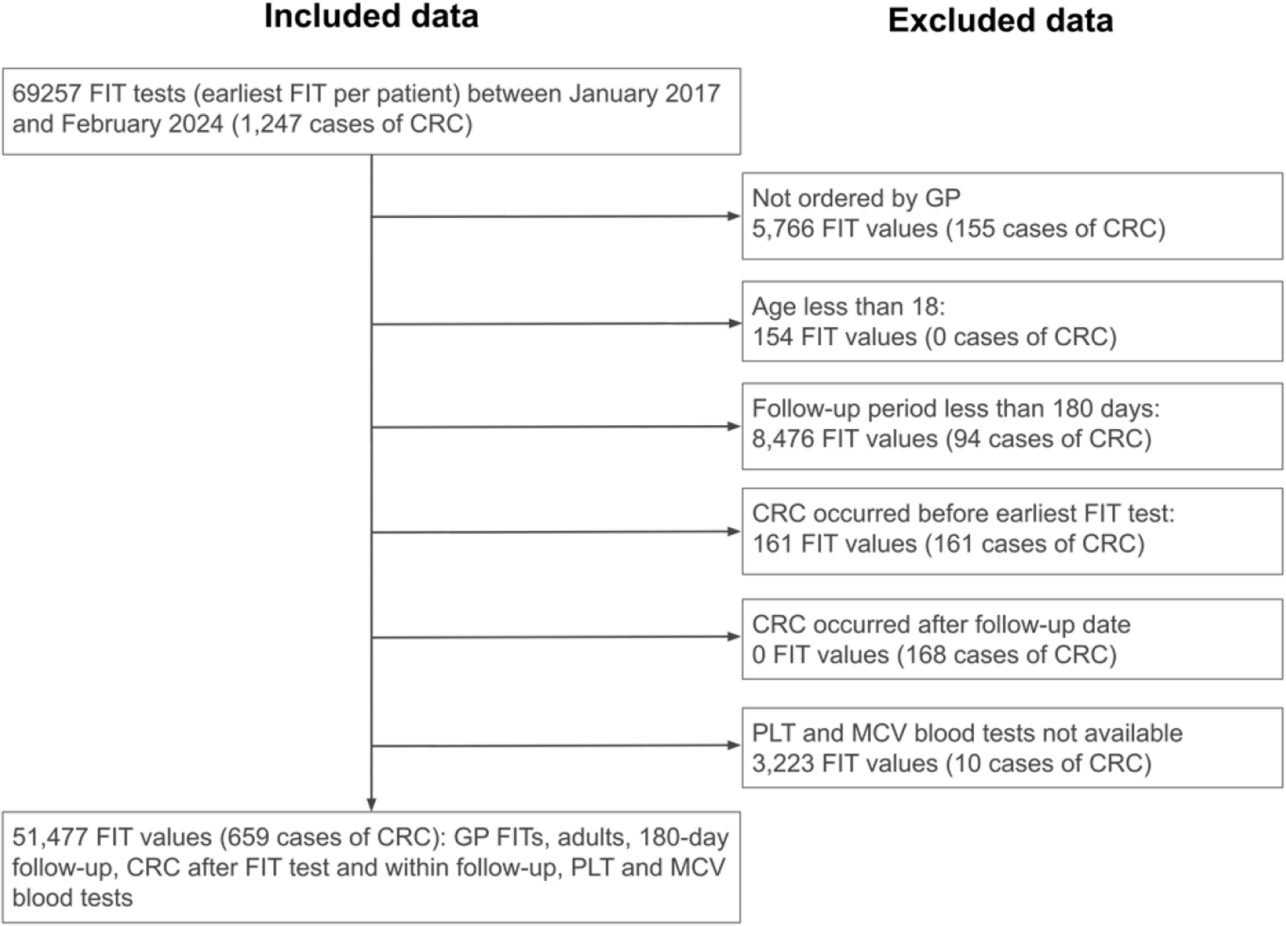
Flow diagram for building the OUH-FIT external validation cohort

FIT results were available for 69,257 patients (1,247 CRC). After applying the inclusion criteria (Fig. 1), 51,477 patients were retained (659 CRC). Exclusions were primarily due to non-GP FITs (5,766 tests) or lack of follow-up (8,476 tests). Prevalence of CRC was 1.28%. Descriptives are reported in Table 1. Compared to individuals without CRC, patients with CRC had higher median age (74.0 vs 62.7; Mann–Whitney *U* = 11,836,310, *p* < 0.001), were more likely to be male (56.3% vs 41.3%; *χ*^2^_(1)_ = 60.5, *p* < 0.001) and had a higher proportion of FITs at or above 10 µg/g (87.6% vs 11.1%; *χ*^2^_(1)_ = 3597.3, *p* < 0.001). CRC patients were also more likely to have abnormal blood test results (see Additional File 6: Table S6). There were 578 CRCs excluded: for 155, the FIT was performed in secondary care; for 161, the CRC was recorded before the FIT and likely to be detected via a non-GP route; 94 were in patients with less than 180 days of follow-up after FIT and 168 were recorded more than 180 days after FIT.

**Table 1:** Descriptive statistics for individuals included in the study

Characteristic	No colorectal cancer	Colorectal cancer	Statistical test
Number of patients	50,818 (98.7%)	659 (1.3%)	-
Age			
18–39.9	5899 (11.6%)	18 (2.7%)	*p* < 0.001 (*χ*^2^_(1)_ = 50.4)
40–49.9	6722 (13.2%)	46 (7.0%)	*p* < 0.001 (*χ*^2^_(1)_ = 22.2)
50–59.9	10,456 (20.6%)	87 (13.2%)	*p* < 0.001 (*χ*^2^_(1)_ = 21.7)
60–69.9	8834 (17.4%)	116 (17.6%)	*p* = 0.883 (*χ*^2^_(1)_ = 0.02)
70–79.9	10,671 (21.0%)	203 (30.8%)	*p* < 0.001 (*χ*^2^_(1)_ = 37.5)
≥ 80	8236 (16.2%)	189 (28.7%)	*p* < 0.001 (*χ*^2^_(1)_ = 73.9)
Median (25th, 75th)	62.7 (50.1, 75.9)	74.0 (61.4, 81.0)	*p* < 0.001 (*U* = 11,836,310)
Min and max	18.0, 102.7	26.9, 102.3	-
Gender			
F or non-binary (< 10)	29,846 (58.7%)	288 (43.7%)	-
M	20,972 (41.3%)	371 (56.3%)	*p* < 0.001 (*χ*^2^_(1)_ = 60.5)
Ethnicity			
Asian	1281 (2.5%)	Not available	Not available
Black	410 (0.8%)	Not available	Not available
Mixed	333 (0.7%)	Not available	Not available
Not known	1149 (2.3%)	Not available	Not available
Not stated	10,007 (19.7%)	149 (22.6%)	*p* = 0.061 (*χ*^2^_(1)_ = 3.5)
Other Ethnic Groups	519 (1.0%)	Not Available	Not available
White	37,119 (73.0%)	495 (75.1%)	*p* = 0.234 (*χ*^2^_(1)_ = 1.4)
Index of multiple deprivation (decile)			
Median (25th, 75th)	8.0 (7.0, 10.0)	9.0 (7.0, 10.0)	*p* = 0.008 (*U* = 13,504,500.5), complete cases
Min, max	1.0, 10.0	1.0, 10.0	-
Not known	4960 (9.8%)	32 (4.9%)	*p* < 0.001 (*χ*^2^_(1)_ = 17.9)
FIT (µg Hb/g)			
0–1.9	39,615 (78.0%)	46 (7.0%)	*p* < 0.001 (*χ*^2^_(1)_ = 1853.0)
2–9.9	5586 (11.0%)	36 (5.5%)	*p* < 0.001 (*χ*^2^_(1)_ = 448.2)
10–99.9	3845 (7.6%)	197 (29.9%)	*p* < 0.001 (*χ*^2^_(1)_ = 20.4)
≥ 100	1772 (3.5%)	380 (57.7%)	*p* < 0.001 (*χ*^2^_(1)_ = 4766.8)
Median (25th, 75th)	0.0 (0.0, 0.0)	165.0 (30.5, 400.0)	*p* < 0.001 (*U* = 2,557,706.5)
Min, max	0.0, 400.0	0.0, 400.0	-
Symptoms—GP reported			
Abdominal mass	61 (0.1%)	Not available	Not available
Abdominal pain	6937 (13.7%)	66 (10.0%)	*p* = 0.007 (*χ*^2^_(1)_ = 7.3)
Anaemia	6710 (13.2%)	128 (19.4%)	*p* < 0.001 (*χ*^2^_(1)_ = 21.8)
Bloating	1708 (3.4%)	Not available	Not available
Blood in stool	5506 (10.8%)	114 (17.3%)	*p* < 0.001 (*χ*^2^_(1)_ = 28.0)
Change in bowel habit	12,230 (24.1%)	173 (26.3%)	*p* = 0.192 (*χ*^2^_(1)_ = 1.7)
Constipation	1774 (3.5%)	10 (1.5%)	*p* = 0.006 (*χ*^2^_(1)_ = 7.6)
Diarrhoea	5818 (11.4%)	56 (8.5%)	*p* = 0.018 (*χ*^2^_(1)_ = 5.6)
Family history of CRC	486 (1.0%)	Not available	Not available
Fatigue	823 (1.6%)	Not available	Not available
Inflammation	631 (1.2%)	Not available	Not available
Iron deficiency anaemia	2937 (5.8%)	61 (9.3%)	*p* < 0.001 (*χ*^2^_(1)_ = 14.3)
Low iron	1443 (2.8%)	Not available	Not available
Melaena	526 (1.0%)	Not available	Not available
Not known	7380 (14.5%)	87 (13.2%)	*p* = 0.339 (*χ*^2^_(1)_ = 0.9)
Rectal bleeding	3495 (6.9%)	70 (10.6%)	*p* < 0.001 (*χ*^2^_(1)_ = 14.2)
Rectal mass	31 (0.1%)	Not available	Not available
Rectal pain	317 (0.6%)	Not available	Not available
Thrombocytosis	559 (1.1%)	Not available	Not available
Weight loss	3805 (7.5%)	50 (7.6%)	*p* = 0.923 (*χ*^2^_(1)_ = 0.009)
CRC-relevant treatments			
No treatments recorded	48,595 (95.6%)	136 (20.6%)	*p* < 0.001 (*χ*^2^_(1)_ = 7244.3)
Chemotherapy	1314 (2.6%)	272 (41.3%)	*p* < 0.001 (*χ*^2^_(1)_ = 3261.1)
Local excision	56 (0.1%)	26 (3.9%)	*p* < 0.001 (*χ*^2^_(1)_ = 601.7)
Radical resection	227 (0.4%)	379 (57.5%)	*p* < 0.001 (*χ*^2^_(1)_ = 18,209.9)
Radiotherapy	997 (2.0%)	99 (15.0%)	p < 0.001 (*χ*^2^_(1)_ = 532.6)
T stage			
1	-	67 (10.2%)	-
2	-	56 (8.5%)	-
3	-	169 (25.6%)	-
4	-	75 (11.4%)	-
Not known	-	292 (44.3%)	-
N stage			
0	-	199 (30.2%)	-
1	-	70 (10.6%)	-
2	-	45 (6.8%)	-
Not known	-	345 (52.4%)	-

Rows with ‘Not available’ had a count less than 10. If one count in a category was less than 10, or all counts less than 10 did not add up to 10, the next highest count was also marked ‘Not available’. This is due to data reporting requirements. Abbreviations: *CRC* colorectal cancer, *GP* general practitioner, *FIT* faecal immunochemical test

#### Comparison of model derivation and validation datasets

There were 34,435 patients (533 cancers) in the Nottingham COLOFIT derivation dataset. The Nottingham derivation and OUH-FIT validation datasets were comparable in terms of the proportion of CRC (1.55% Nottingham, 1.28% OUH-FIT), the quartiles of continuous predictors (age, MCV, PLT), gender (43.7% males in Nottingham, 41.4% in OUH-FIT), and white ethnicities (70.3% Nottingham, 73.1% OUH-FIT; see Table 2). However, 22.4% of patients had a positive FIT at the 10 µg/g threshold in the Nottingham derivation data, compared to 12.0% in OUH-FIT data. The approximate number of FITs per year was also higher in Nottingham (8,609) than in Oxford (7,267).

**Table 2:** Comparison of the Nottingham model derivation and Oxford validation datasets

**Characteristic**	**Nottingham (model derivation data) **[20]	**Oxford** **(OUH-FIT)**
Number of patients	34,435	51,477
Study period	Nov 2017–Nov 2021	Jan 2017–Feb 2024
Colorectal cancer		
Colorectal cancer cases (%)	533 (1.55%)	659 (1.28%)
Timing between FIT and cancer	Up to 12 months	Up to 6 months
Identification of cancer cases	Nottingham University Hospitals (NUH) cancer database	Histopathology reports (88.3%), inpatient diagnosis codes (11.4%), outpatient diagnosis codes (0.3%)
FIT testing		
Setting of FIT testing	Primary care	Primary care
Symptoms tested	‘High-risk’ and ‘low-risk’ symptoms	‘Low-risk’ to include ‘high-risk’ symptoms over time
FIT sample collection device	Primarily buffer device	Stool pot until June 2021, then transition to buffer device
FIT analytical method	OC-Sensor PLEDIA	HM-JACKarc
FIT reported range (µg Hb/g faeces)	< 4–69,800	< 1.3–400
FIT values, median (25th, 75th)	4 (4, 8)	0 (0, 0)
FIT ≥ 10 µg/g (%)	22.4	12.0
Approximate number of FITs per year	8609	7267
Other predictor variables in the model		
Age range (years)	> 18	18–103
Age, median (25th, 75th percentile)	66 (54, 77)	62 (51, 75)
Gender male (%)	43.7%	41.4%
Mean cell volume, median (25th, 75th)	92 (88, 96)	92 (88, 95)
Platelets, median (25th, 75th)	268 (219, 324)	267 (224, 317)
Ethnicity		
White	70.8%	73.1%
Asian	4.3%	2.5%
Black	2.5%	0.8%
Other or mixed	1.9%	1%
Not recorded	20.6%	21.9%

*FIT* faecal immunochemical test

#### Changes in the patient population

**Fig. 2:**
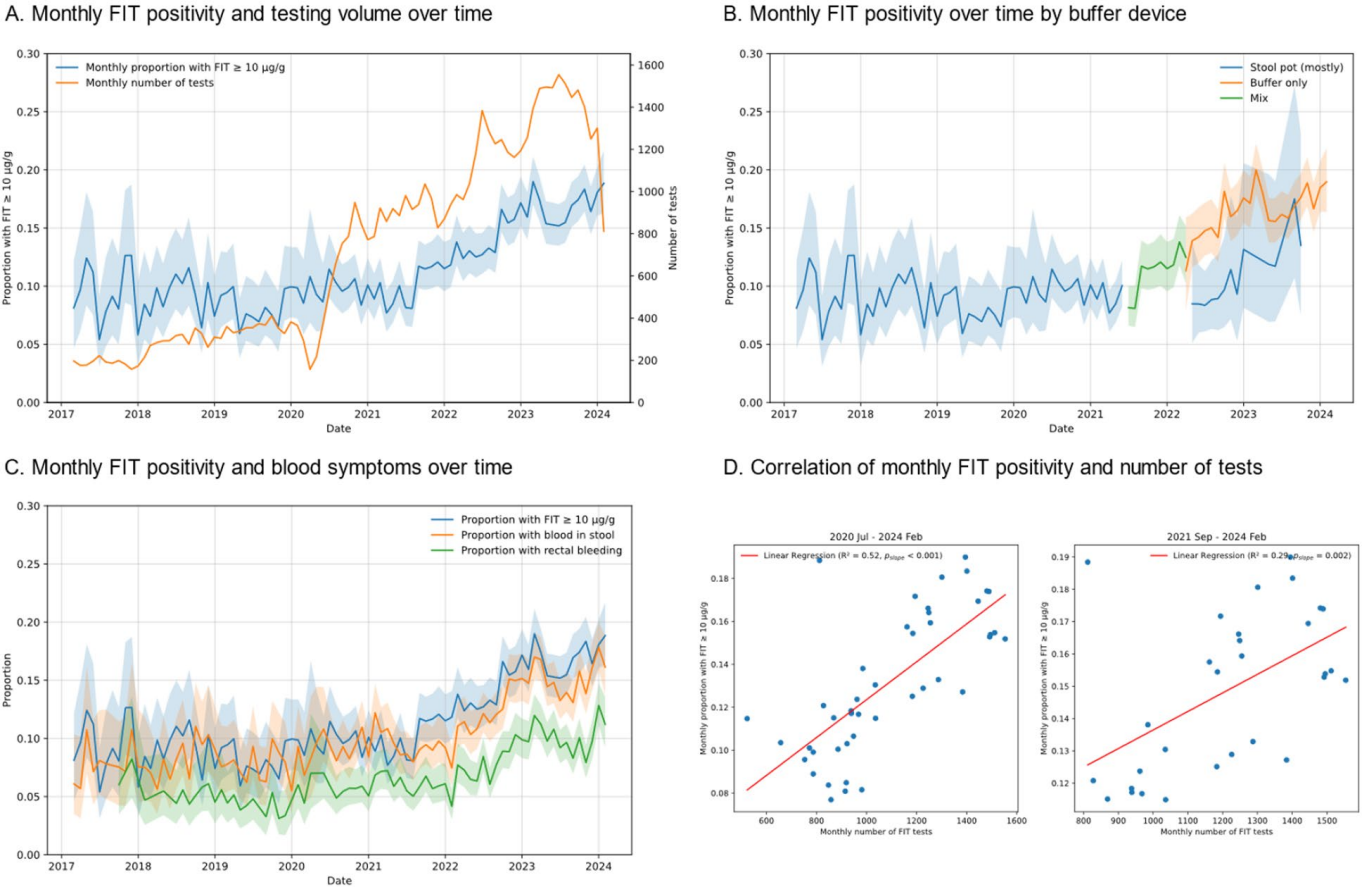
Changes in the FIT testing population over time. **A** Proportion of patients testing positive for FIT at the 10 µg/g threshold each month (left axis, blue line), and number of FITs recorded each month (right axis, orange line). **B** Proportion of patients testing positive for FIT at the 10 µg/g threshold each month, grouped by FIT sample collection device. Before July 2021, FIT samples were usually collected into stool pots (blue line), and buffer devices were introduced afterwards. Since April 2022, it is known how many tests were returned in buffer devices as comments about the stool pot were available alongside FIT results (orange line). Data about stool pots was not available between July 2021 and April 2022 (green line). **C** Proportion of patients testing positive for FIT at the 10 µg/g threshold each month, and proportion of patients presenting with blood in stool (which includes rectal bleeding) or with rectal bleeding only each month. Please note that increases in blood symptoms do not fully explain increases in FIT positivity, because not all patients with these symptoms will have a positive FIT: chances of a positive FIT were approximately 21% for patients with blood symptoms and 10% for patients without. **D** Monthly number of FIT tests plotted against monthly proportion of patients testing positive for the FIT test at the 10 µg/g threshold. Graphs are shown for two time periods: 2020 July to 2024 February (middle)—the period when monthly number of FIT tests was increasing; 2021 September to 2024 February (right)—the period when both monthly FIT positivity and monthly number of tests were increasing (no obvious correlations were seen for the period before). *R*^2^ is the coefficient of determination; *p*_slope_ is the *p*-value of the regression slope. In **A**–**C**, shaded areas show 95% Wilson confidence intervals and proportions were computed only for months with at least 10 positive events

A change in the FIT testing population over time was observed consistent with guidance provided by the Thames Valley Cancer Alliance in July 2020 that FITs be used to also triage patients with higher-risk symptoms. Firstly, the monthly volume of FITs has increased from fewer than 411 tests before July 2020 to usually more than 1,400 tests since April 2023 (Fig. 2A), and this increase was correlated with a greater monthly FIT positivity (Fig. 2D). Secondly, there has been an increasing trend in the proportion of patients testing positive for FIT at the 10 µg/g threshold: FIT positivity has increased from 11.7% in September 2021 to 18.8% in February 2024 (Fig. 2A). Thirdly, the proportion of patients testing positive for FIT has similarly increased among patients who did their FITs with the buffer device only, showing that increased FIT positivity was not related to changes in the faecal sampling method (Fig. 2B). Less than 4% increase in FIT positivity can be attributed to the buffer device (see Additional File 7). Fourthly, the proportion of patients tested for rectal bleeding (a ‘high-risk’ symptom [1]) and blood in stool has increased (Fig. 2C).

### The OUH-FIT was divided into six time periods

The OUH-FIT data was divided into six periods to better take population changes into account (Table 3). We separated out the COVID period due to its unique circumstances and did not subdivide the pre-COVID period due to the smaller testing volume. Periods after COVID represent 6-month intervals, except the first post-COVID period which covers 8 months. All periods have a broadly similar number of cancers (88 to 128). Data from July and August 2023 were excluded so the last three periods have equal duration and seasonality. Descriptives are given in Additional File 8: Tables S8A and S8B.

**Table 3:** Time-periods used in the analysis

Time period	Number of FIT tests	Number of cancers	Prevalence of cancer (%)	Percent FIT ≥ 10 µg/g
All data (2017 Jan–2023 Aug)	51,477	659	1.28	12.0
Pre-COVID (2017 Jan–2020 Feb)	10,379	124	1.19	8.8
COVID (2020 Mar–2021 Apr)	8890	128	1.44	9.6
Post-COVID (2021 May–2021 Dec)	7472	99	1.32	10.2
2022 H1 (2022 Jan–Jun)	5972	88	1.47	12.5
2022 H2 (2022 Jul–Dec)	7490	104	1.39	14.4
2023 H1 (2023 Jan–Jun)	8320	91	1.09	16.7

Prevalence refers to colorectal cancer. Abbreviations: *FIT* – faecal immunochemical test

### Reduction in referrals

#### Reduction in referrals using the Nottingham-derived threshold

**Fig. 3:**
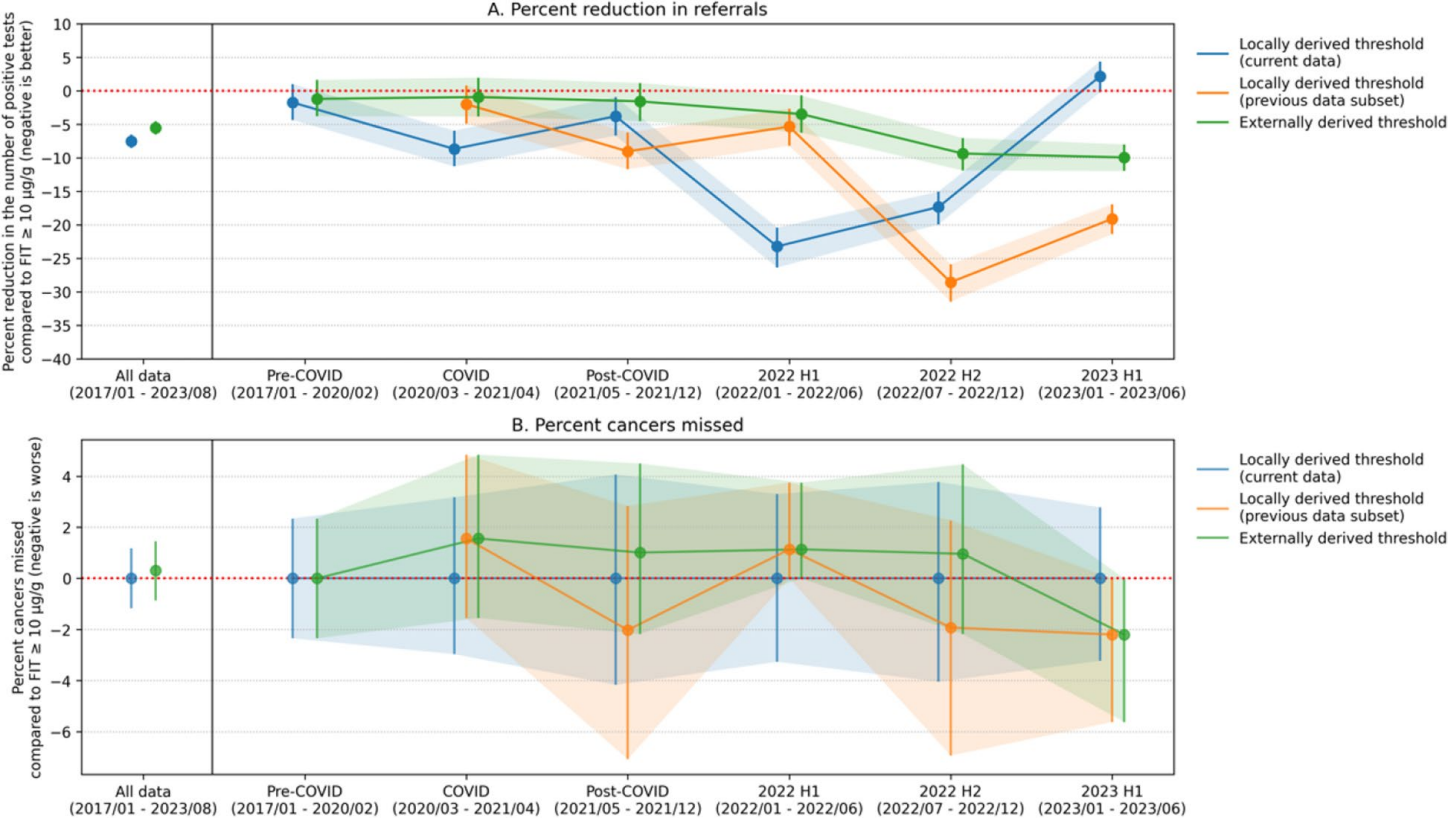
Percent reduction in the number of positive tests (referrals) and percent cancers missed compared to FIT ≥ 10 µg/g over time in OUH-FIT data. The model was evaluated at a threshold that captured the same number of cancers as FIT ≥ 10 µg/g in the current Oxford data subset (blue), in the previous Oxford data subset (orange), or in external model derivation data (green). When percent reduction is negative, it means that the number of patients who would have tested positive according to the model was smaller than the number of patients who tested positive for FIT, and so there was potentially a reduction in referrals. When the model was evaluated using thresholds computed on the previous Oxford data subset or on external data, the model did not always capture the same number of cancers as FIT: the percentage of cancers missed (or extra cancers detected) compared to FIT ≥ 10 µg/g is shown in the lower panel. For example, ‘ − 2%’ means that 2% cancers would have been missed relative to the FIT test. Shaded areas show 95% percentile bootstrap confidence intervals. The points are slightly jittered on the x-axis to avoid overlap

In the COLOFIT derivation data, a 0.64% risk score threshold captured a similar number of cancers as FIT ≥ 10 µg/g. Using this threshold in the OUH-FIT data, the COLOFIT would have led to a 5.51% (95% CI [− 6.54, − 4.51]) reduction in referrals with 0.3% extra cancers detected overall; 1.2% (95% CI [− 3.78, 1.61]) reduction in referrals with no cancers missed pre-COVID; a 0.94% (95% CI [− 3.85, 1.97]) reduction with 1.56% extra cancers detected during COVID; a 1.57% (95% CI [− 4.52, 1.16]) reduction with 1.01% extra cancers detected post-COVID; a 3.47% (95% CI [− 6.26, − 0.71]) reduction with 1.14% extra cancers detected in 2022 H1; a 9.37% (95% CI [− 11.88, − 7.05]) reduction with 0.96% extra cancers detected in 2022 H2; and a 9.95% (95% CI [− 11.98, − 8.0]) reduction with 2.2% cancers missed in 2023 H1 (‘external’ in Table 4, Fig. 3). Reductions were statistically significant (95% confidence intervals excluded zero) on all data and in 2022–2023.

**Table 4:** Percent reduction in the number of patients that test positive when the COLOFIT model would be used instead of the FIT test, and when the model is evaluated at a threshold that captures the same number of cancers as FIT

Model threshold method*	Prevalence of colorectal cancer (%)	Percent reduction in number of positive tests**	Positive tests per 1000 tests (model)	Positive tests per 1000 tests (FIT)	Delta sensitivity (sensitivity model minus sensitivity FIT)***	Sensitivity model (%)	Sensitivity FIT (%)	Total num cancers	Num cancers missed or gained relative to FIT ****	Model threshold (%)
All data (2017/01–2023/08)										
Local current	1.28 (1.18, 1.38)	− 7.51 (− 8.53, − 6.53)	111.29 (108.59, 113.99)	120.33 (117.72, 123.41)	0.0 (− 1.17, 1.18)	87.56 (85.27, 90.02)	87.56 (85.15, 89.99)	659	0 (− 8, 8)	0.68
External	1.28 (1.18, 1.38)	− 5.51 (− 6.54, − 4.51)	113.7 (110.92, 116.44)	120.33 (117.72, 123.41)	0.3 (− 0.86, 1.46)	87.86 (85.57, 90.24)	87.56 (85.15, 89.99)	659	2 (− 6, 10)	0.64
Pre-COVID (2017/01–2020/02)										
Local current	1.19 (0.99, 1.41)	− 1.75 (− 4.35, 0.99)	86.52 (81.03, 91.92)	88.06 (82.47, 93.46)	0.0 (− 2.34, 2.34)	86.29 (79.82, 92.56)	86.29 (80.43, 92.56)	124	0 (− 3, 3)	0.65
External	1.19 (0.99, 1.41)	− 1.2 (− 3.78, 1.61)	87.0 (81.41, 92.4)	88.06 (82.47, 93.46)	0.0 (− 2.34, 2.34)	86.29 (79.82, 92.56)	86.29 (80.43, 92.56)	124	0 (− 3, 3)	0.64
COVID (2020/03–2021/04)										
Local current	1.44 (1.19, 1.68)	− 8.68 (− 11.29, − 5.95)	87.63 (82.22, 93.93)	95.95 (90.1, 101.58)	0.0 (− 2.96, 3.17)	85.94 (79.72, 91.94)	85.94 (79.64, 91.47)	128	0 (− 4, 4)	0.78
Local previous	1.44 (1.19, 1.68)	− 1.99 (− 4.9, 0.81)	94.04 (88.41, 100.34)	95.95 (90.1, 101.58)	1.56 (− 1.55, 4.84)	87.5 (81.1, 93.16)	85.94 (79.64, 91.47)	128	2 (− 2, 6)	0.65
External	1.44 (1.19, 1.68)	− 0.94 (− 3.85, 1.97)	95.05 (89.31, 101.35)	95.95 (90.1, 101.58)	1.56 (− 1.55, 4.84)	87.5 (81.1, 93.16)	85.94 (79.64, 91.47)	128	2 (− 2, 6)	0.64
Post-COVID (2021/05–2021/12)										
Local current	1.32 (1.07, 1.62)	− 3.8 (− 6.69, − 0.93)	98.23 (91.41, 105.46)	102.11 (95.15, 109.34)	0.0 (− 4.17, 4.07)	82.83 (75.45, 89.8)	82.83 (74.77, 90.22)	99	0 (− 4, 4)	0.68
Local previous	1.32 (1.07, 1.62)	− 9.04 (− 11.7, − 6.22)	92.88 (86.45, 99.58)	102.11 (95.15, 109.34)	− 2.02 (− 7.07, 2.83)	80.81 (72.9, 88.42)	82.83 (74.77, 90.22)	99	− 2 (− 7, 3)	0.78
External	1.32 (1.07, 1.62)	− 1.57 (− 4.52, 1.16)	100.51 (93.81, 107.47)	102.11 (95.15, 109.34)	1.01 (− 2.18, 4.49)	83.84 (76.67, 90.66)	82.83 (74.77, 90.22)	99	1 (− 2, 4)	0.64
2022 H1 (2022/01–2022/06)										
Local current	1.47 (1.21, 1.79)	− 23.23 (− 26.38, − 20.43)	96.28 (89.25, 103.49)	125.42 (117.88, 133.79)	0.0 (− 3.26, 3.3)	89.77 (82.42, 95.51)	89.77 (82.35, 95.41)	88	0 (− 3, 3)	1.28
Local previous	1.47 (1.21, 1.79)	− 5.34 (− 8.21, − 2.66)	118.72 (111.19, 126.76)	125.42 (117.88, 133.79)	1.14 (0.0, 3.75)	90.91 (83.87, 96.2)	89.77 (82.35, 95.41)	88	1 (0, 3)	0.68
External	1.47 (1.21, 1.79)	− 3.47 (− 6.26, − 0.71)	121.06 (113.19, 129.44)	125.42 (117.88, 133.79)	1.14 (0.0, 3.75)	90.91 (83.87, 96.2)	89.77 (82.35, 95.41)	88	1 (0, 3)	0.64
2022 H2 (2022/07–2022/12)										
Local current	1.39 (1.13, 1.66)	− 17.35 (− 19.95, − 15.06)	118.96 (111.62, 126.44)	143.93 (136.58, 152.07)	0.0 (− 4.04, 3.77)	88.46 (82.1, 94.06)	88.46 (81.98, 94.29)	104	0 (− 4, 4)	0.84
Local previous	1.39 (1.13, 1.66)	− 28.57 (− 31.46, − 25.89)	102.8 (96.13, 109.75)	143.93 (136.58, 152.07)	− 1.92 (− 6.93, 2.27)	86.54 (80.0, 92.59)	88.46 (81.98, 94.29)	104	− 2 (− 7, 2)	1.28
External	1.39 (1.13, 1.66)	− 9.37 (− 11.88, − 7.05)	130.44 (123.1, 137.79)	143.93 (136.58, 152.07)	0.96 (− 2.17, 4.46)	89.42 (83.17, 95.0)	88.46 (81.98, 94.29)	104	1 (− 2, 5)	0.64
2023 H1 (2023/01–2023/06)										
Local current	1.09 (0.87, 1.3)	2.16 (− 0.21, 4.38)	170.31 (162.5, 178.85)	166.71 (159.13, 175.12)	0.0 (− 3.23, 2.78)	92.31 (87.23, 97.26)	92.31 (86.73, 97.56)	91	0 (− 3, 3)	0.47
Local previous	1.09 (0.87, 1.3)	− 19.11 (− 21.37, − 16.97)	134.86 (127.16, 142.55)	166.71 (159.13, 175.12)	− 2.2 (− 5.62, 0.0)	90.11 (84.09, 95.61)	92.31 (86.73, 97.56)	91	− 2 (− 5, 0)	0.84
External	1.09 (0.87, 1.3)	− 9.95 (− 11.98, − 8.0)	150.12 (142.43, 157.82)	166.71 (159.13, 175.12)	− 2.2 (− 5.62, 0.0)	90.11 (84.09, 95.61)	92.31 (86.73, 97.56)	91	− 2 (− 5, 0)	0.64

*Model threshold method: A threshold was computed for the model such that it detects the same number of cancers as FIT test at threshold 10 µg/g while having as low false positive rate as possible. The threshold was computed on three different data subsets: (a) on external Nottingham data (‘external’); (b) on current subset of Oxford data (‘local current’); and (c) on previous subset of Oxford data (‘local previous’). **Percent reduction in number of positive tests: when percent reduction in the number of positive tests is less than zero, it indicates that fewer patients tested positive when the model was used and so the model would have potentially led to fewer referrals. ***Delta sensitivity represents the difference in sensitivities between the model and the FIT test, and thus shows the percentage of cancers missed (when it is negative) or extra cancers detected (when it is positive) compared to the FIT test. ****Num cancers missed or gained relative to FIT shows the absolute number of cancers that would have been missed relative to FIT ≥ 10 µg/g (negative numbers) or absolute number of extra cancers detected relative to FIT ≥ 10 µg/g (positive numbers). All confidence intervals are bootstrap percentile confidence intervals. Abbreviations: *FIT*, faecal immunochemical test

#### Reduction in referrals using the OUH-FIT locally derived threshold

When a threshold was chosen based on OUH-FIT data such that COLOFIT captured as many cancers as FIT ≥ 10 µg/g, COLOFIT yielded a 7.51% (95% CI [− 8.53, − 6.53]) reduction in referrals (‘all data: local current’ in Table 4). When optimal thresholds were computed separately for each time period so that no cancers were missed, the reduction in referrals was 1.75% (95% CI [− 4.35, 0.99]) pre − COVID; 8.68% (95% CI [− 11.29, − 5.95]) during COVID; 3.8% (95% CI [− 6.69, − 0.93]) post − COVID; 23.23% (95% CI [− 26.38, − 20.43]) in 2022 H1; 17.35% (95% CI [− 19.95, − 15.06]) in 2022 H2; and a 2.16% (95% CI [− 0.21, 4.38]) increase in 2023 H1 (‘local current’ in Table 4, Fig. 3). Reductions were statistically significant during COVID and in 2022 (95% confidence intervals excluded zero). The optimal referral threshold was 0.68% for all data and between 0.47% and 1.28% for individual time periods.

#### Simulation of prospective use of the locally derived threshold

To simulate prospective use, the model was applied to data in each period using the optimal threshold computed with data from the previous analytical period. In this simulation, there was a 1.99% (95% CI [− 4.9, 0.81]) reduction in referrals during COVID with 1.56% extra cancers detected; post-COVID there was a 9.04% (95% CI [− 11.7, − 6.22]) reduction with 2.02% cancers missed; in 2022 H1 there was a 5.34% (95% CI [− 8.21, − 2.66]) reduction with 1.14% extra cancers detected; in 2022 H2 there was a 28.57% (95% CI [− 31.46, − 25.89]) reduction with 1.92% cancers missed; and in 2023 H1 there was a 19.11% reduction (95% CI [− 21.37, − 16.97]) with 2.2% cancers missed (‘local previous’ in Table 4, Fig. 3). Reductions were statistically significant (95% confidence intervals excluded zero) post-COVID and in subsequent periods.

#### Sensitivity analysis

Increasing the follow-up period to 365 days had no effect or a small effect on the reduction in referrals in time periods where both follow-up lengths were available (see Additional File 9).

### Calibration

**Fig. 4:**
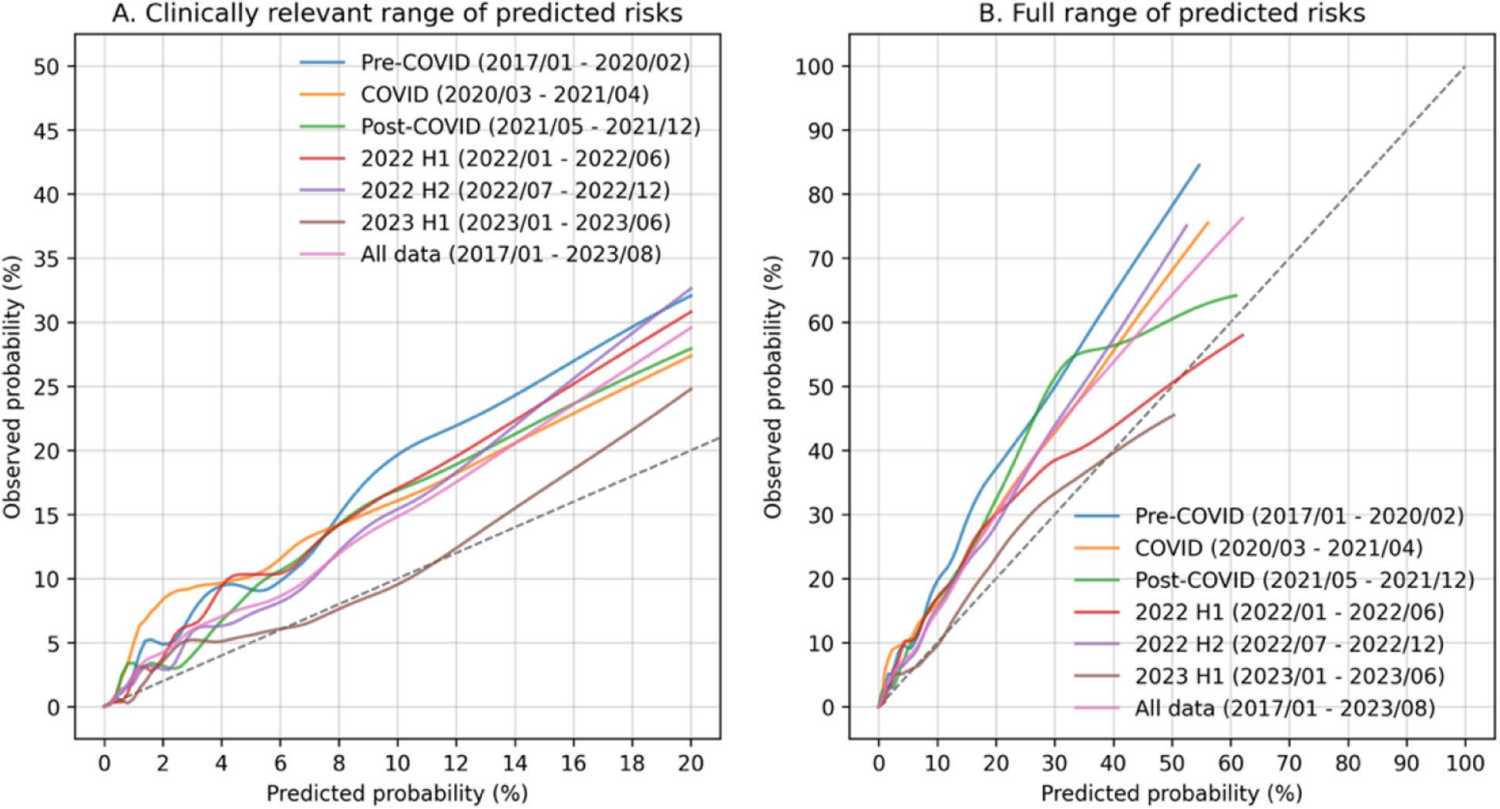
LOWESS-calibration curves for the COLOFIT Cox model over six time periods in the OUH-FIT dataset. **A** Curves for predicted probabilities less than 20%. **B** Curves over the full range of predicted probabilities

COLOFIT was poorly calibrated: predicted probabilities were on average 1.52 times lower than expected on all data, and 1.48 to 1.82 times lower in the five time periods before 2023 (observed/expected [O/E] ratio in Table 5). Calibration improved in 2023 H1, where the O/E ratio was 1.09. An examination of predicted against true probabilities revealed that COLOFIT under-predicted over the full range of predicted risks before 2023 (Fig. 4). The model could be approximately recalibrated using quantile transformation of FIT values, indicating that differences in FIT values between Nottingham and Oxford determined miscalibration (Table 5, Additional File 10: Figure S10). Calibration improved over time as the proportion of patients with FIT ≥ 10 µg/g approached the proportion in model derivation data (see Table 3 for the proportions). Miscalibration was also partly caused by differences in the FIT analytic device (see Additional File 11).

**Table 5:** Overall calibration metrics for the original and recalibrated COLOFIT models

Model	Event rate (%)	Mean risk (%)	O/E ratio	Logistic intercept	Logistic slope
All data (2017/01–2023/08)					
FIT-spline	1.28 (1.18, 1.37)	1.28 (1.25, 1.32)	1.0 (0.93, 1.07)	− 0.0 (− 0.15, 0.16)	1.0 (0.95, 1.05)
COLOFIT-Cox	1.28 (1.18, 1.37)	0.84 (0.81, 0.87)	1.52 (1.42, 1.63)	0.64 (0.48, 0.8)	1.05 (1.0, 1.09)
COLOFIT-Cox-quant	1.28 (1.18, 1.37)	1.27 (1.25, 1.29)	1.01 (0.94, 1.08)	0.21 (0.05, 0.37)	1.08 (1.03, 1.13)
Pre-COVID (2017/01–2020/02)					
FIT-spline	1.19 (0.99, 1.4)	1.2 (1.11, 1.28)	1.0 (0.84, 1.16)	− 0.0 (− 0.32, 0.32)	1.0 (0.9, 1.13)
COLOFIT-Cox	1.19 (0.99, 1.4)	0.66 (0.61, 0.71)	1.81 (1.52, 2.08)	1.05 (0.68, 1.43)	1.11 (1.02, 1.22)
COLOFIT-Cox-quant	1.19 (0.99, 1.4)	1.35 (1.3, 1.4)	0.88 (0.74, 1.04)	0.06 (− 0.29, 0.45)	1.09 (0.97, 1.23)
COVID (2020/03–2021/04)					
FIT-spline	1.44 (1.2, 1.69)	1.44 (1.35, 1.52)	1.0 (0.84, 1.16)	− 0.0 (− 0.33, 0.35)	1.0 (0.9, 1.13)
COLOFIT-Cox	1.44 (1.2, 1.69)	0.79 (0.72, 0.86)	1.82 (1.52, 2.09)	0.79 (0.42, 1.13)	1.02 (0.93, 1.12)
COLOFIT-Cox-quant	1.44 (1.2, 1.69)	1.37 (1.31, 1.43)	1.05 (0.88, 1.22)	0.2 (− 0.15, 0.53)	1.06 (0.96, 1.18)
Post-COVID (2021/05–2021/12)					
FIT-spline	1.32 (1.08, 1.58)	1.33 (1.24, 1.42)	1.0 (0.82, 1.18)	− 0.01 (− 0.4, 0.37)	1.0 (0.88, 1.15)
COLOFIT-Cox	1.32 (1.08, 1.58)	0.79 (0.72, 0.85)	1.68 (1.39, 1.98)	0.65 (0.22, 1.09)	1.01 (0.91, 1.13)
COLOFIT-Cox-quant	1.32 (1.08, 1.58)	1.37 (1.32, 1.42)	0.97 (0.79, 1.15)	0.12 (− 0.31, 0.54)	1.06 (0.93, 1.23)
2022 H1 (2022/01–2022/06)					
FIT-spline	1.47 (1.19, 1.79)	1.47 (1.37, 1.59)	1.0 (0.81, 1.2)	0.0 (− 0.42, 0.41)	1.0 (0.87, 1.17)
COLOFIT-Cox	1.47 (1.19, 1.79)	0.96 (0.87, 1.05)	1.54 (1.25, 1.85)	0.67 (0.21, 1.1)	1.05 (0.94, 1.19)
COLOFIT-Cox-quant	1.47 (1.19, 1.79)	1.35 (1.28, 1.42)	1.09 (0.88, 1.32)	0.27 (− 0.17, 0.73)	1.06 (0.93, 1.22)
2022 H2 (2022/07–2022/12)					
FIT-spline	1.39 (1.13, 1.67)	1.39 (1.3, 1.48)	1.0 (0.83, 1.18)	0.01 (− 0.36, 0.41)	1.0 (0.89, 1.16)
COLOFIT-Cox	1.39 (1.13, 1.67)	0.94 (0.86, 1.02)	1.48 (1.24, 1.75)	0.66 (0.29, 1.08)	1.07 (0.96, 1.2)
COLOFIT-Cox-quant	1.39 (1.13, 1.67)	1.24 (1.18, 1.29)	1.12 (0.93, 1.34)	0.41 (0.02, 0.83)	1.1 (0.98, 1.25)
2023 H1 (2023/01–2023/06)					
FIT-spline	1.09 (0.87, 1.31)	1.09 (1.04, 1.16)	1.0 (0.8, 1.2)	− 0.0 (− 0.46, 0.51)	1.0 (0.86, 1.19)
COLOFIT-Cox	1.09 (0.87, 1.31)	1.0 (0.93, 1.07)	1.09 (0.88, 1.31)	0.24 (− 0.16, 0.66)	1.05 (0.94, 1.19)
COLOFIT-Cox-quant	1.09 (0.87, 1.31)	1.12 (1.08, 1.17)	0.98 (0.77, 1.17)	0.17 (− 0.25, 0.57)	1.07 (0.96, 1.2)

O/E ratio – observed/expected ratio. Logistic intercept and logistic slope are the intercept and slope of a logistic regression model that predicts the colorectal cancer rate from the logits of predicted probabilities of cancer. COLOFIT-Cox-quant is a recalibrated version of the COLOFIT-Cox model, where the FIT test values were transformed to be more similar to Nottingham FIT values using quantile transformation, before entering them to the model. FIT-spline is a model developed on Oxford data that predicts the risk of colorectal cancer from FIT test values, and is only used as a comparator for the Nottingham model in decision curve analysis. Abbreviations: *FIT*, faecal immunochemical test

### Discrimination

**Fig. 5:**
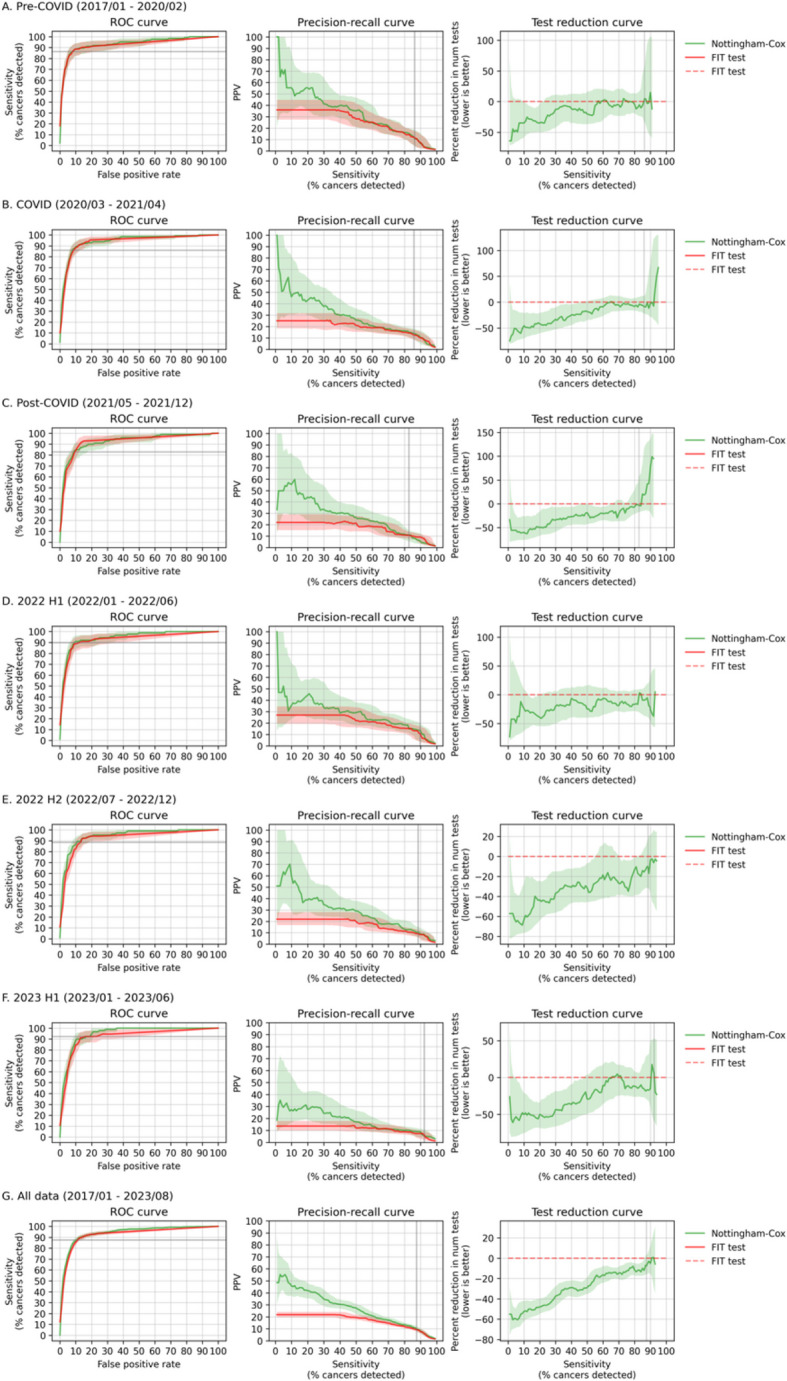
ROC, precision-recall and test reduction curves for the Nottingham models over the six time periods. Precision-recall curve shows how the positive predictive value (PPV) of the model changes as the risk score threshold is increased, so that the model captures fewer cancers but does so more confidently. Test reduction curve shows the percent reduction in number of positive tests (referrals) for the model relative to FIT test at the same level of sensitivity (i.e. when the model and FIT capture the same number of cancers). Percent reduction in number of positive tests can be computed from the precision-recall curve by the formula (ppv_fit/ppv_model—1) * 100, where ppv_fit is the PPV of FIT test and ppv_model is the PPV of the prediction model at a given level of sensitivity. Confidence intervals are 95% bootstrap percentile intervals

COLOFIT had higher *c*-statistic and average precision (AP) than FIT alone when evaluated on all data: *c*-statistics were 94.04 (model) and 92.36 (FIT); AP was 27.12 (model) and 16.81 (FIT). Estimates of the *c*-statistic and AP were also higher in each of the six time periods (Table 6). ROC curves of COLOFIT and FIT were similar (Fig. 5). Precision-recall curves showed that COLOFIT tended to have higher PPVs than FIT near the sensitivity of FIT ≥ 10 µg/g in 2022, consistent with observed reduction in referrals (‘test reduction curve’ in Fig. 5).

**Table 6:** Average precision and *c*-statistic for the COLOFIT model and the FIT test

Model	Prevalence of colorectal cancer (%)	Average precision (%)	c-statistic (%)
All data (2017/01–2023/08)			
FIT test	1.28	16.81 (14.98, 18.74)	92.36 (91.2, 93.43)
COLOFIT	1.28	27.12 (23.7, 30.88)	94.04 (93.09, 94.96)
Pre-COVID (2017/01–2020/02)			
FIT test	1.19	25.75 (19.99, 32.77)	92.49 (89.46, 95.33)
COLOFIT	1.19	33.97 (26.33, 43.21)	93.58 (90.88, 96.03)
COVID (2020/03–2021/04)			
FIT test	1.44	19.34 (15.0, 24.22)	93.63 (91.08, 95.64)
COLOFIT	1.44	29.81 (22.44, 38.99)	94.26 (92.03, 96.05)
Post-COVID (2021/05–2021/12)			
FIT test	1.32	17.2 (12.89, 22.68)	92.36 (89.11, 95.09)
COLOFIT	1.32	27.32 (20.02, 37.44)	92.68 (89.33, 95.43)
2022 H1 (2022/01–2022/06)			
FIT test	1.47	20.87 (14.9, 27.36)	92.66 (89.19, 95.54)
COLOFIT	1.47	28.64 (20.53, 39.21)	94.59 (92.14, 96.61)
2022 H2 (2022/07–2022/12)			
FIT test	1.39	16.89 (13.02, 22.12)	92.34 (89.41, 94.87)
COLOFIT	1.39	29.77 (22.59, 39.46)	94.58 (92.44, 96.39)
2023 H1 (2023/01–2023/06)			
FIT test	1.09	11.35 (8.41, 14.95)	91.81 (88.81, 94.58)
COLOFIT	1.09	18.56 (13.58, 26.98)	94.87 (93.46, 96.22)

Average precision is an estimator for area under the precision-recall curve and is high when the model has a high positive predictive value across all possible levels of detected cancers. Average precision of a model that predicts randomly is equal to prevalence of cancer in the dataset. Abbreviations: *FIT*, faecal immunochemical test

### Net benefit

Net benefit curves for recalibrated COLOFIT models revealed no clear differences between COLOFIT and FIT near probabilities of CRC corresponding to FIT 10 µg/g (see Additional File 12: Figure S12).

### Additional results

Diagnostic metrics at various risk thresholds and sensitivities are reported in Additional File 13: Table S13 and Additional File 14: Table S14. There were no strong differences in reduction in referrals pre- and post-buffer device adoption (Additional File 15: Figure S15). Demographics and bloods did not evenly contribute to reduction in referrals over time (Additional File 16: Figure S16).

## Discussion

### Main findings

We investigated whether COLOFIT could reduce the number of patients referred for urgent colorectal cancer investigation compared to the standard practice of referring patients with FIT ≥ 10 µg/g, without missing cancers compared to FIT. We found that COLOFIT would have led to an 8% reduction in referrals across the study period and between 23% reduction and 2% increase depending on the time period. This variation demonstrated the role of increasing testing rates and population characteristics on model performance.

### Strengths and limitations

We externally validated COLOFIT in a large real-world GP-requested FIT dataset of 51,477 patients, focussing on a clinically relevant metric: the change in the proportion of patients eligible for referral without missing colorectal cancer diagnoses compared to using FIT ≥ 10 µg/g alone. We evaluated COLOFIT overall and by dividing the seven-year study period into six independent periods to take into account changes in the tested population, including increases in the volume of testing, the sampling process, and changes in guidance to test patients with higher-risk symptoms during and after the COVID pandemic. Prospective usage of the COLOFIT model was also simulated.

The study has some limitations. A mixed reference standard including histopathology reports and diagnosis codes was used to identify cancers; thus, some cancers may have been missed due to coding errors, missed pathology reports, or lack of definitive investigation. However, it would be expected that if a colorectal cancer was the cause of the symptoms investigated, it would be diagnosed within 180 days of the FIT. Furthermore, increasing the length of follow-up to 365 days as in model derivation data had a minimal effect on the estimated reduction in referrals in time periods where both follow-up lengths were available. In addition, we did not evaluate how many advanced adenomas would have been missed or additionally detected if COLOFIT had been used over FIT. This was also important to the PPI group, but the OUH-FIT dataset does not currently have structured data about polyps. COLOFIT was validated only using the first FIT test for each patient for the results to be comparable to the model derivation study; subsequent studies should evaluate how it performs on subsequent FITs of the same patient.

### Comparison to the literature

The initial FIT positivity rate in this cohort is lower than in other primary care datasets cited by Booth et al. [6]. This suggests that the older OUH data has a higher percentage of low-risk patients and is less representative of the current practice, but this allowed us to investigate factors that influence COLOFIT’s performance.

**Fig. 6:**
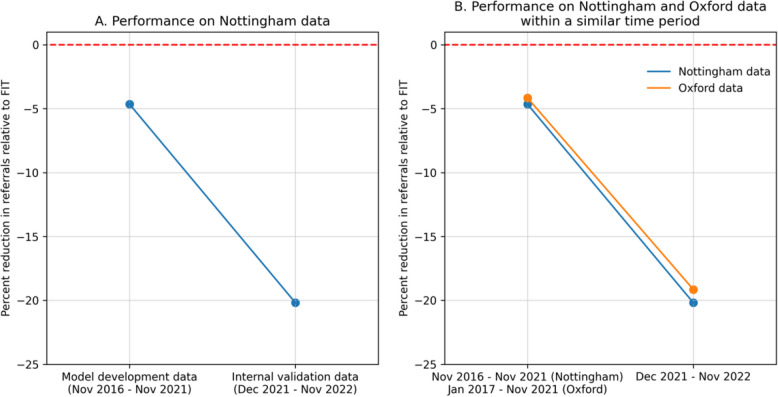
Percent reduction in the number of positive tests (referrals) compared to FIT ≥ 10 µg/g in Nottingham and OUH-FIT datasets. **A** Reduction in referrals in Nottingham model derivation and internal validation datasets. **B** Reduction in referrals in Nottingham and OUH-FIT datasets over similar time periods. Reduction in referrals for Nottingham data was computed using data from Table 4 in Crooks et al. [20], using a 0.64% risk score threshold as this captured a similar number of cancers as FIT ≥ 10 µg/g. For example, for every 100,000 FIT tests in Nottingham model derivation data, 21,383 patients tested positive for COLOFIT at the 0.64% threshold and 22,427 tested positive for FIT at the 10 µg/g threshold, yielding a (21,383–22,427)/22,427 × 100 = 4.7% reduction in referrals. Reduction in referrals in Oxford data was computed by finding a threshold that captures the same number of cancers as FIT ≥ 10 µg/g

COLOFIT’s performance over time varied significantly in the Nottingham dataset: COLOFIT yielded a 4.6% reduction in referrals in the model derivation data and a 20.2% reduction in the newer Nottingham internal validation data (Fig. 6A) [20]. In the OUH-FIT data, reductions were almost identical over the same period (Fig. 6B). This could be due to chance, an artefact of Crooks et al. [20] using risk score thresholds that did not capture as many cancers as FIT (COLOFIT captured 1.7% more cancers in model derivation data and 0.7% fewer in internal validation data), or indicate that Nottingham and Oxford underwent a similar population change.

Crooks et al. [20] reported that in East Lancashire data, 4793 patients (287 cancers) were above the COLOFIT 1% risk score threshold, while 6288 patients (295 cancers) had FIT ≥ 10 µg/g, representing a 23% reduction in referrals with eight cancers missed relative to FIT. However, if COLOFIT had been deployed to capture the same number of cancers as FIT, the reduction would have been smaller as a lower risk score threshold would be required. Reducing the threshold in the lower range of risk scores significantly increases referrals because most symptomatic patients have low risk scores. In COLOFIT derivation data, 83% of patients had risk scores below 1% [20].

High *c*-statistics for FIT-based models, as reported in a recent review [19] are only partly helpful when ascertaining the additional benefit of a FIT-based model as FIT alone has a high *c*-statistic. COLOFIT [20] was the only study we identified comparing the potential reduction in referrals to the current standard practice of FIT ≥ 10 µg/g at a similar sensitivity (see Additional File 17: Table S17, [20, 26, 37–44]). The other nine studies of FIT-based models have not observed improved performance relative to FIT at the same sensitivity or not evaluated the model and FIT at the same sensitivity [26, 37–44] (Additional File 18).

Three existing FIT-based models have been externally validated. The FAST (FIT, age and sex test) [37] was validated on UK primary care patients [38, 39] by computing reduction in referrals relative to all existing referrals but was not compared to FIT at the same sensitivity. The COLONPREDICT[40] and COLONOFIT [42] were validated on referred primary and secondary care patients and may not represent a relevant target population.

### Implications for research and practice

The Oxford Clinical Biochemistry laboratory switched from collecting faecal samples using standard stool pots to buffered test kits in June 2021. Switching to buffered kits led to a 4% increase in FIT positivity. FIT positivity then increased further in buffered samples. COLOFIT’s performance varied significantly over time in Nottingham data where only buffered kits were used, indicating that factors other than the sampling method can influence performance over time (Fig. 6A). It is likely that changes in the patient population towards testing a higher-risk symptomatic population (see Findings) contributed to the model reducing referrals by at least 17% in the later periods. However, more complete symptom information is needed to validate this association.

COLOFIT was validated using samples analysed on a different faecal haemoglobin analyser (HM-JACKarc) than during model development (OC sensor). The HM-JACKarc upper reporting range in the OUH dataset was 400 µg/g, while the OC sensor values in model development data reached 69,000 µg/g. Therefore, patients with FIT greater than 400 µg/g would obtain substantially lower COLOFIT risk scores using HM-JACKarc. A patient with an OC sensor result of 5000 µg/g could obtain a COLOFIT risk score of 46%, dropping to 26% if the value was capped at 400 µg/g (see Additional File 11). Whilst this drop is substantial, the clinical impact is probably minimal as 26% and 46% risk are above the level of risk used to guide urgent investigation.

COLOFIT performance varied over time as Oxfordshire rates of testing increased incorporating higher-risk symptomatic patients with rectal bleeding or blood in stool. We observed that a COLOFIT referral threshold may not be directly transferable if estimated using data from another setting, or in data from an earlier time period from the same setting if the tested population is changing. The optimal setting for COLOFIT implementation would be a population similar to the original Nottingham derivation population, a ‘pan-risk’ population including stable rates of both ‘low-risk’ and ‘high-risk’ symptoms of colorectal cancer and using a buffered stool collection kit. We offer three recommendations to inform the implementation of COLOFIT in new settings:

1. *Validation using local data would be the optimal approach where it is feasible and local data are readily available.* Before using COLOFIT to change patient care, the model would be validated in local data to identify the referral threshold that reduces referrals without missing cancers compared to FIT. This could be done retrospectively in systems where FIT has already been used, and the other variables in the model could be retrieved, or prospectively by running the model passively for a period of time while data accrues.
2. *COLOFIT can be implemented before local validation if data are not available to evaluate the model before implementation under the following conditions: FIT positivity is at least 17% and the colorectal cancer rate is near 1.3–1.6%. Without local validation, COLOFIT may miss a small number of cancers and might not reduce referrals relative to FIT.*
3. *Monitor COLOFIT performance following implementation.* COLOFIT performance may still vary over time with changes in the tested population. The optimal risk score threshold to indicate referral will correspondingly change over time. COLOFIT should be regularly revalidated in local data to understand changes in the tested population and to re-estimate a risk threshold as new data become available. This will reduce the likelihood of missed cancers whilst maintaining maximal reduction in referrals.

For future research, we recommend evaluating FIT-based models using reduction in referrals relative to FIT and provide a python pipeline to facilitate its use at https://github.com/tammandres/fitval. It should also be further studied which characteristics of the FIT population make demographics and bloods a useful rule-out test.

## Conclusions

COLOFIT has the potential to reduce the number of whole colon examinations without missing colorectal cancer diagnoses compared to FIT alone. Targeted validation is recommended prior to implementation using data from the local population to determine the COLOFIT risk threshold that is most likely to lead to a reduction in referrals without missing colorectal cancer diagnoses. Ongoing monitoring and validation over time would ensure that COLOFIT performance was optimised in line with changes in FIT use in the local population. COLOFIT can be implemented before local validation if data are not available to evaluate it, given that FIT positivity and CRC rate are near derivation data values, but the model may not reduce referrals and a small number of cancers may be missed.

## Supplementary Information

Additional File 1: Positive predictive value of the FIT test in primary care studies: Tables S1A-S1B, Figures S1A-S1B. Tab S1A – Primary care studies that report the PPV of the FIT test at threshold 10 µg/g. Tab S1B – Random-effects meta-analysis of PPV and number needed to scope for the FIT test at threshold 10 µg/g. Fig S1A – Prevalence of colorectal cancer plotted against positive predictive value of the faecal immunochemical test at the 10 µg/g threshold in primary care studies identified in Booth et al. meta-analysis. Fig S1B – Sensitivity and positive predictive value of the faecal immunochemical test at the 10 µg/g threshold in primary care studies identified in Booth et al. meta-analysis

Additional File 2: Extracting cancer status, TNM staging and symptoms from free text

Additional File 3: Source of cancer diagnosis

Additional File 4: Performance metrics computed in the external validation pipeline: Table S4. Tab S4 – Performance metrics for comparing prediction models against the FIT test

Additional File 5: Recalibration methods applied to the COLOFIT model

Additional File 6: Descriptive statistics for blood tests: Table S6. Tab S6 – Summary of selected blood test results

Additional File 7: Changes in patient population over time: Figures S7A-S7E. Fig S7A – FIT positivity and testing volume over time. Fig S7B – Correlation between FIT positivity and testing volume. Fig S7C – Time trends of FIT positivity and blood in stool. Fig S7D – Time trends of FIT positivity and all extracted clinical symptoms. Fig S7E – FIT positivity over time grouped by faecal sampling method

Additional File 8: Descriptive statistics for the patient cohort and blood tests over time: Tables S8A– S8B. Tab S8A – Descriptive statistics for the patient cohort over the six time periods. Tab S8B – Summaries of selected blood tests over the six time periods

Additional File 9: Reduction in referrals with 180-day and 365-day follow-ups: Tables S9A–S9B, Figure S9. Tab S9A – Cancers detected with 180-day and 365-day follow-ups. Tab S9B – Reduction in the number of referrals for the COLOFIT model relative to the FIT test with 180-day and 365-day follow-ups. Fig S9 – Reduction in the number of positive tests for the COLOFIT model relative to the FIT test with 180-day and 365-day follow-ups over time

Additional File 10: Calibration curves for recalibrated COLOFIT models: Figure S10.Figure S10 — Calibration curves for recalibrated COLOFIT models

Additional File 11: Effect of the FIT analytic device on COLOFIT calibration

Additional File 12: Decision curves for recalibrated COLOFIT models: Figure S12. Fig S12 —Decision curves

Additional File 13: Diagnostic metrics at selected levels of predicted risk: Table S13. Tab S13 – Performance metrics computed at selected levels of predicted risk for the recalibrated COLOFIT model and the Oxford FIT-spline model

Additional File 14: Diagnostic metrics at selected levels of sensitivity: Table S14. Tab S14 – Performance metrics computed at selected levels of sensitivity for the COLOFIT model and Oxford FIT-spline models

Additional File 15: Reduction in referrals before and after buffer device adoption: Figure S15. Fig S15 – Percent reduction in the number of positive tests and percent cancers missed compared to FIT ≥ 10 µg/g before and after buffer device adoption

Additional File 16: Contributions of age, sex and bloods to reduction in referrals: Figure S16. Fig S16 – Percent reduction in the number of positive tests and percent cancers missed compared to FIT ≥ 10 µg/g over time in OUH-FIT data, for the full and reduced COLOFIT-Cox models

Additional File 17: Evaluation of FIT-based risk prediction models in the literature: Table S17. Tab S17 – FIT-based colorectal cancer risk prediction models and their evaluation

Additional File 18: Reduction in referrals produced by FIT-based risk prediction models in the literature

## Data Availability

The analysed data cannot be shared as they are based on electronic health records. Code is available at [https://github.com/tammandres/fitval](https://github.com/tammandres/fitval).

## Abbreviations

AP: Average precision
CRC: Colorectal cancer
FIT: Faecal immunochemical test
GP: General practitioner
ICD-10: International Classification of Diseases, version 10
MCV: Mean cell volume
NICE: National Institute for Health and Care Excellence
NHS: National Health Service
O/E ratio: Observed/expected ratio
OUH: Oxford University Hospitals
PLT: Platelets
PPV: Positive predictive value
ROC: Receiver-operating characteristic
