# Supplementary material for: External validation of the COLOFIT colorectal cancer risk prediction model in the Oxford-FIT dataset: the importance of population characteristics and clinically relevant evaluation metrics
Source: BMC Med. 2025 Aug 27;23:503. doi: 10.1186/s12916-025-04339-w (PMC12392603; doi:10.1186/s12916-025-04339-w)
Supplement: Supplementary file 1 — Additional File 1: Positive predictive value of the FIT test in primary care studies: Tables S1A-S1B, Figures S1A-S1B. Tab S1A – Primary care studies that report the PPV of the FIT test at threshold 10 µg/g. Tab S1B – Random-effects meta-analysis of PPV and number needed to scope for the FIT test at threshold 10 µg/g. Fig S1A – Prevalence of colorectal cancer plotted against positive predictive value of the faecal immunochemical test at the 10 µg/g threshold in primary care studies identified in Booth et al. meta-analysis. Fig S1B – Sensitivity and positive predictive value of the faecal immunochemical test at the 10 µg/g threshold in primary care studies identified in Booth et al. meta-analysis [file 12916_2025_4339_MOESM1_ESM.pdf]

S1. POSITIVE PREDICTIVE VALUE OF THE FIT TEST IN PRIMARY CARE STUDIES

The faecal immunochemical test (FIT), when used at the NICE-recommended threshold of 10 μg/g, is known to have about 90% sensitivity for colorectal cancer in the primary care setting according to a recent meta-analysis by Booth et al [6]. However, what is the percentage of cancers among FIT positive patients, the positive predictive value (PPV) of FIT? And what is the number of FIT positive patients that need to be examined to detect one cancer, the number needed to scope (NNS)? Booth et al [6] only provided summary sensitivity and specificity. Here we summarise PPV and NNS in two ways: a meta-analysis of studies identified by Booth et al; and back-computation of PPV and NNS from the summary sensitivity and specificity reported by Booth et al.

Included studies. We considered primary care studies (*n* = 11) identified in Table 2 of Booth et al meta analysis [6] (Table S1A). We looked up the original articles to identify the number of patients testing positive for FIT at the 10 μg/g threshold and the number of colorectal cancers among FIT positives. For Chapman et al [7] and Nicholson et al [5, 15] the number of FIT positive patients and cancers was inferred using the reported number of cancers, sensitivity and PPV. For Pin Vieito et al [16], the number of FIT positive patients was inferred from the percentage of FIT positive patients, and the number of cancers and number of FIT positive cancers was inferred from sensitivity and PPV. Two studies reported more than one estimate of PPV: Chapman et al reported results for two different FIT sensors; Pin Vieto et al reported PPV for patients with altered bowel habit and for patients unstratified by symptom category. We only included the unstratified estimate from Pin Vieto et al. To avoid double counting, we computed the summary PPV and NNS twice, by including only the OC or the HM-JACKarc (HMJ) sensor results from Chapman et al. Booth et al also reported the Tier of the study: in Tier 1 studies, colorectal cancer was detected using colonoscopy; in Tier 2 studies, a mixed reference standard consisting of endoscopy and registry follow-up was used. We computed the summary PPV and NNS for all studies, and separately for Tier 1 and Tier 2 studies.

Meta-analysis of PPV. Summary PPV was estimated across the studies using a random-effects meta-analysis based on the number of FIT positive patients and the number of cancers among FIT positives reported for each study. We employed the *metaprop* function from the *meta* package (v7.0-0) in R (v4.3.2) using default settings that fits a logistic regression model with a random intercept to provide a summary estimate for proportions. NNS was computed as the reciprocal of PPV (1 / PPV) when PPV is on the proportion scale.

Back-computation of PPV. Summary PPV was also computed from the meta-analytic estimates of sensitivity and specificity reported by Booth et al, separately for Tier 1 and Tier 2 studies, using the average prevalence of Tier 1 and Tier 2 studies. The formula for PPV is *sensitivity* * *prevalence* / [*sensitivity* * *prevalence* + (1 – *specificity*) * (1 – *prevalence*)]. The sensitivity and specificity reported by Booth et al were 91.1% (85.7, 94.5) and 71.6% (57.7, 82.2) for Tier 1 studies when including the OC sensor result from Chapman et al; 90.1% (83.9, 94.1) and 72.6% (58.6, 83.3) for Tier 1 studies when including the HM-JACKarc sensor result from Chapman et al; and 88.8% (84.6, 92.0) and 85.7% (82.3, 88.5) for Tier 2 studies.

Results. Sample size of the 11 primary care studies varied from 238 to 13,042 patients (median 3,890). The observed PPV and prevalence of cancer in each study is shown in Figure S1A; sensitivity and PPV are shown in Figure S1B. In all seven Tier 2 studies, prevalence was below 3% (average 1.7%), whereas Tier 2 studies had prevalence between 1.2% and 5.2% (average 3.7%). The random-effect estimates for PPV were 9% (both Tiers, Chapman-OC), 9.1% (both Tiers, Chapman-HMJ), 10.2% (Tier 1, Chapman-OC), 10.4% (Tier 1, Chapman-HMJ), and 8.3% (Tier 2). The random-effect estimates for NNS were 11.1 (both Tiers, Chapman-OC), 11 (both Tiers, Chapman-HMJ), 9.8 (Tier 1, Chapman-OC), 9.6 (Tier 1, Chapman-HMJ), and 12.1 (Tier 2). The back-computation estimates for PPV were 11.8% (Tier 1, Chapman-OC), 11.5% (Tier 1, Chapman-HMJ), and 9.7% (Tier 2). The back-computation estimates for NNS were 8.5 (Tier 1, Chapman-OC), 8.7 (Tier 1, Chapman-HMJ), and 10.3 (Tier 2). Please see Table S1B for confidence intervals.

Conclusion. Across all 11 primary care studies, the number needed to scope was 11, indicating that about one in 11 patients testing positive for FIT at the 10 μg/g threshold had colorectal cancer. This was based on a combined sample of at least 11,000 positive FITs. If one would only include the four

studies with colonoscopy as the reference standard, about one in 10 patients testing positive for FIT had cancer. The back-computation estimates of NNS were lower, but similar: about 1 in 9 patients had cancer according to Tier 1 studies assuming a fixed prevalence of 3.7%, and 1 in 10 had cancer according to Tier 2 studies assuming a prevalence of 1.7%.

Acknowledgments. We thank Professor Richard Stevens for a helpful discussion about the relationship between prevalence and PPV and meta-analysis techniques.

Table S1A. Primary care studies that report the PPV of FIT test at threshold 10 μg/g. Data is taken from Table 2 of Booth et al meta-analysis [6], except the number of cancers, the number of patients that tested positive for FIT, and the number of cancers among FIT positive patients

Study	Tier	Reference Test	FIT sensor	Presenting symptoms	Num patients	Num cancers	Cancer prevalence	Num FIT ≥ 10	Num cancer FIT ≥ 10	Sensitivity	PPV
Chapman, 2021 [7]	1	Colonoscopy, CT colonography	OC	high-risk	732	38	5.2	214**	34**	89.5	15.9
Chapman, 2021 [7]	1	Colonoscopy, CT colonography	HM-JACKarc	high-risk	732	38	5.2	185**	32**	84.2	17.3
Khasawneh, 2020 [8]	1	CT colonography	OC	cibh	5818	72	1.2	1170	64	88.9	5.5
McSorley, 2020 [9]	1	Colonoscopy	HM-JACKarc	high-risk	4841	266	5.5	2675	252	94.7	9.4
Mowat, 2016 [10]	1	Colonoscopy	OC	unstratified	750	28	3.7	176	25	89.3	14.2
Bailey J, 2021 [11]	2	registry f/up	OC	high-risk	13042	227	1.7	2554	209	92.1	8.2
Bailey S, 2021 [12]	2	registry f/up	HM-JACKarc	low-risk	3890	51	1.3*	618	43	84.3	7
Juul, 2018 [13]	2	Colonoscopy, registry f/up	OC	unstratified	3462	54	1.6	540	51	94.4	9.4
Mowat, 2021 [14]	2	Colonoscopy, CT colonography, CT, flexible sigmoidoscopy, registry f/up	HM-JACKarc	unstratified	5381	105	2	1177	91	86.7	7.7
Nicholson, 2019 [15]	2	Colonoscopy, CT colonography, Registry f/up	HM-JACKarc	low-risk	238	7	2.9*	28**	6**	85.7	21.4
Nicholson, 2020 [5]	2	Registry f/up	HM-JACKarc	unstratified	9896	105	1.1	945**	95**	90.5	10.1
Pin Vieito, 2020 [16]	2	Registry f/up	OC	unstratified	5623	81***	1.4	945***	65***	80.2	6.9

Note. *For these studies, the prevalence of cancer differs from data reported in Booth et al, but is likely to be correct as prevalence was recomputed using reported sample size and number of cancers. For Bailey S (2021) Booth et al reported 1.1 prevalence but this is the prevalence of cancer for FIT positive patients. Similarly, for Nicholson (2020) Booth et al reported 7 as prevalence but this is the number not percentage of cancers. **These numbers were inferred from sensitivity and PPV reported in the studies. ***These numbers were inferred from the proportion of FIT positive tests, sensitivity and PPV reported in the study.

Table S1B. Random-effects meta-analysis of PPV and number needed to scope for the FIT test at threshold 10 μg/g, based on studies reported in Booth et al meta-analysis [6]

Tier*	Chapman (2021) FIT sensor	Num studies	Total num FIT ≥ 10	Total num cancers FIT ≥ 10	PPV (95% CI), meta-analysis	NNS (95% CI), meta-analysis	PPV (95% CI), back-compute	NNS (95% CI), back-compute
Both	OC	11	11042	935	9 (7.5, 10.8)	11.1 (9.2, 13.4)	Not available	Not available
Both	HM-JACKarc	11	11013	933	9.1 (7.5, 11)	11 (9.1, 13.4)	Not available	Not available
1	OC	4	4235	375	10.2 (6.7, 15.1)	9.8 (6.6, 14.9)	11.8 (7.6 ,18.6)	8.5 (5.4 ,13.2)
1	HM-JACKarc	4	4206	373	10.4 (6.7, 15.8)	9.6 (6.3, 14.9)	11.5 (7.6 ,17.7)	8.7 (5.6 ,13.2)
2	-	7	6807	560	8.3 (7.5, 9.1)	12.1 (11, 13.4)	9.7 (7.6 ,12.2)	10.3 (8.2 ,13.1)

Note. *In Tier 1 studies, colonoscopy was used as a reference standard for detecting colorectal cancers; in Tier 2 studies, a mixed reference standard was used (based on Booth et al). PPV – positive predictive value, reported on a percentage scale; NNS – number needed to scope, the number of FIT positive patients that need to be examined to detect one colorectal cancer. “meta-analysis” refers to random effects meta-analysis based on the studies reported by Booth et al; “back-compute” refers to estimates of PPV and NNS computed from estimates of sensitivity and specificity reported by Booth et al when using average prevalence for Tier 1 and Tier 2 studies, respectively.

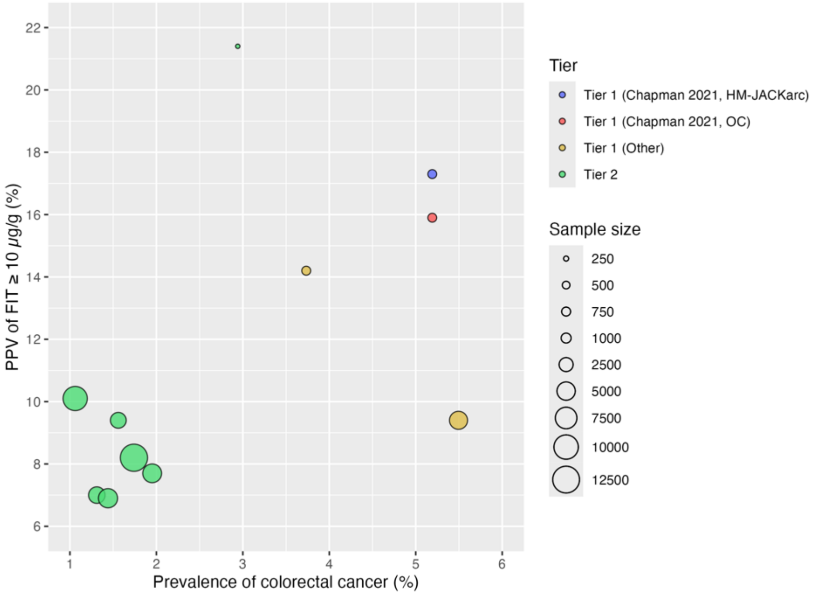

Figure S1A. Prevalence of colorectal cancer plotted against positive predictive value (PPV) of the faecal immunochemical test at the 10 μg/g threshold in primary care studies identified in Booth et al meta-analysis [6]. Tier 1 studies used colonoscopy as the reference standard; Tier 2 studies used a mixed reference standard. A study by Chapman et al [7] reported results for two different FIT sensors (HM-JACKarc and OC): these are shown in different colours to avoid double counting the study.

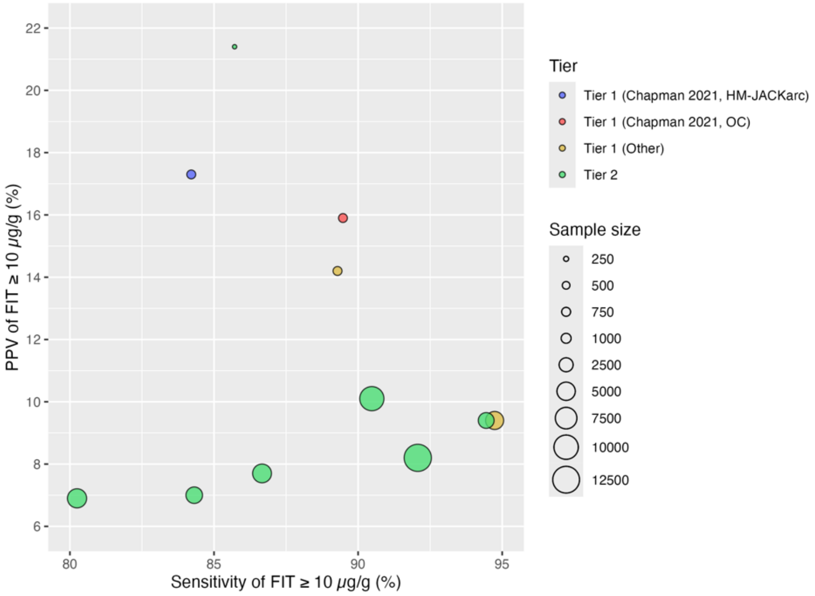

Figure S1B. Sensitivity and positive predictive value (PPV) of the faecal immunochemical test at the 10 μg/g threshold in primary care studies identified in Booth et al meta-analysis [6]. Tier 1 studies used colonoscopy as the reference standard; Tier 2 studies used a mixed reference standard. A study by Chapman et al [7] reported results for two different FIT sensors (HM-JACKarc and OC): these are shown in different colours to avoid double counting the study.
