# Supplementary material for: External validation of the COLOFIT colorectal cancer risk prediction model in the Oxford-FIT dataset: the importance of population characteristics and clinically relevant evaluation metrics
Source: BMC Med. 2025 Aug 27;23:503. doi: 10.1186/s12916-025-04339-w (PMC12392603; doi:10.1186/s12916-025-04339-w)
Supplement: Supplementary file 2 — Additional File 2: Extracting cancer status, TNM staging and symptoms from free text [file 12916_2025_4339_MOESM2_ESM.pdf]

S2. EXTRACTING CANCER STATUS, TNM STAGING AND SYMPTOMS FROM FREE TEXT

Colorectal cancer. Presence of primary colorectal cancer was extracted from free text pathology reports using a regex-based algorithm developed by the first author in collaboration with colorectal cancer clinicians Dr Helen Jones and Dr Neel Doshi. The algorithm first used a comprehensive set of keywords related to tumours and tumour sites to extract all potential sentences that describe colorectal cancer. Additional pattern matching rules were applied to the words on the left and right side of each extracted keyword, similar to the ConText algorithm [27]. This excluded keywords that were negated ('no colorectal cancer'), general ('in patients with colorectal cancer'), historic ('history of colorectal cancer), or that described metastasis, recurrence or treatment response. The algorithm is described in detail in a soon-to-be-published manuscript [29], and initial validation on OUH-FIT data showed it achieved 92% PPV and 97% sensitivity for retrieving pathology reports that describe current CRC among all main pathology reports of CRC patients (i.e. among reports that describe primary CRC, other primary tumours, other nonprimary tumours with CRC in clinical history, or other pathologies, but excluding the less-relevant supplementary gene testing reports). However, 92% PPV still means that about 8% of extracted matches were false positives. To further refine the performance, all extracted keywords along with their left and right context were 'semi-manually' processed. This involved looking for very specific patterns on the left and right side that indicated a current colorectal sample (e.g. 'biopsy – colon', or 'site: sigmoid colon'), and manually reviewing all sentences that matched this pattern. These steps are encoded in a python script that can be used to reproduce the results.

Please note a subtlety when interpreting the PPV and sensitivity of the CRC algorithm. The algorithm was quantitatively evaluated for identifying pathology reports that describe current CRC among the various other pathology reports of CRC patients, so it has not strictly been evaluated when run on the reports of CRC patients *and* FIT-testing patients without CRC. However, this does not change the quality of identifying CRC reports in the current analysis, because (1) the algorithm only targets reports that mention tumours and colorectal sites, and patients with a FIT test will predominantly have these reports only if they have or had CRC (i.e. when they already belong to the population on which the algorithm was evaluated); and (2) all sentences extracted by the algorithm were semi-manually reviewed to ensure they strictly described current CRC and were not general or false positive statements.

TNM staging. T-staging scores were extracted from histopathology and imaging reports using another regex-based algorithm developed by the first author in collaboration with colorectal cancer clinician Dr Helen Jones that achieved at least 94% PPV and 91% sensitivity for retrieving current explicit TNM stages [29]. Lack of sensitivity was mainly due to reports that described historical staging or where staging could be inferred from text but was not reported in customary letters and numbers of the TNM staging system. The algorithm first extracted all phrases that contain TNM staging given in letters and numbers, such as "pT1 (<comment>), N0, M0", allowing for common variations in how the staging may be reported (e.g. mis-spelling 0 as O, writing the letters in different orders, not reporting all TNM categories, having extensive comments between TNM letters, reporting multiple values for a single TNM category such as 'T1/2' etc). Additional rules were used to exclude false positive matches, for example where "T1" represents the T1-vertbra not TNM staging. Individual staging values were then extracted from the phrases. The algorithm is described in detail in a soon-to-be-published manuscript.

Clinical symptoms. Clinical symptoms, such as abdominal pain, were extracted from free text comments associated with the FIT test request. We started from a comprehensive set of keywords used by Withrow et al [26] and encoded these into more general regular expressions to better capture variations in reporting. Unlike pathology reports, the clinical details associated with FIT requests often contained spelling errors. A python spell checking program written by the computer scientist Peter Norvig (https://norvig.com/spell-correct.html) was used to identify spelling variations for all symptom keywords, such as 'abd pain', 'adb pain', 'abod pain' for abdominal pain, and these variations were incorporated into the regular expressions that extracted symptoms. Additional patterns were used to exclude negated keywords, such as 'no abd pain'. The results were validated by examining a random sample of all extracted symptom keywords along with their left and right

context, as well as clinical details where no symptoms were detected. The clinical details associated with FIT requests were short (< 200 characters), which made inspection of the results easier.
