# Supplementary material for: External validation of the COLOFIT colorectal cancer risk prediction model in the Oxford-FIT dataset: the importance of population characteristics and clinically relevant evaluation metrics
Source: BMC Med. 2025 Aug 27;23:503. doi: 10.1186/s12916-025-04339-w (PMC12392603; doi:10.1186/s12916-025-04339-w)
Supplement: Supplementary file 3 — Additional File 3: Source of cancer diagnosis [file 12916_2025_4339_MOESM3_ESM.pdf]

S3. SOURCE OF CANCER DIAGNOSIS

In the OUH-FIT dataset, cancers were identified using histopathology reports and ICD-10 diagnosis codes (see Methods in main text). A histopathology report describing colorectal cancer was available for 582 (88.3%) of the 659 cancer patients within 180 days of the date of colorectal cancer that was used in the analysis. An inpatient ICD-10 colorectal cancer diagnosis code but no histopathology report was available for 75 (11.4%) of patients within 180 days of the date of colorectal cancer. Finally, 2 (0.3%) patients had an outpatient ICD-10 colorectal cancer diagnosis code without a histopathology report or an inpatient diagnosis code.

Therefore, most cancer patients included in the study had histopathological evidence of colorectal cancer, and if not, most still had an inpatient diagnosis code for cancer which can be considered a more reliable source of information than outpatient diagnosis code. Only two patients were classified as cancer cases based on outpatient data alone.
