# Supplementary material for: External validation of the COLOFIT colorectal cancer risk prediction model in the Oxford-FIT dataset: the importance of population characteristics and clinically relevant evaluation metrics
Source: BMC Med. 2025 Aug 27;23:503. doi: 10.1186/s12916-025-04339-w (PMC12392603; doi:10.1186/s12916-025-04339-w)
Supplement: Supplementary file 4 — Additional File 4: Performance metrics computed in the external validation pipeline: Table S4. Tab S4 – Performance metrics for comparing prediction models against the FIT test [file 12916_2025_4339_MOESM4_ESM.pdf]

S4. PERFORMANCE METRICS COMPUTED IN THE EXTERNAL VALIDATION PIPELINE

We created a python script that computes both the core metrics of model performance (reduction in referrals relative to FIT) and other common metrics, given an input dataset of risk scores (‘y_pred’) and colorectal cancer indicators (‘y_true’) for each patient. Table S4 summarises these metrics.

Table S4. Performance metrics for comparing prediction models against the FIT test

Metric	Description
A. Clinically relevant ‘core’ metrics	
Percent reduction in the number of positive tests	Percent reduction in the number of patients that test positive when the prediction model is used instead of the FIT test at threshold ≥ 10 μg/g, and when the model is used at a threshold that captures the same number of cancers as FIT ≥ 10 μg/g*. It is computed as ((pos_fit – pos_model) / pos_fit) * 100, where pos_fit is the number of patients that test positive according to FIT ≥ 10 μg/g, and pos_model is the number that test positive according to the model. *This is a proxy for the reduction in number of referrals if all patients with FIT ≥ 10 μg/g were referred.*
Test reduction curve	Plots percent reduction in the number of positive tests compared to FIT at each level of sensitivity. It is derived from the precision-recall curve: at each level of sensitivity, the percent reduction is given by (ppv_fit/ppv_model - 1) * 100.
B. Discrimination metrics	
c-statistic	Concordance statistic, estimates the probability that a randomly chosen patient with cancer is assigned a higher risk score than a randomly chosen patient without cancer; equivalent to area under the ROC curve; *c*-statistic of a prediction model that assigns risk scores randomly is equal to 0.5.
Average precision (AP)	Estimates area under the precision-recall curve [30]. The area is higher when the model has high PPV over all possible classification thresholds (i.e. over all possible sensitivities). AP of a prediction model that assigns risk scores randomly is equal to the proportion of cancer cases in the dataset.
Precision-recall (PR) curve	Plots sensitivity (x-axis) against positive predictive value (y-axis). It can be more informative than ROC-curve when the proportion of patients with cancer is low in the dataset (as is often the case) [31].
Receiver-operating characteristic (ROC) curve	Plots false positive rate (x-axis) against sensitivity (y-axis). False positive rate is 1 - specificity.
Basic metrics at predefined levels of sensitivity	Positive predictive value, negative predictive value, specificity, number of positive and negative tests, number of detected and missed cancers, at selected levels of sensitivity: 80%, 85%, 90%, 95%, 99%.
Basic metrics at predefined levels of risk (predefined probabilities of colorectal cancer)	Positive predictive value, negative predictive value, specificity, number of positive and negative tests, number of detected and missed cancers, at selected levels of predicted risk: 0.5, 1%, 2%, 3%, 4%, 5%, 10%, 20%. NB: these are meaningful only for calibrated models.
C. Calibration metrics	
The observed-expected (O/E) ratio	Ratio between the observed proportion of cancer cases in the data and the average predicted risk of cancer according to the model. If the ratio is 1, the model does not over- or underpredict the total number of patients with cancer [23].
Logistic intercept and slope	Intercept and slope of a logistic regression model that predicts the occurrence of cancer from the logits of model's predicted probabilities. A slope of 1 indicates that a unit increase in logits of predicted probabilities is associated with a unit increase in logits of true probabilities [23]. Observed-expected ratio of 1 together with a slope of 1 constitutes a minimum required “weak” level of calibration [23].
Smooth calibration curve	Observed cancer events were regressed against predicted probabilities of cancer using locally weighted scatterplot smoothing (LOWESS) [32, 33].
D. Net benefit metrics	
Decision (net benefit) curve	Plots predicted probability of cancer (x-axis) against net benefit (y-axis). Net benefit is computed as tpr - fpr * p/(1-p), where tpr is the true positive rate (sensitivity), fpr is the false positive rate (1 - specificity), and p is the predicted probability of cancer according to the model [34]. NB: meaningful for calibrated models only.

Note. *If a threshold cannot be chosen for the model that yields the same sensitivity as FIT ≥ 10 μg/g, the precision-recall curve of the model is interpolated using the method of Davis and Goadrich [31], and percent reduction in number of tests is computed based on the ratio of PPVs: (ppv_fit / ppv_model - 1) * 100.
