# Supplementary material for: External validation of the COLOFIT colorectal cancer risk prediction model in the Oxford-FIT dataset: the importance of population characteristics and clinically relevant evaluation metrics
Source: BMC Med. 2025 Aug 27;23:503. doi: 10.1186/s12916-025-04339-w (PMC12392603; doi:10.1186/s12916-025-04339-w)
Supplement: Supplementary file 5 — Additional File 5: Recalibration methods applied to the COLOFIT model [file 12916_2025_4339_MOESM5_ESM.pdf]

S5. RECALIBRATION METHODS APPLIED TO THE COLOFIT MODEL

We found that COLOFIT was not calibrated in OUH-FIT data and attempted to recalibrate it using three methods.

- *Constant multiplication*. FIT values were multiplied by 3.5 before applying the model. The faecal samples were analysed using different sensors in Nottingham and Oxford (HM-Jack in Oxford, OC in Nottingham), and in a separate Nottingham dataset the OC results were 3.5 times higher than HM-Jack results.
- *Quantile transformation*. FIT values in Oxford data that fell within each quantile bin were replaced with values from the Nottingham derivation data that fell within the same bin.
- *Logistic recalibration (Platt scaling).* The probabilities returned by the model were transformed, using a secondary logistic model that predicts the outcome (cancer) from the logits of predicted probabilities.
