# Supplementary material for: External validation of the COLOFIT colorectal cancer risk prediction model in the Oxford-FIT dataset: the importance of population characteristics and clinically relevant evaluation metrics
Source: BMC Med. 2025 Aug 27;23:503. doi: 10.1186/s12916-025-04339-w (PMC12392603; doi:10.1186/s12916-025-04339-w)
Supplement: Supplementary file 6 — Additional File 6: Descriptive statistics for blood tests: Table S6. Tab S6 – Summary of selected blood test results [file 12916_2025_4339_MOESM6_ESM.pdf]

S6. DESCRIPTIVE STATISTICS FOR BLOOD TESTS

Patients with colorectal cancer were also more likely to have low haemoglobin (52.4% *vs* 33.4%; $c^2_{(1)}$ = 105.1, $p < 0.001$), elevated platelets (21.1% *vs* 10.5%; $c^2_{(1)}$ = 77.5, $p < 0.001$), high white cell counts (16.1% *vs* 12.5%; $c^2_{(1)}$ = 7.8, $p < 0.01$), low mean cell haemoglobin (35.2% *vs* 15.5%; $c^2_{(1)}$ = 191.0, $p < 0.001$), and low mean cell volume (18.7% *vs* 6.3%; $c^2_{(1)}$ = 164.2, $p < 0.001$) (Table S6).

Table S6. Summary of selected blood test results

Characteristic	No colorectal cancer	Colorectal cancer
Number of patients	50818	659
Haemoglobin (HGB), g/L		
Median (25th, 75th)	134.0 (122.0, 144.0)	126.0 (109.0, 140.0)
Min and max	50.0, 226.0	53.0, 184.0
Not known	Not Available	-
Low haemoglobin	16957 (33.4%)	345 (52.4%)
Normal haemoglobin	41263 (81.2%)	400 (60.7%)
Platelets (PLT), 10^9/L		
Median (25th, 75th)	266.0 (224.0, 316.0)	305.0 (244.0, 369.0)
Min and max	9.0, 1241.0	93.0, 920.0
High platelets	5318 (10.5%)	139 (21.1%)
Normal platelets	48715 (95.9%)	590 (89.5%)
White cells (WBC), 10^9/L		
Median (25th, 75th)	6.7 (5.6, 8.1)	7.5 (6.3, 9.1)
Min and max	1.3, 237.5	3.6, 24.6
High white cells	6337 (12.5%)	106 (16.1%)
Normal white cells	49600 (97.6%)	618 (93.8%)
Mean cell haemoglobin (MCH), pg/cell		
Median (25th, 75th)	30.1 (28.7, 31.3)	28.7 (26.0, 30.5)
Min and max	13.8, 49.6	12.5, 38.7
Not known	27 (0.1%)	-
Low MCH	7865 (15.5%)	232 (35.2%)
Normal MCH	45474 (89.5%)	496 (75.3%)
Mean cell volume (MCV), fl		
Median (25th, 75th)	91.7 (88.1, 95.1)	89.1 (83.7, 93.2)
Min and max	48.1, 137.1	55.0, 121.6
Low MCV	3206 (6.3%)	123 (18.7%)
Normal MCV	48899 (96.2%)	581 (88.2%)

Note. Normal, high, and low values for these bloods were defined as in Withrow et al [26]. Low HGB: < 130 g/L for males, < 120 g/L for females. High PLT: > 400 * 10^9/L. High WBC: > 11 * 10^9/L. Low MCH: < 27.4 pg/cell. Low MCV: < 80 fl. Rows where the result is shown as “Not available” had a count less than 10. Percentages of low/normal/high results do not add to 100, because these were defined as the presence of at least one low/normal/high result within a [-365, 14] day period around the first FIT test result.
