# Supplementary material for: External validation of the COLOFIT colorectal cancer risk prediction model in the Oxford-FIT dataset: the importance of population characteristics and clinically relevant evaluation metrics
Source: BMC Med. 2025 Aug 27;23:503. doi: 10.1186/s12916-025-04339-w (PMC12392603; doi:10.1186/s12916-025-04339-w)
Supplement: Supplementary file 7 — Additional File 7: Changes in patient population over time: Figures S7A-S7E. Fig S7A – FIT positivity and testing volume over time. Fig S7B – Correlation between FIT positivity and testing volume. Fig S7C – Time trends of FIT positivity and blood in stool. Fig S7D – Time trends of FIT positivity and all extracted clinical symptoms. Fig S7E – FIT positivity over time grouped by faecal sampling method [file 12916_2025_4339_MOESM7_ESM.pdf]

S7. CHANGES IN PATIENT POPULATION OVER TIME

Increased FIT positivity. Since September 2021, there has been an increasing trend in the proportion of patients testing positive for FIT at the 10 μg/g threshold: FIT positivity has increased from 11.7% in September 2021 to 18.8% in February 2024 (Figure S7A). Before September 2021, the median monthly FIT positivity rate was 9.5%, and the 5th and 95th percentiles were 6.3% and 12.5%.

Increased monthly number of tests. The number of FIT tests done each month has also increased from less than 411 tests before July 2020 to usually more than 1,400 monthly tests since April 2023 (Figure S7A). Furthermore, the number of tests done each month has been correlated with the *proportion* of patients testing positive for FIT at the 10 μg/g threshold: since July 2020, when the number of tests started increasing, the monthly number of tests explained 52% of variance in monthly proportion of positive tests; since September 2021, when FIT positivity started increasing, it explained 29% (Figure S7B). These correlations are not due to more tests being done each month, as the total number of tests is correlated with the *proportion* of patients testing positive. This indicates that not only more patients are being tested each month, but patients who are more likely to test positive are being tested.

Increases in patients with blood in stool. While the proportion of patients testing positive for the FIT test each month has been increasing, the proportion of patients presenting with blood in stool (which includes rectal bleeding) and rectal bleeding only, has increased at a similar rate as FIT positivity (Figure S7C). There was no obvious trend in other clinical symptoms (Figure S7D). However, the increase in patients with these clinical symptoms does not fully explain increases in FIT positivity, because not all patients with these symptoms will have a positive FIT. In the whole dataset, the probability of a positive FIT is 21.1% for patients with blood in stool and 10.9% for patients without blood in stool; and 21.7% for rectal bleeding and 11.3% for no rectal bleeding.

Increases in FIT positivity among tests done in the buffer device. The Oxford clinical biochemistry laboratory switched to using buffer devices rather than stool pots for FIT testing around July 2021. There is concern that when FIT samples are returned in a stool pot, haemoglobin degradation may occur during the time that the sample is transferred to the laboratory. Since April 2022, there is data for how many tests were collected in stool pots rather than buffer devices. This shows that the proportion of patients with a positive FIT result has been increasing even among patients who did their FITs with the buffer device, from 11.3% in April 2022 to 19.0% in February 2024 (Figure S7E). This rules out the argument that increasing FIT positivity is related to the adoption of the buffer device. Less than 4% increase in FIT positivity can be attributed to the buffer device – before the buffer devices were adopted, FIT positivity was 10% in June 2021; in May 2022, when data about buffer devices was available, FIT positivity was 14%. If one interpolated the FIT positivity in buffer devices back from May 2022 to June 2021, the increase in positivity would be smaller than 4%.

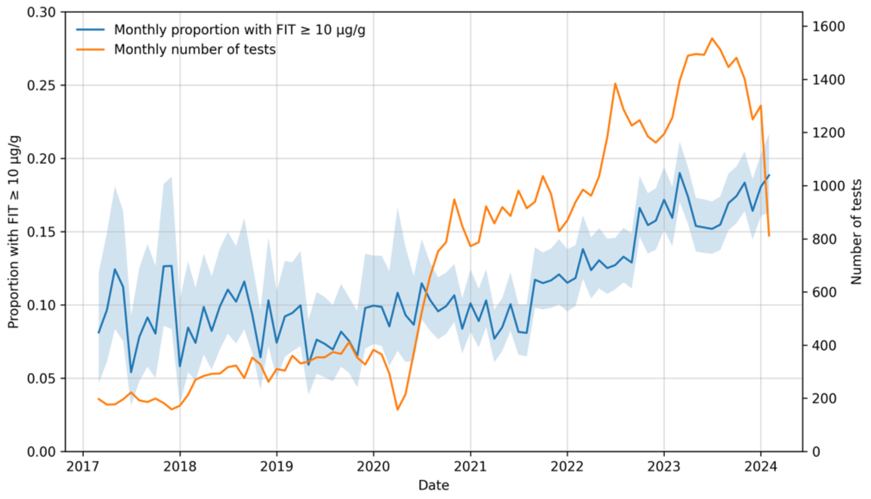

Figure S7A. FIT positivity and testing volume over time. Proportion of patients testing positive for FIT at the 10 µg/g threshold each month (left axis, blue line), and number of FITs recorded each month (right axis, orange line) are shown. Shaded areas display 95% Wilson confidence intervals. Proportions were computed for months with at least 10 FIT positive patients (all months except Jan and Feb 2017).

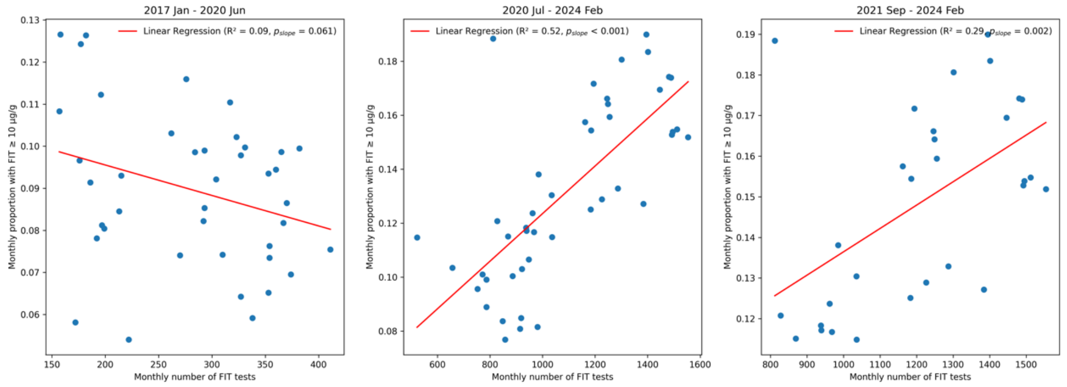

Figure S7B. Correlation between FIT positivity and testing volume. Monthly number of FIT tests plotted against monthly proportion of patients testing positive for the FIT test at the 10 µg/g threshold. Graphs are shown for three time periods: 2017 January to 2020 June (left) – the period before monthly number of FIT tests started increasing; 2020 July to 2024 February (middle) – the period when monthly number of FIT tests was increasing; 2021 September to 2024 February (right) – the period when both monthly FIT positivity and monthly number of tests were increasing. R^2 is the coefficient of determination; p_{slope} is the p-value of the regression slope.

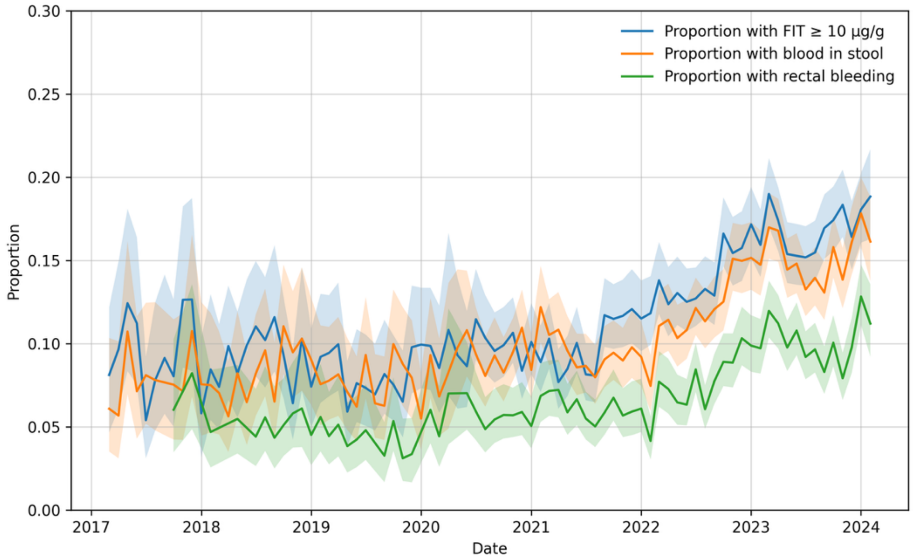

Figure S7C. Time trends of FIT positivity and blood in stool. Proportion of patients testing positive for FIT at the 10 µg/g threshold each month, and proportion of patients presenting with blood in stool (which includes rectal bleeding) or with rectal bleeding only each month. Shaded areas show 95% Wilson confidence intervals. Proportions were computed only for months with at least ten positive events. Please note that increases in blood symptoms do not fully explain increases in FIT positivity, because not all patients with these symptoms will have a positive FIT. Chances of a positive FIT were approximately 21% for patients with blood symptoms and 10% for patients without.

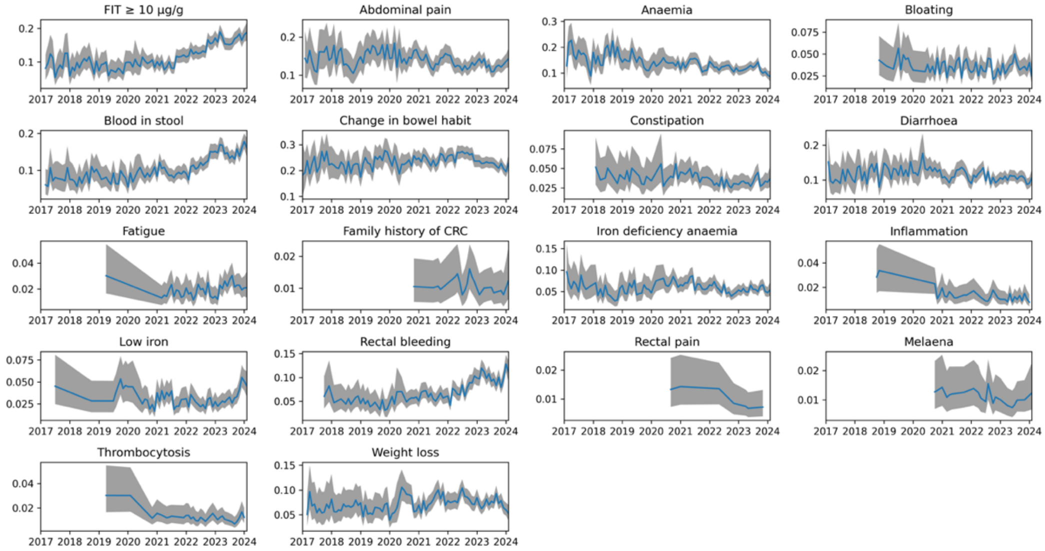

Figure S7D. Time trends of FIT positivity and all extracted clinical symptoms. Proportion of patients testing positive for the FIT test at the 10 µg/g threshold each month is shown in the upper left corner; proportion of patients presenting with each of the clinical symptoms recorded in the FIT test request is shown in the remaining panels. Shaded areas show 95% Wilson confidence intervals. If a month had less than 10 records for clinical symptoms, it was excluded as otherwise the proportion calculation would be unreliable. Rectal and abdominal mass are not shown in this figure, as there were less than 10 patients presenting with these symptoms each month.

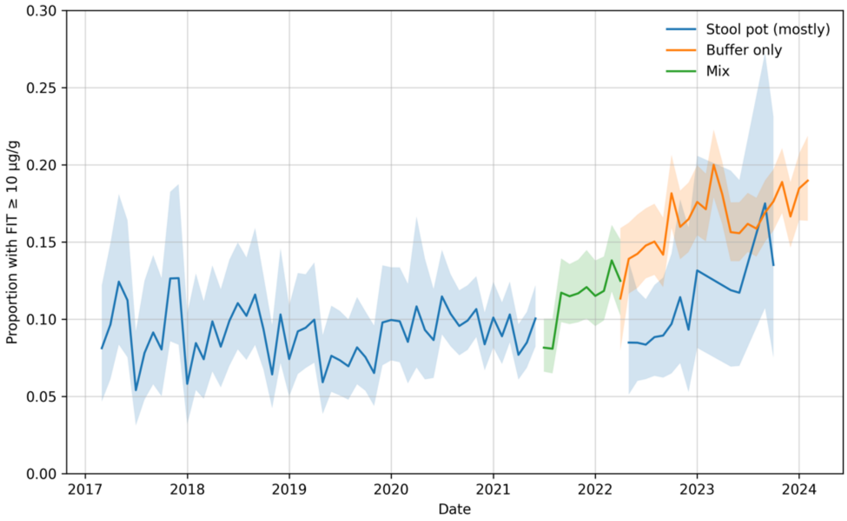

Figure S7E. FIT positivity over time grouped by faecal sampling method. Proportion of patients testing positive for the FIT test at the 10 µg/g threshold each month, grouped by FIT sample collection device. Before July 2021, FIT samples were generally collected into stool pots (blue), and the sample pickers with buffer solution ('buffer devices') were introduced afterwards. Since April 2022, it is known how many tests were returned in buffer devices as comments about the stool pot were available alongside FIT results – FIT positivity among these tests is shown separately as the orange line. Data about buffer devices was not available between July 2021 and April 2022, so FIT positivity for tests in that period is shown in green. Shaded areas show 95% Wilson confidence intervals. The proportion of tests in stool pot gradually dropped to less than 10% since the introduction of the buffer device, and hence the confidence intervals are wide for tests done in stool pots since April 2022. Proportions were only computed for months with at least 10 FIT positive patients.
