# Supplementary material for: External validation of the COLOFIT colorectal cancer risk prediction model in the Oxford-FIT dataset: the importance of population characteristics and clinically relevant evaluation metrics
Source: BMC Med. 2025 Aug 27;23:503. doi: 10.1186/s12916-025-04339-w (PMC12392603; doi:10.1186/s12916-025-04339-w)
Supplement: Supplementary file 8 — Additional File 8: Descriptive statistics for the patient cohort and blood tests over time: Tables S8A– S8B. Tab S8A – Descriptive statistics for the patient cohort over the six time periods. Tab S8B – Summaries of selected blood tests over the six time periods [file 12916_2025_4339_MOESM8_ESM.pdf]

S8. DESCRIPTIVE STATISTICS FOR THE PATIENT COHORT AND BLOOD TESTS OVER TIME

Descriptive statistics for the patient cohort over the six time periods can be found in Table S8A; and summaries of core blood tests in Table S8B.

Table S8A. Descriptive statistics for the patient cohort over the six time periods

	Pre-COVID (2017/01 - 2020/02)		COVID (2020/03 - 2021/04)		Post-COVID (2021/05 - 2021/12)		2022 H1 (2022/01 - 2022/06)		2022 H2 (2022/07 - 2022/12)		2023 H1 (2023/01 - 2023/06)	
	**No CRC**	**CRC**	**No CRC**	**CRC**	**No CRC**	**CRC**	**No CRC**	**CRC**	**No CRC**	**CRC**	**No CRC**	**CRC**
Number of patients	10255	124	8762	128	7373	99	5884	88	7386	104	8229	91
Age												
18-39.9	806 (7.9%)	Not Available	830 (9.5%)	Not Available	741 (10.1%)	Not Available	652 (11.1%)	-	1017 (13.8%)	Not Available	1326 (16.1%)	Not Available
40-49.9	1432 (14.0%)	Not Available	1043 (11.9%)	Not Available	898 (12.2%)	Not Available	780 (13.3%)	Not Available	1039 (14.1%)	Not Available	1147 (13.9%)	Not Available
50-59.9	2804 (27.3%)	22 (17.7%)	1709 (19.5%)	13 (10.2%)	1407 (19.1%)	17 (17.2%)	1133 (19.3%)	Not Available	1386 (18.8%)	Not Available	1523 (18.5%)	15 (16.5%)
60-69.9	1692 (16.5%)	22 (17.7%)	1630 (18.6%)	18 (14.1%)	1297 (17.6%)	Not Available	1028 (17.5%)	17 (19.3%)	1267 (17.2%)	19 (18.3%)	1419 (17.2%)	21 (23.1%)
70-79.9	2004 (19.5%)	32 (25.8%)	1996 (22.8%)	40 (31.2%)	1681 (22.8%)	29 (29.3%)	1315 (22.3%)	31 (35.2%)	1554 (21.0%)	36 (34.6%)	1575 (19.1%)	27 (29.7%)
≥80	1517 (14.8%)	34 (27.4%)	1554 (17.7%)	47 (36.7%)	1349 (18.3%)	29 (29.3%)	976 (16.6%)	22 (25.0%)	1123 (15.2%)	30 (28.8%)	1239 (15.1%)	18 (19.8%)
Median (25th, 75th)	60.7 (51.2, 74.7)	70.5 (58.1, 80.7)	64.9 (52.0, 76.8)	75.0 (66.3, 82.5)	65.1 (51.5, 77.2)	73.2 (59.5, 80.8)	63.7 (50.3, 76.2)	75.0 (61.7, 79.8)	62.2 (48.3, 75.6)	75.1 (64.5, 80.9)	60.8 (46.3, 75.0)	69.9 (57.6, 78.4)
Min, max	18.2, 100.7	31.1, 91.2	18.3, 100.9	31.6, 92.2	18.3, 101.4	26.9, 90.8	18.0, 101.6	40.9, 91.2	18.2, 102.7	38.8, 102.3	18.2, 99.7	35.4, 91.9
Gender												
F or non-binary (<10)	6038 (58.9%)	51 (41.1%)	4989 (56.9%)	60 (46.9%)	4330 (58.7%)	43 (43.4%)	3503 (59.5%)	46 (52.3%)	4346 (58.8%)	49 (47.1%)	4845 (58.9%)	33 (36.3%)
M	4217 (41.1%)	73 (58.9%)	3773 (43.1%)	68 (53.1%)	3043 (41.3%)	56 (56.6%)	2381 (40.5%)	42 (47.7%)	3040 (41.2%)	55 (52.9%)	3384 (41.1%)	58 (63.7%)
Ethnicity												
Asian	232 (2.3%)	Not Available	215 (2.5%)	-	199 (2.7%)	-	150 (2.5%)	-	193 (2.6%)	Not Available	208 (2.5%)	Not Available
Black	76 (0.7%)	Not Available	66 (0.8%)	-	57 (0.8%)	-	48 (0.8%)	-	58 (0.8%)	-	76 (0.9%)	Not Available
Mixed	69 (0.7%)	-	48 (0.5%)	-	42 (0.6%)	Not Available	38 (0.6%)	-	44 (0.6%)	-	67 (0.8%)	-
Not known	132 (1.3%)	-	142 (1.6%)	Not Available	137 (1.9%)	-	139 (2.4%)	Not Available	211 (2.9%)	-	272 (3.3%)	-
Not stated	1799 (17.5%)	Not Available	1610 (18.4%)	Not Available	1439 (19.5%)	Not Available	1189 (20.2%)	Not Available	1565 (21.2%)	Not Available	1803 (21.9%)	Not Available
Other Ethnic Groups	91 (0.9%)	-	78 (0.9%)	Not Available	80 (1.1%)	Not Available	69 (1.2%)	-	74 (1.0%)	Not Available	97 (1.2%)	-
White	7856 (76.6%)	98 (79.0%)	6603 (75.4%)	98 (76.6%)	5419 (73.5%)	78 (78.8%)	4251 (72.2%)	65 (73.9%)	5241 (71.0%)	76 (73.1%)	5706 (69.3%)	63 (69.2%)
IMDD												
Median (25th, 75th)	8.0 (7.0, 10.0)	9.0 (7.0, 10.0)	8.0 (7.0, 10.0)	8.0 (7.0, 10.0)	8.0 (7.0, 9.0)	8.0 (7.0, 9.0)	8.0 (6.0, 10.0)	9.0 (7.0, 10.0)	8.0 (7.0, 10.0)	9.0 (7.0, 9.0)	8.0 (6.0, 10.0)	9.0 (7.0, 10.0)
Min, max	1.0, 10.0	2.0, 10.0	1.0, 10.0	1.0, 10.0	1.0, 10.0	2.0, 10.0	1.0, 10.0	2.0, 10.0	1.0, 10.0	2.0, 10.0	1.0, 10.0	2.0, 10.0
Not known	802 (7.8%)	<10	754 (8.6%)	<10	684 (9.3%)	<10	600 (10.2%)	<10	800 (10.8%)	<10	963 (11.7%)	<10
FIT (μg Hb/g)												
0-1.9	8548 (83.4%)	Not Available	7350 (83.9%)	Not Available	6072 (82.4%)	Not Available	4593 (78.1%)	Not Available	5460 (73.9%)	Not Available	5574 (67.7%)	Not Available
2-9.9	900 (8.8%)	Not Available	669 (7.6%)	Not Available	620 (8.4%)	Not Available	621 (10.6%)	Not Available	940 (12.7%)	Not Available	1352 (16.4%)	Not Available
10-99.9	593 (5.8%)	36 (29.0%)	499 (5.7%)	46 (35.9%)	477 (6.5%)	32 (32.3%)	449 (7.6%)	Not Available	677 (9.2%)	27 (26.0%)	852 (10.4%)	Not Available
≥100	214 (2.1%)	71 (57.3%)	244 (2.8%)	64 (50.0%)	204 (2.8%)	50 (50.5%)	221 (3.8%)	55 (62.5%)	309 (4.2%)	65 (62.5%)	451 (5.5%)	58 (63.7%)
Median (25th, 75th)	0.0 (0.0, 0.0)	170.6 (29.7, 400.0)	0.0 (0.0, 0.0)	107.8 (24.8, 400.0)	0.0 (0.0, 0.0)	124.0 (15.0, 400.0)	0.0 (0.0, 0.0)	217.5 (44.8, 400.0)	0.0 (0.0, 2.0)	237.0 (33.0, 400.0)	0.0 (0.0, 3.0)	210.0 (45.0, 400.0)
Min, max	0.0, 400.0	0.0, 400.0	0.0, 400.0	0.0, 400.0	0.0, 400.0	0.0, 400.0	0.0, 400.0	0.0, 400.0	0.0, 400.0	0.0, 400.0	0.0, 400.0	0.0, 400.0
Symptoms - GP reported												
Abdominal mass	Not Available	-	Not Available	-	Not Available	Not Available	Not Available	Not Available	Not Available	-	Not Available	-
Abdominal pain	1517 (14.8%)	16 (12.9%)	1316 (15.0%)	12 (9.4%)	992 (13.5%)	12 (12.1%)	790 (13.4%)	10 (11.4%)	908 (12.3%)	Not Available	1095 (13.3%)	11 (12.1%)
Anaemia	1609 (15.7%)	29 (23.4%)	1174 (13.4%)	26 (20.3%)	980 (13.3%)	19 (19.2%)	735 (12.5%)	19 (21.6%)	887 (12.0%)	23 (22.1%)	963 (11.7%)	Not Available
Bloating	293 (2.9%)	Not Available	297 (3.4%)	Not Available	273 (3.7%)	Not Available	192 (3.3%)	-	243 (3.3%)	Not Available	278 (3.4%)	Not Available
Blood in stool	802 (7.8%)	15 (12.1%)	843 (9.6%)	22 (17.2%)	658 (8.9%)	15 (15.2%)	589 (10.0%)	15 (17.0%)	952 (12.9%)	19 (18.3%)	1270 (15.4%)	20 (22.0%)
Change in bowel habit	2348 (22.9%)	23 (18.5%)	2106 (24.0%)	31 (24.2%)	1747 (23.7%)	34 (34.3%)	1521 (25.8%)	22 (25.0%)	1938 (26.2%)	28 (26.9%)	1902 (23.1%)	25 (27.5%)
Constipation	334 (3.3%)	Not Available	367 (4.2%)	Not Available	301 (4.1%)	Not Available	195 (3.3%)	Not Available	214 (2.9%)	Not Available	255 (3.1%)	Not Available
Diarrhoea	1239 (12.1%)	11 (8.9%)	1056 (12.1%)	12 (9.4%)	888 (12.0%)	10 (10.1%)	686 (11.7%)	Not Available	756 (10.2%)	Not Available	885 (10.8%)	Not Available
Family history of CRC	118 (1.2%)	Not Available	70 (0.8%)	-	65 (0.9%)	-	62 (1.1%)	-	87 (1.2%)	-	58 (0.7%)	-
Fatigue	114 (1.1%)	-	91 (1.0%)	Not Available	128 (1.7%)	-	104 (1.8%)	Not Available	134 (1.8%)	Not Available	166 (2.0%)	-
Inflammation	149 (1.5%)	-	126 (1.4%)	Not Available	85 (1.2%)	Not Available	68 (1.2%)	-	90 (1.2%)	-	79 (1.0%)	-
Iron deficiency anaemia	548 (5.3%)	Not Available	599 (6.8%)	13 (10.2%)	483 (6.6%)	Not Available	347 (5.9%)	13 (14.8%)	358 (4.8%)	Not Available	442 (5.4%)	Not Available
Low iron	307 (3.0%)	Not Available	259 (3.0%)	-	218 (3.0%)	Not Available	169 (2.9%)	-	184 (2.5%)	Not Available	219 (2.7%)	Not Available
Melaena	162 (1.6%)	Not Available	87 (1.0%)	-	61 (0.8%)	-	57 (1.0%)	-	73 (1.0%)	-	58 (0.7%)	-
Not known	1444 (14.1%)	24 (19.4%)	1150 (13.1%)	17 (13.3%)	992 (13.5%)	11 (11.1%)	853 (14.5%)	Not Available	1159 (15.7%)	14 (13.5%)	1300 (15.8%)	13 (14.3%)
Rectal bleeding	425 (4.1%)	Not Available	517 (5.9%)	14 (10.9%)	433 (5.9%)	Not Available	371 (6.3%)	Not Available	611 (8.3%)	15 (14.4%)	869 (10.6%)	12 (13.2%)
Rectal mass			Not Available	-	Not Available	-	Not Available	-	Not Available	Not Available	Not Available	-
Rectal pain	Not Available	-	67 (0.8%)	Not Available	47 (0.6%)	-	Not Available	Not Available	40 (0.5%)	-	50 (0.6%)	Not Available
Thrombocytosis	146 (1.4%)	Not Available	87 (1.0%)	Not Available	65 (0.9%)	Not Available	68 (1.2%)	Not Available	83 (1.1%)	-	89 (1.1%)	Not Available
Weight loss	640 (6.2%)	Not Available	642 (7.3%)	11 (8.6%)	613 (8.3%)	Not Available	462 (7.9%)	11 (12.5%)	621 (8.4%)	Not Available	601 (7.3%)	Not Available
T stage												
1	-	15 (12.1%)	-	16 (12.5%)	-	Not Available	-	Not Available	-	Not Available	-	10 (11.0%)
2	-	10 (8.1%)	-	12 (9.4%)	-	Not Available	-	Not Available	-	Not Available	-	Not Available
3	-	34 (27.4%)	-	31 (24.2%)	-	23 (23.2%)	-	28 (31.8%)	-	26 (25.0%)	-	20 (22.0%)
4	-	13 (10.5%)	-	16 (12.5%)	-	Not Available	-	16 (18.2%)	-	12 (11.5%)	-	Not Available
Not known	-	52 (41.9%)	-	53 (41.4%)	-	53 (53.5%)	-	33 (37.5%)	-	49 (47.1%)	-	45 (49.5%)

Note. Rows where the result is shown as “Not available” had a count less than 10. If only one count in a data category was less than 10, or all counts less than 10 did not add up to 10, the value with the next highest count was also marked as “Not available”. This is due to data governance requirements.

Table S8B. Summaries of selected blood tests over the six time periods

	Pre-COVID (2017/01 - 2020/02)		COVID (2020/03 - 2021/04)		Post-COVID (2021/05 - 2021/12)		2022 H1 (2022/01 - 2022/06)		2022 H2 (2022/07 - 2022/12)		2023 H1 (2023/01 - 2023/06)	
	**No CRC**	**CRC**	**No CRC**	**CRC**	**No CRC**	**CRC**	**No CRC**	**CRC**	**No CRC**	**CRC**	**No CRC**	**CRC**
Number of patients	10255	124	8762	128	7373	99	5884	88	7386	104	8229	91
Haemoglobin (HGB)												
Median (25th, 75th)	133.0 (121.0, 144.0)	124.0 (110.8, 142.2)	133.0 (121.0, 144.0)	125.0 (106.0, 136.0)	133.0 (121.0, 144.0)	123.0 (108.5, 139.5)	134.0 (123.0, 145.0)	120.0 (102.0, 137.2)	134.0 (123.0, 145.0)	125.0 (101.0, 141.5)	134.0 (123.0, 145.0)	132.0 (116.0, 143.0)
Min, max	50.0, 189.0	69.0, 168.0	52.0, 226.0	55.0, 168.0	54.0, 219.0	53.0, 166.0	53.0, 184.0	56.0, 165.0	57.0, 200.0	67.0, 184.0	54.0, 199.0	79.0, 163.0
Not known	-	-	Not Available	-	-	-	-	-	Not Available	-	-	-
low haemoglobin	3565 (34.8%)	64 (51.6%)	3110 (35.5%)	69 (53.9%)	2521 (34.2%)	55 (55.6%)	1893 (32.2%)	51 (58.0%)	2322 (31.4%)	62 (59.6%)	2566 (31.2%)	34 (37.4%)
normal haemoglobin	8124 (79.2%)	72 (58.1%)	6939 (79.2%)	78 (60.9%)	5940 (80.6%)	60 (60.6%)	4865 (82.7%)	46 (52.3%)	6103 (82.6%)	55 (52.9%)	6855 (83.3%)	70 (76.9%)
Platelets (PLT)												
Median (25th, 75th)	261.0 (220.0, 310.0)	308.0 (246.8, 372.5)	267.0 (223.0, 316.0)	293.5 (244.8, 363.8)	266.0 (224.0, 318.0)	301.0 (242.5, 364.5)	268.0 (225.0, 319.0)	311.5 (249.2, 369.2)	267.0 (225.0, 316.0)	318.5 (243.2, 394.8)	271.0 (227.0, 322.0)	300.0 (238.0, 361.0)
Min, max	9.0, 1235.0	103.0, 920.0	46.0, 894.0	120.0, 654.0	11.0, 855.0	151.0, 722.0	46.0, 1241.0	149.0, 723.0	23.0, 848.0	102.0, 651.0	12.0, 926.0	93.0, 598.0
high platelets	990 (9.7%)	29 (23.4%)	969 (11.1%)	22 (17.2%)	805 (10.9%)	21 (21.2%)	647 (11.0%)	20 (22.7%)	737 (10.0%)	28 (26.9%)	854 (10.4%)	17 (18.7%)
normal platelets	9860 (96.1%)	109 (87.9%)	8385 (95.7%)	117 (91.4%)	7056 (95.7%)	88 (88.9%)	5616 (95.4%)	79 (89.8%)	7090 (96.0%)	90 (86.5%)	7890 (95.9%)	83 (91.2%)
White cellls (WBC)												
Median (25th, 75th)	6.6 (5.5, 8.0)	7.5 (6.3, 9.2)	6.8 (5.6, 8.2)	7.5 (6.3, 9.1)	6.8 (5.6, 8.2)	7.6 (6.1, 9.2)	6.7 (5.6, 8.0)	7.2 (6.0, 8.9)	6.8 (5.6, 8.2)	7.6 (6.5, 9.4)	6.8 (5.6, 8.2)	7.9 (6.7, 9.2)
Min, max	1.7, 237.5	3.8, 17.4	1.6, 173.6	3.7, 15.5	1.9, 151.4	3.8, 20.3	1.3, 195.2	3.6, 14.6	1.6, 116.0	4.4, 15.1	1.7, 82.1	4.2, 24.6
high white cells	1216 (11.9%)	17 (13.7%)	1149 (13.1%)	21 (16.4%)	934 (12.7%)	16 (16.2%)	703 (11.9%)	Not Available	886 (12.0%)	25 (24.0%)	1051 (12.8%)	15 (16.5%)
normal white cells	10036 (97.9%)	117 (94.4%)	8522 (97.3%)	122 (95.3%)	7167 (97.2%)	94 (94.9%)	5756 (97.8%)	84 (95.5%)	7231 (97.9%)	93 (89.4%)	8030 (97.6%)	85 (93.4%)
Mean cell haemoglobin (MCH)												
Median (25th, 75th)	29.9 (28.4, 31.1)	28.7 (26.4, 30.3)	29.9 (28.5, 31.1)	28.4 (25.6, 30.2)	30.3 (28.9, 31.5)	28.6 (25.8, 30.6)	30.4 (28.9, 31.5)	28.8 (25.6, 30.6)	30.3 (29.0, 31.5)	29.0 (25.3, 30.2)	30.2 (28.8, 31.3)	29.0 (27.6, 31.0)
Min, max	13.8, 49.0	15.4, 34.2	15.3, 43.9	18.4, 35.2	15.3, 46.0	12.5, 33.7	14.1, 49.4	17.5, 33.1	15.8, 49.6	16.7, 34.1	14.3, 44.1	18.3, 38.7
Not known	-	-	-	-	-	-	-	-	Not Available	-	-	-
low MCH	1862 (18.2%)	38 (30.6%)	1482 (16.9%)	45 (35.2%)	1138 (15.4%)	40 (40.4%)	858 (14.6%)	33 (37.5%)	958 (13.0%)	41 (39.4%)	1171 (14.2%)	26 (28.6%)
normal MCH	8931 (87.1%)	96 (77.4%)	7809 (89.1%)	95 (74.2%)	6606 (89.6%)	70 (70.7%)	5306 (90.2%)	65 (73.9%)	6739 (91.2%)	75 (72.1%)	7434 (90.3%)	74 (81.3%)
Mean cell volume (MCV)												
Median (25th, 75th)	90.9 (87.4, 94.1)	89.2 (84.6, 93.8)	91.2 (87.6, 94.4)	88.8 (83.4, 92.8)	91.2 (87.6, 94.6)	88.1 (81.2, 92.4)	92.1 (88.3, 95.6)	89.8 (82.2, 92.6)	92.7 (89.0, 96.1)	89.1 (82.8, 93.6)	92.2 (88.5, 95.6)	90.1 (86.6, 94.0)
Min, max	53.1, 134.7	61.8, 102.5	57.4, 127.3	66.0, 105.0	54.9, 129.2	55.0, 104.1	54.4, 132.0	63.5, 102.5	56.6, 137.1	61.5, 104.7	57.9, 130.1	65.3, 121.6
low MCV	771 (7.5%)	24 (19.4%)	595 (6.8%)	26 (20.3%)	492 (6.7%)	21 (21.2%)	394 (6.7%)	17 (19.3%)	378 (5.1%)	22 (21.2%)	436 (5.3%)	11 (12.1%)
normal MCV	9799 (95.6%)	108 (87.1%)	8414 (96.0%)	112 (87.5%)	7063 (95.8%)	84 (84.8%)	5649 (96.0%)	77 (87.5%)	7161 (97.0%)	89 (85.6%)	7961 (96.7%)	87 (95.6%)

Note. Normal, high, and low values for these bloods were defined as in Withrow et al[4]. Low HGB: < 130 g/L for males, < 120 g/L for females. High PLT: > 400 * 10^9/L. High WBC: > 11 * 10^9/L. Low MCH: < 27.4 pg/cell. Low MCV: < 80 fl. Rows where the result is shown as “Not available” had a count less than 10. Percentages of low/normal/high results do not add to 100, because these were defined as the presence of at least one low/normal/high result within a [-365, 14] day period around the first FIT test result.
