# Supplementary material for: External validation of the COLOFIT colorectal cancer risk prediction model in the Oxford-FIT dataset: the importance of population characteristics and clinically relevant evaluation metrics
Source: BMC Med. 2025 Aug 27;23:503. doi: 10.1186/s12916-025-04339-w (PMC12392603; doi:10.1186/s12916-025-04339-w)
Supplement: Supplementary file 9 — Additional File 9: Reduction in referrals with 180-day and 365-day follow-ups: Tables S9A–S9B, Figure S9. Tab S9A – Cancers detected with 180-day and 365-day follow-ups. Tab S9B – Reduction in the number of referrals for the COLOFIT model relative to the FIT test with 180-day and 365-day follow-ups. Fig S9 – Reduction in the number of positive tests for the COLOFIT model relative to the FIT test with 180-day and 365-day follow-ups over time [file 12916_2025_4339_MOESM9_ESM.pdf]

S9. REDUCTION IN REFERRALS WITH 180-DAY AND 365-DAY FOLLOW-UPS

As a sensitivity analysis, we used a 365-day (rather than 180-day) follow-up to detect cancers. We split data into six time periods as in the main analysis, but we only used data for the periods where both follow-ups were available for all patients, to explore changes in model performance that can be attributed to follow-up length. Data from the first half of 2023 was not included, as four out of six months of the 2023 data did not have 365 days of follow up. Time periods where both follow-ups were available included the pre-COVID, COVID, post-COVID, 2022 H1 (first half of 2022) and 2022 H2 (second half of 2022) periods.

Using a 365-day follow-up would have led to the detection of 6-15 extra cancers depending on the time period (Table S9A). Therefore, a small number of patients who would have been considered non-cancer cases with 180-day follow-up would have been classified as cancers cases with 365-day follow-up.

We again estimated a threshold for the model that captures the same number of cancers as FIT ≥ 10 µg/g, and computed reduction in referrals for the model compared to the FIT test at this threshold. The threshold was estimated in three different ways: on external Nottingham data, on local Oxford data from the current time period, and on local Oxford data from the previous time period. When using externally estimated threshold, the reduction in referrals was the same with 180-day and 365-day follow-ups in all time periods ('external' in Table S9B). When estimating the threshold locally on each of the current time periods, the reduction in referrals was the same with 180-day and 365-day follow-ups during the pre-COVID, COVID, post-COVID, and 2022 H2 periods; reduction in referrals was somewhat different in the first half of 2022 (-23.23% with 180-day follow-up, -17.22% with 365-day follow-up), although the effect was of similar magnitude and in the same direction ('local-current' in Table S9B, Figure S9). Finally, when estimating the threshold locally on the previous subset of Oxford data, the estimated reduction in referrals was the same with 180-day and 365-day follow-ups during the COVID, post-COVID, and 2022 H1 periods; and somewhat different during 2022 H2 (-28.57% reduction with 180-day follow-up, -21.89% reduction with 365-day follow-up). The effect was again in the same direction but somewhat stronger with 180-day follow-up.

This shows that estimated reduction in referrals was usually not impacted by including the additional cancers that would have been detected with 365-day follow-up. It was only affected in one of the five time periods when model threshold was estimated locally on the data from the same time period, in which case the estimated reduction was smaller but in the same direction and of similar magnitude. It was also affected in one of the four time periods when estimating the threshold on the previous subset of the Oxford data, in which case the estimated reduction was again in the same direction and of not too different magnitude (the estimate was about 1/4 times smaller).

One may also wonder: what does it mean when the estimated reduction in referrals is not affected by follow-up length in a particular time period? Recall that a threshold was computed for the model that captures the same number of cancers as the FIT test, and with 365-day follow-up a small number of FIT positive patients may turn out to be previously undetected cases of cancer. If the reduction in referrals is not affected by follow-up length, it means that the small number of additional FIT positive cancers that are detected with 365-day follow-up are still detected by the same model threshold that was estimated using 180-day follow-up.

Table S9A. Cancers detected with 180-day and 365-day follow-ups

Time period	Num FIT tests	Num cancers (180-day follow-up)	Prevalence of cancer (180-day follow-up)	Num cancers (365-day follow-up)	Prevalence of cancer (%) (365-day follow-up)	Additional cancers detected
Pre-COVID (2017/01 – 2020/02)	10379	124	1.19	133	1.28	9
COVID (2020/03 - 2021/04)	8890	128	1.44	143	1.61	15
Post-COVID (2021/05 - 2021/12)	7472	99	1.32	106	1.42	7
2022 H1 (2022/01 - 2022/06)	5972	88	1.47	94	1.57	6
2022 H2 (2022/07 - 2022/12)	7490	104	1.39	114	1.52	10
2023 H1 (2023/01 – 2023/06)	8320	91	1.09	Not yet known	Not yet known	Not yet known

Note. Prevalence of cancer refers to colorectal cancer. 2022 H1 means "the first half of 2022".

Table S9B. Reduction in the number of referrals for the COLOFIT model relative to the FIT test with 180-day and 365-day follow-ups

Period	Model threshold dataset*	Reduction in referrals (180-day follow-up)	Reduction in referrals (365-day follow-up)
Pre-COVID (2017/01 - 2020/02)	external	-1.2 (-3.78, 1.61)	-1.2 (-3.77, 1.62)
Pre-COVID (2017/01 - 2020/02)	local-current	-1.75 (-4.35, 0.99)	-1.75 (-4.33, 1.04)
COVID (2020/03 - 2021/04)	external	-0.94 (-3.85, 1.97)	-0.94 (-3.86, 1.98)
COVID (2020/03 - 2021/04)	local-current	-8.68 (-11.29, -5.95)	-8.68 (-11.34, -6.19)
COVID (2020/03 - 2021/04)	local-previous	-1.99 (-4.9, 0.81)	-1.99 (-5.13, 0.86)
Post-COVID (2021/05 - 2021/12)	external	-1.57 (-4.52, 1.16)	-1.57 (-4.26, 1.18)
Post-COVID (2021/05 - 2021/12)	local-current	-3.8 (-6.69, -0.93)	-3.8 (-6.41, -0.95)
Post-COVID (2021/05 - 2021/12)	local-previous	-9.04 (-11.7, -6.22)	-9.04 (-11.6, -6.39)
2022 H1 (2022/01 - 2022/06)	external	-3.47 (-6.26, -0.71)	-3.47 (-6.24, -0.68)
2022 H1 (2022/01 - 2022/06)	local-current	-23.23 (-26.38, -20.43)	-17.22 (-19.86, -14.34)
2022 H1 (2022/01 - 2022/06)	local-previous	-5.34 (-8.21, -2.66)	-5.34 (-7.97, -2.55)
2022 H2 (2022/07 - 2022/12)	external	-9.37 (-11.88, -7.05)	-9.37 (-11.72, -7.05)
2022 H2 (2022/07 - 2022/12)	local-current	-17.35 (-19.95, -15.06)	-17.35 (-19.94, -15.02)
2022 H2 (2022/07 - 2022/12)	local-previous	-28.57 (-31.46, -25.89)	-21.89 (-24.64, -19.47)

Note. A threshold was chosen for the risk scores of the COLOFIT model such that the model captured the same number of cancers as the FIT test at threshold 10 μg/g. The number of patients who tested positive at this threshold was compared to the number of patients testing positive for FIT to obtain reduction in referrals. Values below zero indicate reduction in referrals: for example, -20 means 20% less patients test positive compared to FIT. Values above zero indicate that the model led to more referrals than the FIT test to capture the same number of cancers.

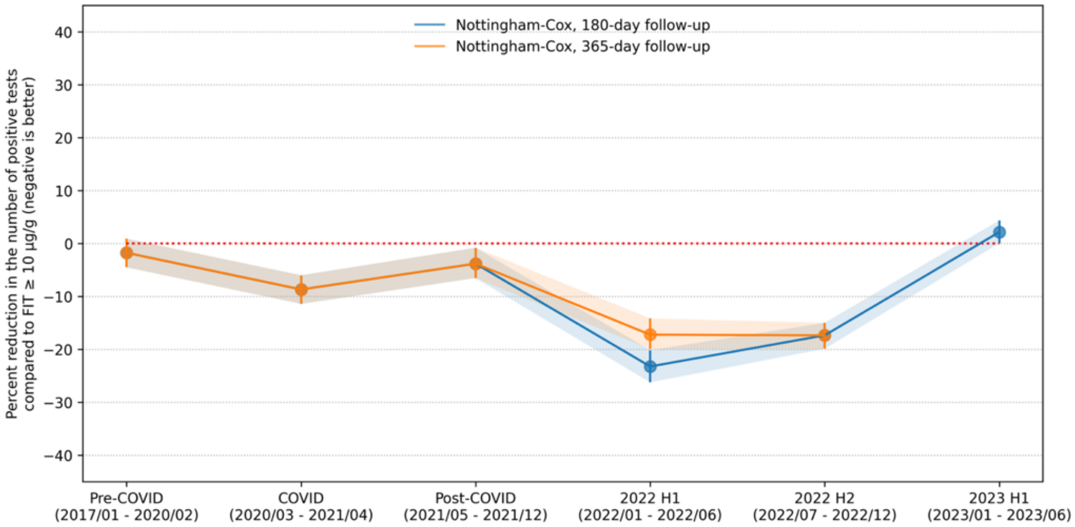

Figure S9. Reduction in the number of positive tests (referrals) for the COLOFIT model relative to the FIT test with 180-day and 365-day follow-ups over time. A threshold was chosen for the risk scores of the COLOFIT model such that the model captured the same number of cancers as the FIT test at threshold 10 µg/g. The number of patients who tested positive at this threshold was compared to the number of patients testing positive for FIT to obtain reduction in referrals. Values below zero indicate reduction in referrals: for example, -20 means 20% less patients test positive compared to FIT. Values above zero indicate that the model led to more referrals than the FIT test to capture the same number of cancers.
