# Supplementary material for: External validation of the COLOFIT colorectal cancer risk prediction model in the Oxford-FIT dataset: the importance of population characteristics and clinically relevant evaluation metrics
Source: BMC Med. 2025 Aug 27;23:503. doi: 10.1186/s12916-025-04339-w (PMC12392603; doi:10.1186/s12916-025-04339-w)
Supplement: Supplementary file 10 — Additional File 10: Calibration curves for recalibrated COLOFIT models: Figure S10.Figure S10 — Calibration curves for recalibrated COLOFIT models [file 12916_2025_4339_MOESM10_ESM.pdf]

S10. CALIBRATION CURVES FOR RECALIBRATED COLOFIT MODELS

The COLOFIT models were recalibrated using three methods (constants multiplication of FIT values, quantile transformation of FIT values, and logistic recalibration – see Methods in main text). Calibration curves for the recalibrated models across the six time periods in OUH-FIT data (and in all data) are given in Figure S10. Quantile transformation for sufficient for approximately recalibrating the models in the clinically most meaningful range of risk (< 6%), indicating that model miscalibration was driven by differences in the FIT test values between Oxford and Nottingham.

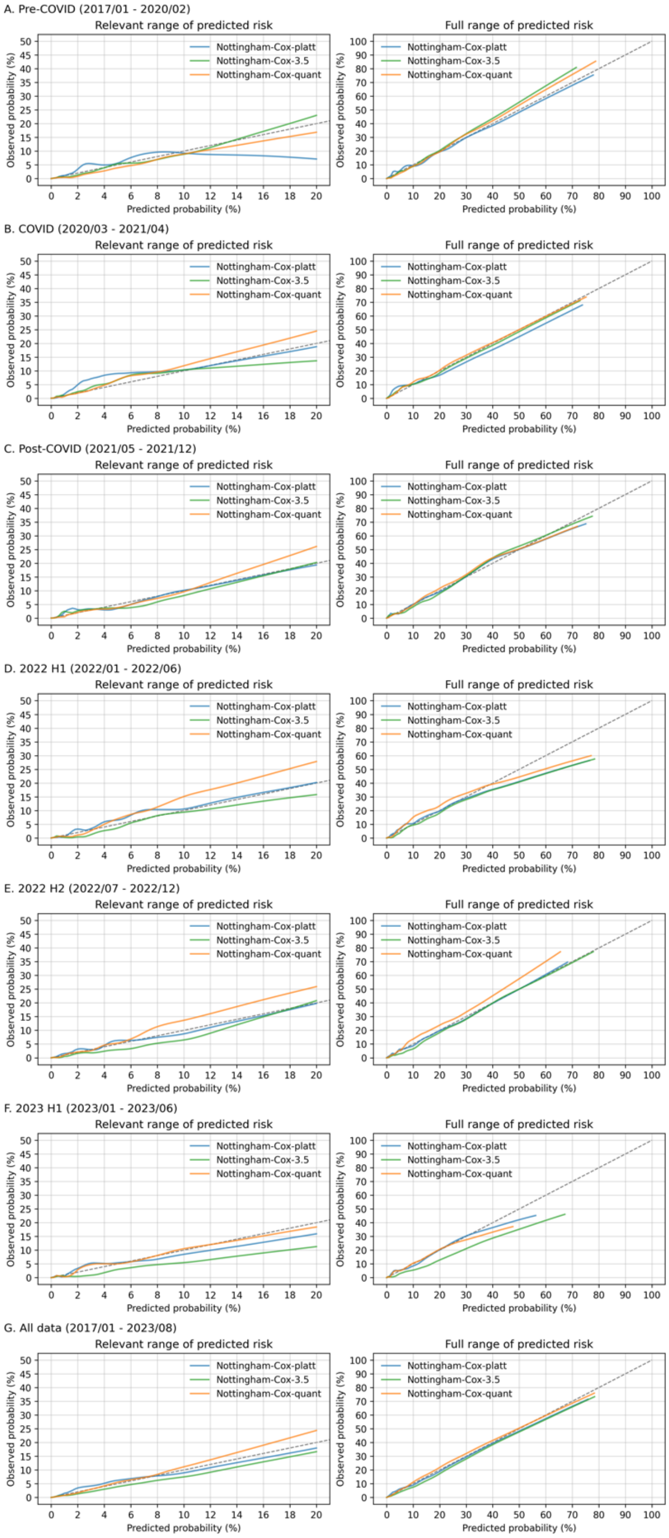

Figure S10. Calibration curves for recalibrated COLOFIT models. Three methods of recalibration were used: logistic recalibration (-platt), multiplying FIT values by a constant of 3.5 (-3.5) and quantile transformation of Oxford FIT values to Nottingham FIT values before entering them to the model (-quant). Time periods cover the pre-COVID, COVID and post-COVID periods, the first and second halves of 2022 and the first half of 2023. The curves were obtained by applying LOWESS-smoothing on the data of predicted probabilities (x) and indicator for the presence of cancer (y).
