# Supplementary material for: External validation of the COLOFIT colorectal cancer risk prediction model in the Oxford-FIT dataset: the importance of population characteristics and clinically relevant evaluation metrics
Source: BMC Med. 2025 Aug 27;23:503. doi: 10.1186/s12916-025-04339-w (PMC12392603; doi:10.1186/s12916-025-04339-w)
Supplement: Supplementary file 11 — Additional File 11: Effect of the FIT analytic device on COLOFIT calibration [file 12916_2025_4339_MOESM11_ESM.pdf]

S11. EFFECT OF THE FIT ANALYTIC DEVICE ON COLOFIT CALIBRATION

Miscalibration was partly caused by differences in FIT analytic device between Nottingham and Oxford. Nottingham FIT values ranged up to 68,900 µg/g due to the use of the OC sensor with larger analytic range, whereas Oxford FIT values were capped at 400 µg/g due to the limits of the HM-JACKarc sensor.

In Nottingham, 25% of cancer patients had a FIT value greater than 1146 µg/g. If these individuals had median Oxford cancer patient values for all other variables (gender Male, age 74, platelet count 305 * 10^9/L, and mean cell volume 89.1 pg/cell), then their COLOFIT risk score would be 36%. However, if their FIT value was capped at 400 µg/g, the risk score would drop to 26%, a significant decrease. Similarly, 10% of Nottingham cancer patients had a FIT value greater than 4640 µg/g – their COLOFIT risk score when other predictors are set at Oxford cancer patient median would be 46%, and it would again drop to 26% if FIT was capped at 400 µg/g. Finally, 5% of Nottingham cancer patients had FIT greater than 8303 µg/g, which would result in COLOFIT risk score of 49% if other predictors are set at median, a significant increase from 26%.

This behaviour could be avoided if FIT values would be capped at 400 during model derivation.
