# Supplementary material for: External validation of the COLOFIT colorectal cancer risk prediction model in the Oxford-FIT dataset: the importance of population characteristics and clinically relevant evaluation metrics
Source: BMC Med. 2025 Aug 27;23:503. doi: 10.1186/s12916-025-04339-w (PMC12392603; doi:10.1186/s12916-025-04339-w)
Supplement: Supplementary file 12 — Additional File 12: Decision curves for recalibrated COLOFIT models: Figure S12. Fig S12 —Decision curves [file 12916_2025_4339_MOESM12_ESM.pdf]

S12. DECISION CURVES FOR RECALIBRATED COLOFIT MODELS

Figure S12 displays decision curves (net benefit curves) for the recalibrated COLOFIT model ('Nottingham-Cox-quant'), for the FIT-only spline model derived on OUH-FIT data, and for the FIT test at threshold ≥ 10 µg/g. It is important to compute net benefit statistics for calibrated models, as otherwise the levels of predicted risk (the *x*-axis) may not be comparable between models and cannot be interpreted as probabilities of colorectal cancer. The COLOFIT model was recalibrated using quantile transformation of FIT values as it appeared to work well for risk levels near FIT 10 µg/g (see Section S7 for its calibration curves). It is also important to include a FIT-only model, as it covers predicted risks corresponding to all FIT values, just like the COLOFIT model can be used at multiple levels of predicted risk. Otherwise, COLOFIT would be used at multiple thresholds and FIT at one threshold, and it would not be clear whether benefits provided by the model would remain if FIT was simply used at a different threshold.

Based on the FIT-only spline model, probability of CRC corresponding to FIT 10 µg/g was 1.7% on all data, 1.7% pre-COVID, 3.0% during COVID, 2.5% post-COVID, 1.6% in 2022 H1, 1.5% in 2022 H2, and 1% in 2023 H1. Net benefit curves did not show clear differences between the model and FIT at these levels of predicted risk (in corresponding time periods), even though there were significant reductions in referrals observed in 2022 H1 and 2022 H2. What could be the cause of this discrepancy? Firstly, net benefit and reduction in referrals are different quantities that are not expected to match. Net benefit is proportional to the number of true positives minus the odds of cancer times false positives (a weighted difference of true and false positives), whereas the number of referrals is the total number of positives. Therefore, the difference in net benefit between model and FIT, and reduction in referrals for the model relative to FIT, will not yield the same result. Secondly, the models cannot be perfectly calibrated, so the true net benefit at a particular risk score threshold may be different from the observed net benefit due to calibration error. Furthermore, net benefit assumes that the cost of false positives is higher when the odds of cancer are higher, which is likely an oversimplification (see Supplementary Materials S9). Health system managers and clinicians would arguably be more interested in reducing the total number of patients that are referred to colonoscopy while capturing the same number of cancers as FIT—a quantity represented by reduction in referrals—whereas the difference in true and false positives given by net benefit does not have the same clinically relevant interpretation.

Note that there were also no clear differences in net benefit between COLOFIT and FIT in COLOFIT internal validation data, at the 0.64% risk threshold corresponding to FIT 10 µg/g, even though the model had a large 20.2% reduction in referrals (Figure 1 in Crooks et al [20]).

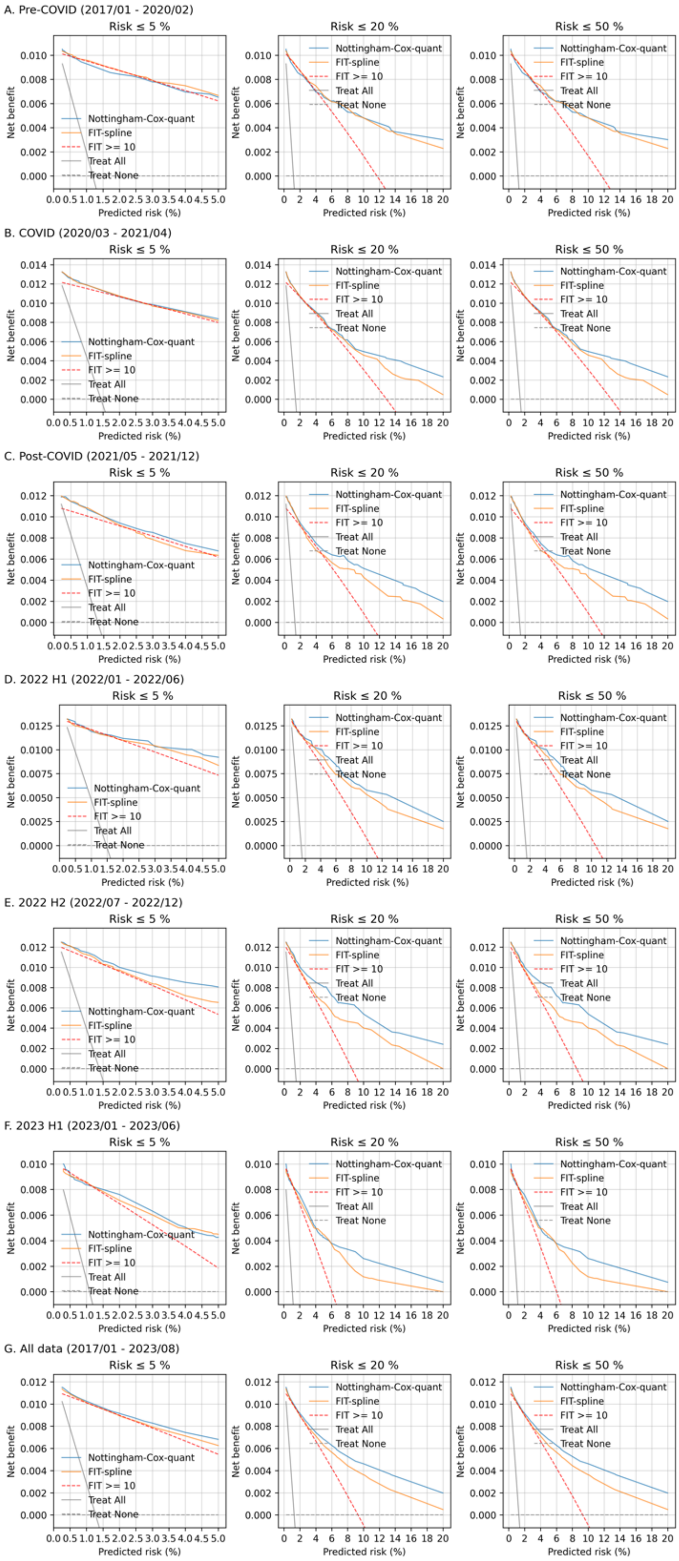

Figure S12. Decision curves. Decision curves, also known as net benefit curves, are shown for the COLOFIT model, the Oxford FIT-spline model, and for the FIT test at threshold ≥ 10 µg/g. The FIT-spline model predicts the risk of colorectal cancer from FIT values in Oxford data and has knots at values 10 and 100; it represents the performance of FIT test over all possible levels of predicted risk (as the FIT test can be applied at multiple thresholds). The COLOFIT model (Nottingham-Cox) was recalibrated using quantile transformation of FIT values before computing the decision curves, as the original Cox model was not calibrated in Oxford data.
