# Supplementary material for: External validation of the COLOFIT colorectal cancer risk prediction model in the Oxford-FIT dataset: the importance of population characteristics and clinically relevant evaluation metrics
Source: BMC Med. 2025 Aug 27;23:503. doi: 10.1186/s12916-025-04339-w (PMC12392603; doi:10.1186/s12916-025-04339-w)
Supplement: Supplementary file 13 — Additional File 13: Diagnostic metrics at selected levels of predicted risk: Table S13. Tab S13 – Performance metrics computed at selected levels of predicted risk for the recalibrated COLOFIT model and the Oxford FIT-spline model [file 12916_2025_4339_MOESM13_ESM.pdf]

S13. DIAGNOSTIC METRICS AT SELECTED LEVELS OF PREDICTED RISK

Common diagnostic metrics of model performance at various levels of predicted risks are reported for the recalibrated COLOFIT model (‘Nottingham-Cox-quant’) and for a FIT-only spline model in Table S13. It is important to report these metrics for *calibrated* models, as in that case predicted risks approximately correspond to probabilities of colorectal cancer. To compare the COLOFIT model to the FIT test, we also report these metrics for a FIT-only model that was fitted to OUH-FIT data and associates each FIT value with a probability of colorectal cancer. Confidence intervals are bootstrap percentile intervals. Based on the FIT-only spline model, probability of CRC corresponding to FIT 10 μg/g was 1.7% on all data, 1.7% pre-COVID, 3.0% during COVID, 2.5% post-COVID, 1.6% in 2022 H1, 1.5% in 2022 H2, and 1% in 2023 H1.

Table S13. Performance metrics computed at selected levels of predicted risk for the recalibrated COLOFIT model (Nottingham-Cox-quant) and the Oxford FIT-spline model

Predicted risk (%)	Num patients	Num cancers	Model	Positive tests per 1000 tests	Negative tests per 1000 tests	Detected cancers per 1000 tests	Missed cancers per 1000 tests	Sensitivity (%)	Specificity (%)	PPV (%)	NPV (%)
**Pre-COVID (2017/01 - 2020/02)**											
0.5	10379	124	FIT-spline	155.99 (148.67, 162.54)	844.01 (837.46, 851.33)	10.79 (8.86, 12.72)	1.16 (0.58, 1.83)	90.32 (85.04, 95.32)	85.3 (84.63, 86.02)	6.92 (5.69, 8.15)	99.86 (99.78, 99.93)
0.5	10379	124	Nottingham-Cox-quant	198.48 (190.77, 203.68)	801.52 (796.32, 809.23)	10.89 (9.06, 12.81)	1.06 (0.48, 1.73)	91.13 (86.03, 95.94)	81.01 (80.49, 81.77)	5.49 (4.59, 6.53)	99.87 (99.78, 99.94)
0.6	10379	124	FIT-spline	141.15 (134.21, 147.32)	858.85 (852.68, 865.79)	10.69 (8.86, 12.62)	1.25 (0.58, 2.02)	89.52 (83.78, 94.78)	86.8 (86.21, 87.48)	7.58 (6.26, 8.96)	99.85 (99.77, 99.93)
0.6	10379	124	Nottingham-Cox-quant	190.48 (183.92, 194.05)	809.52 (805.95, 816.08)	10.89 (9.06, 12.81)	1.06 (0.48, 1.73)	91.13 (86.03, 95.94)	81.82 (81.46, 82.52)	5.72 (4.76, 6.81)	99.87 (99.79, 99.94)
1	10379	124	FIT-spline	110.99 (105.02, 116.97)	889.01 (883.03, 894.98)	10.6 (8.77, 12.53)	1.35 (0.67, 2.12)	88.71 (82.76, 93.91)	89.84 (89.25, 90.44)	9.55 (7.95, 11.28)	99.85 (99.76, 99.92)
1	10379	124	Nottingham-Cox-quant	159.65 (156.08, 163.21)	840.35 (836.79, 843.92)	10.79 (8.86, 12.72)	1.16 (0.58, 1.83)	90.32 (85.04, 95.2)	84.93 (84.56, 85.31)	6.76 (5.59, 7.96)	99.86 (99.78, 99.93)
2	10379	124	FIT-spline	82.57 (77.37, 87.87)	917.43 (912.13, 922.63)	10.21 (8.38, 12.14)	1.73 (0.96, 2.6)	85.48 (78.9, 91.53)	92.68 (92.17, 93.21)	12.37 (10.29, 14.58)	99.81 (99.72, 99.89)
2	10379	124	Nottingham-Cox-quant	117.26 (114.75, 120.44)	882.74 (879.56, 885.25)	10.6 (8.77, 12.53)	1.35 (0.67, 2.12)	88.71 (82.81, 93.97)	89.21 (88.87, 89.51)	9.04 (7.43, 10.64)	99.85 (99.76, 99.92)
3	10379	124	FIT-spline	68.31 (63.49, 72.94)	931.69 (927.06, 936.51)	9.73 (8.0, 11.66)	2.22 (1.35, 3.08)	81.45 (74.36, 88.38)	94.07 (93.62, 94.54)	14.25 (11.93, 16.8)	99.76 (99.67, 99.86)
3	10379	124	Nottingham-Cox-quant	91.82 (89.41, 94.81)	908.18 (905.19, 910.59)	10.31 (8.58, 12.24)	1.64 (0.87, 2.41)	86.29 (80.7, 92.74)	91.75 (91.44, 92.05)	11.23 (9.33, 13.37)	99.82 (99.73, 99.9)
4	10379	124	FIT-spline	59.06 (54.63, 63.3)	940.94 (936.7, 945.37)	9.54 (7.8, 11.47)	2.41 (1.45, 3.37)	79.84 (72.65, 86.89)	94.99 (94.56, 95.4)	16.15 (13.41, 19.06)	99.74 (99.64, 99.85)
4	10379	124	Nottingham-Cox-quant	76.31 (73.71, 79.1)	923.69 (920.9, 926.29)	9.73 (8.0, 11.56)	2.22 (1.35, 3.18)	81.45 (74.82, 87.93)	93.26 (92.94, 93.58)	12.75 (10.61, 15.25)	99.76 (99.66, 99.85)
5	10379	124	FIT-spline	50.68 (46.54, 54.73)	949.32 (945.27, 953.46)	8.86 (7.23, 10.79)	3.08 (2.02, 4.24)	74.19 (66.35, 81.99)	95.77 (95.37, 96.16)	17.49 (14.45, 21.03)	99.68 (99.55, 99.79)
5	10379	124	Nottingham-Cox-quant	64.94 (62.14, 67.44)	935.06 (932.56, 937.86)	9.44 (7.8, 11.18)	2.51 (1.64, 3.56)	79.03 (71.54, 85.84)	94.38 (94.12, 94.7)	14.54 (11.97, 17.35)	99.73 (99.62, 99.83)
6	10379	124	FIT-spline	45.09 (41.23, 49.14)	954.91 (950.86, 958.77)	8.48 (6.84, 10.31)	3.47 (2.41, 4.62)	70.97 (62.94, 78.82)	96.29 (95.92, 96.66)	18.8 (15.45, 22.76)	99.64 (99.51, 99.75)
6	10379	124	Nottingham-Cox-quant	55.3 (52.31, 57.13)	944.7 (942.87, 947.69)	9.15 (7.42, 10.89)	2.79 (1.83, 4.05)	76.61 (68.35, 83.78)	95.33 (95.11, 95.67)	16.55 (13.61, 20.0)	99.7 (99.57, 99.81)
7	10379	124	FIT-spline	41.53 (37.67, 45.28)	958.47 (954.72, 962.33)	8.48 (6.84, 10.31)	3.47 (2.41, 4.62)	70.97 (62.94, 78.82)	96.66 (96.3, 97.02)	20.42 (16.86, 24.76)	99.64 (99.52, 99.75)
7	10379	124	Nottingham-Cox-quant	47.11 (44.99, 49.52)	952.89 (950.48, 955.01)	8.77 (7.13, 10.5)	3.18 (2.22, 4.34)	73.39 (65.42, 80.32)	96.12 (95.83, 96.34)	18.61 (15.08, 22.09)	99.67 (99.54, 99.77)
8	10379	124	FIT-spline	37.67 (34.11, 41.24)	962.33 (958.76, 965.89)	8.19 (6.65, 9.93)	3.76 (2.6, 4.91)	68.55 (60.68, 76.75)	97.02 (96.68, 97.35)	21.74 (17.93, 26.28)	99.61 (99.49, 99.73)
8	10379	124	Nottingham-Cox-quant	42.68 (40.08, 44.71)	957.32 (955.29, 959.92)	8.29 (6.84, 10.02)	3.66 (2.6, 4.82)	69.35 (61.61, 77.05)	96.52 (96.28, 96.79)	19.41 (15.95, 23.45)	99.62 (99.5, 99.73)
9	10379	124	FIT-spline	34.88 (31.31, 38.44)	965.12 (961.56, 968.69)	7.71 (6.17, 9.44)	4.24 (3.08, 5.4)	64.52 (56.16, 72.97)	97.25 (96.91, 97.58)	22.1 (18.16, 26.72)	99.56 (99.44, 99.68)
9	10379	124	Nottingham-Cox-quant	38.44 (36.23, 40.57)	961.56 (959.43, 963.77)	8.19 (6.55, 9.83)	3.76 (2.79, 5.01)	68.55 (60.34, 75.57)	96.94 (96.7, 97.17)	21.3 (17.07, 25.38)	99.61 (99.48, 99.71)
10	10379	124	FIT-spline	32.76 (29.29, 36.13)	967.24 (963.87, 970.71)	7.61 (6.16, 9.35)	4.34 (3.08, 5.59)	63.71 (55.93, 72.28)	97.45 (97.14, 97.78)	23.24 (18.99, 27.88)	99.55 (99.42, 99.68)
10	10379	124	Nottingham-Cox-quant	34.78 (32.47, 36.9)	965.22 (963.1, 967.53)	7.8 (6.26, 9.44)	4.14 (2.99, 5.4)	65.32 (57.27, 72.8)	97.27 (97.03, 97.51)	22.44 (18.1, 27.08)	99.57 (99.44, 99.69)
**COVID (2020/03 - 2021/04)**											
0.5	8890	128	FIT-spline	172.33 (164.9, 180.09)	827.67 (819.91, 835.1)	13.5 (11.25, 15.86)	0.9 (0.34, 1.57)	93.75 (89.29, 97.66)	83.88 (83.11, 84.64)	7.83 (6.51, 9.25)	99.89 (99.81, 99.96)
0.5	8890	128	Nottingham-Cox-quant	192.91 (186.95, 201.58)	807.09 (798.42, 813.05)	13.5 (11.25, 15.97)	0.9 (0.22, 1.46)	93.75 (89.81, 98.33)	81.8 (80.96, 82.35)	7.0 (5.79, 8.24)	99.89 (99.82, 99.97)
0.6	8890	128	FIT-spline	161.3 (153.77, 168.84)	838.7 (831.16, 846.23)	13.27 (10.91, 15.64)	1.12 (0.45, 1.91)	92.19 (87.16, 96.67)	84.98 (84.27, 85.75)	8.23 (6.76, 9.68)	99.87 (99.77, 99.95)
0.6	8890	128	Nottingham-Cox-quant	183.24 (177.16, 190.67)	816.76 (809.33, 822.84)	13.5 (11.25, 15.97)	0.9 (0.22, 1.46)	93.75 (89.81, 98.33)	82.78 (82.04, 83.37)	7.37 (6.14, 8.7)	99.89 (99.82, 99.97)
1	8890	128	FIT-spline	138.58 (131.83, 145.22)	861.42 (854.78, 868.17)	13.16 (10.91, 15.52)	1.24 (0.56, 2.02)	91.41 (86.15, 96.15)	87.27 (86.61, 87.97)	9.5 (7.79, 11.18)	99.86 (99.76, 99.93)
1	8890	128	Nottingham-Cox-quant	159.28 (156.13, 164.57)	840.72 (835.43, 843.87)	13.39 (11.25, 15.75)	1.01 (0.34, 1.69)	92.97 (88.81, 97.58)	85.2 (84.63, 85.55)	8.4 (6.97, 9.83)	99.88 (99.8, 99.96)
2	8890	128	FIT-spline	109.0 (102.81, 115.19)	891.0 (884.81, 897.19)	12.71 (10.57, 14.96)	1.69 (0.9, 2.7)	88.28 (82.54, 93.6)	90.23 (89.62, 90.81)	11.66 (9.68, 13.69)	99.81 (99.7, 99.9)
2	8890	128	Nottingham-Cox-quant	118.56 (114.85, 121.71)	881.44 (878.29, 885.15)	12.82 (10.57, 15.07)	1.57 (0.79, 2.47)	89.06 (83.18, 93.89)	89.27 (88.9, 89.7)	10.82 (8.94, 12.74)	99.82 (99.72, 99.91)
3	8890	128	FIT-spline	95.28 (89.43, 101.35)	904.72 (898.65, 910.57)	12.26 (10.24, 14.51)	2.14 (1.12, 3.15)	85.16 (78.57, 91.18)	91.58 (91.04, 92.14)	12.87 (10.69, 15.11)	99.76 (99.65, 99.87)
3	8890	128	Nottingham-Cox-quant	93.81 (90.77, 97.3)	906.19 (902.7, 909.23)	12.37 (10.12, 14.51)	2.02 (1.24, 3.15)	85.94 (78.57, 91.24)	91.74 (91.36, 92.05)	13.19 (10.83, 15.44)	99.78 (99.65, 99.86)
4	8890	128	FIT-spline	86.5 (80.76, 92.01)	913.5 (907.99, 919.24)	12.15 (10.01, 14.4)	2.25 (1.24, 3.37)	84.38 (77.78, 90.27)	92.46 (91.92, 92.99)	14.04 (11.71, 16.42)	99.75 (99.63, 99.86)
4	8890	128	Nottingham-Cox-quant	76.49 (73.45, 79.64)	923.51 (920.36, 926.55)	11.81 (9.56, 13.95)	2.59 (1.69, 3.82)	82.03 (74.14, 87.77)	93.44 (93.08, 93.79)	15.44 (12.5, 18.26)	99.72 (99.58, 99.82)
5	8890	128	FIT-spline	78.63 (73.34, 84.03)	921.37 (915.97, 926.66)	11.7 (9.56, 13.95)	2.7 (1.69, 3.94)	81.25 (74.4, 88.0)	93.21 (92.72, 93.73)	14.88 (12.3, 17.46)	99.71 (99.57, 99.82)
5	8890	128	Nottingham-Cox-quant	66.03 (62.88, 69.18)	933.97 (930.82, 937.12)	11.25 (9.22, 13.39)	3.15 (2.02, 4.39)	78.12 (70.77, 85.35)	94.44 (94.11, 94.76)	17.04 (13.87, 20.24)	99.66 (99.53, 99.78)
6	8890	128	FIT-spline	72.44 (67.49, 77.51)	927.56 (922.49, 932.51)	11.14 (9.11, 13.28)	3.26 (2.02, 4.5)	77.34 (70.0, 84.48)	93.78 (93.3, 94.27)	15.37 (12.7, 18.21)	99.65 (99.52, 99.78)
6	8890	128	Nottingham-Cox-quant	55.91 (53.66, 59.62)	944.09 (940.38, 946.34)	10.24 (8.32, 12.37)	4.16 (2.59, 5.51)	71.09 (63.91, 80.65)	95.37 (94.98, 95.64)	18.31 (14.85, 21.95)	99.56 (99.42, 99.73)
7	8890	128	FIT-spline	68.84 (63.67, 73.9)	931.16 (926.1, 936.33)	10.91 (8.89, 13.05)	3.49 (2.25, 4.84)	75.78 (68.03, 83.21)	94.12 (93.66, 94.6)	15.85 (13.03, 18.78)	99.63 (99.48, 99.76)
7	8890	128	Nottingham-Cox-quant	49.94 (46.46, 52.53)	950.06 (947.47, 953.54)	9.79 (7.76, 11.47)	4.61 (3.26, 6.41)	67.97 (58.86, 75.94)	95.93 (95.62, 96.29)	19.59 (15.64, 23.25)	99.51 (99.33, 99.66)
8	8890	128	FIT-spline	65.35 (60.52, 70.19)	934.65 (929.81, 939.48)	10.57 (8.55, 12.71)	3.82 (2.47, 5.06)	73.44 (65.65, 81.16)	94.44 (93.98, 94.9)	16.18 (13.32, 19.25)	99.59 (99.46, 99.73)
8	8890	128	Nottingham-Cox-quant	42.97 (40.27, 45.67)	957.03 (954.33, 959.73)	9.11 (7.09, 10.8)	5.29 (3.82, 7.09)	63.28 (54.26, 71.06)	96.56 (96.25, 96.87)	21.2 (16.62, 25.19)	99.45 (99.26, 99.6)
9	8890	128	FIT-spline	60.85 (56.13, 65.69)	939.15 (934.31, 943.87)	10.12 (8.21, 12.15)	4.27 (2.92, 5.63)	70.31 (62.5, 78.32)	94.85 (94.38, 95.29)	16.64 (13.67, 19.78)	99.54 (99.4, 99.69)
9	8890	128	Nottingham-Cox-quant	38.47 (35.88, 40.94)	961.53 (959.06, 964.12)	8.21 (6.41, 9.9)	6.19 (4.61, 7.99)	57.03 (48.41, 65.25)	96.93 (96.66, 97.22)	21.35 (17.01, 25.89)	99.36 (99.17, 99.52)
10	8890	128	FIT-spline	57.71 (53.32, 62.43)	942.29 (937.57, 946.68)	9.9 (7.99, 12.04)	4.5 (3.15, 5.85)	68.75 (60.97, 76.87)	95.15 (94.7, 95.55)	17.15 (14.02, 20.5)	99.52 (99.38, 99.67)

10	8890	128	Nottingham-Cox-quant	34.08 (31.61, 36.56)	965.92 (963.44, 968.39)	7.87 (6.07, 9.67)	6.52 (4.95, 8.32)	54.69 (46.22, 63.24)	97.34 (97.05, 97.59)	23.1 (18.03, 28.16)	99.32 (99.14, 99.49)
**Post-COVID (2021/05 - 2021/12)**											
0.5	7472	99	FIT-spline	186.43 (177.73, 195.66)	813.57 (804.34, 822.27)	12.31 (9.9, 14.72)	0.94 (0.27, 1.74)	92.93 (87.38, 97.73)	82.35 (81.48, 83.16)	6.6 (5.36, 7.85)	99.88 (99.79, 99.97)
0.5	7472	99	Nottingham-Cox-quant	187.9 (180.81, 194.06)	812.1 (805.94, 819.19)	12.31 (9.9, 14.72)	0.94 (0.27, 1.74)	92.93 (87.38, 97.73)	82.21 (81.62, 82.9)	6.55 (5.33, 7.85)	99.88 (99.79, 99.97)
0.6	7472	99	FIT-spline	159.4 (151.37, 168.23)	840.6 (831.77, 848.63)	12.31 (9.9, 14.72)	0.94 (0.27, 1.74)	92.93 (87.38, 97.73)	85.09 (84.25, 85.83)	7.72 (6.27, 9.16)	99.89 (99.79, 99.97)
0.6	7472	99	Nottingham-Cox-quant	179.34 (173.05, 185.09)	820.66 (814.91, 826.95)	12.31 (9.9, 14.72)	0.94 (0.27, 1.74)	92.93 (87.38, 97.73)	83.07 (82.49, 83.68)	6.87 (5.58, 8.23)	99.89 (99.79, 99.97)
1	7472	99	FIT-spline	130.75 (123.26, 138.92)	869.25 (861.08, 876.74)	12.04 (9.77, 14.46)	1.2 (0.54, 2.01)	90.91 (84.88, 96.3)	87.97 (87.2, 88.71)	9.21 (7.54, 10.93)	99.86 (99.77, 99.94)
1	7472	99	Nottingham-Cox-quant	160.06 (154.84, 163.81)	839.94 (836.19, 845.16)	12.18 (9.77, 14.72)	1.07 (0.4, 1.87)	91.92 (86.24, 96.94)	85.01 (84.61, 85.54)	7.61 (6.21, 9.14)	99.87 (99.78, 99.95)
2	7472	99	FIT-spline	106.53 (99.84, 113.89)	893.47 (886.11, 900.16)	11.11 (8.83, 13.39)	2.14 (1.2, 3.22)	83.84 (75.5, 90.39)	90.33 (89.65, 90.94)	10.43 (8.32, 12.47)	99.76 (99.64, 99.87)
2	7472	99	Nottingham-Cox-quant	122.46 (119.11, 126.34)	877.54 (873.66, 880.89)	11.64 (9.37, 14.05)	1.61 (0.8, 2.54)	87.88 (81.55, 94.12)	88.77 (88.35, 89.12)	9.51 (7.66, 11.41)	99.82 (99.71, 99.91)
3	7472	99	FIT-spline	94.49 (88.19, 101.31)	905.51 (898.69, 911.81)	10.44 (8.16, 12.58)	2.81 (1.74, 4.02)	78.79 (70.29, 86.24)	91.48 (90.84, 92.08)	11.05 (8.79, 13.32)	99.69 (99.55, 99.81)
3	7472	99	Nottingham-Cox-quant	99.84 (96.22, 103.19)	900.16 (896.81, 903.78)	11.24 (8.97, 13.52)	2.01 (1.07, 3.08)	84.85 (77.22, 91.58)	91.02 (90.65, 91.42)	11.26 (9.1, 13.58)	99.78 (99.66, 99.88)
4	7472	99	FIT-spline	81.5 (75.48, 87.79)	918.5 (912.21, 924.52)	9.77 (7.63, 11.91)	3.48 (2.28, 4.82)	73.74 (65.04, 81.82)	92.73 (92.12, 93.3)	11.99 (9.48, 14.5)	99.62 (99.48, 99.75)
4	7472	99	Nottingham-Cox-quant	81.91 (78.83, 86.05)	918.09 (913.95, 921.17)	10.44 (8.16, 12.71)	2.81 (1.61, 4.01)	78.79 (70.64, 87.34)	92.76 (92.29, 93.11)	12.75 (10.03, 15.4)	99.69 (99.56, 99.82)
5	7472	99	FIT-spline	74.95 (69.46, 81.37)	925.05 (918.63, 930.54)	9.77 (7.63, 11.91)	3.48 (2.28, 4.82)	73.74 (65.04, 81.82)	93.39 (92.8, 93.94)	13.04 (10.33, 15.75)	99.62 (99.48, 99.76)
5	7472	99	Nottingham-Cox-quant	69.73 (66.38, 72.54)	930.27 (927.46, 933.62)	9.9 (7.9, 12.04)	3.35 (2.0, 4.68)	74.75 (66.67, 83.56)	93.94 (93.62, 94.35)	14.2 (11.47, 17.37)	99.64 (99.5, 99.79)
6	7472	99	FIT-spline	67.72 (62.5, 73.48)	932.28 (926.52, 937.5)	9.37 (7.23, 11.51)	3.88 (2.54, 5.35)	70.71 (62.1, 79.52)	94.09 (93.53, 94.6)	13.83 (10.85, 16.6)	99.58 (99.43, 99.73)
6	7472	99	Nottingham-Cox-quant	58.35 (55.81, 61.83)	941.65 (938.17, 944.19)	9.5 (7.63, 11.64)	3.75 (2.41, 5.09)	71.72 (63.1, 80.58)	95.05 (94.66, 95.37)	16.28 (12.8, 19.92)	99.6 (99.46, 99.74)
7	7472	99	FIT-spline	61.96 (57.01, 67.45)	938.04 (932.55, 942.99)	9.1 (6.96, 11.24)	4.15 (2.81, 5.62)	68.69 (59.55, 78.22)	94.64 (94.09, 95.11)	14.69 (11.38, 17.76)	99.56 (99.4, 99.7)
7	7472	99	Nottingham-Cox-quant	51.26 (48.05, 54.34)	948.74 (945.66, 951.95)	9.37 (7.36, 11.51)	3.88 (2.54, 5.35)	70.71 (61.96, 79.21)	95.75 (95.44, 96.13)	18.28 (14.29, 22.5)	99.59 (99.44, 99.73)
8	7472	99	FIT-spline	55.41 (50.59, 60.76)	944.59 (939.24, 949.41)	9.1 (6.96, 11.24)	4.15 (2.81, 5.62)	68.69 (59.55, 78.22)	95.31 (94.77, 95.77)	16.43 (12.7, 19.8)	99.56 (99.4, 99.7)
8	7472	99	Nottingham-Cox-quant	44.97 (42.29, 48.18)	955.03 (951.82, 957.71)	8.97 (7.23, 10.97)	4.28 (2.68, 5.89)	67.68 (58.69, 77.32)	96.35 (96.04, 96.66)	19.94 (16.07, 24.1)	99.55 (99.38, 99.72)
9	7472	99	FIT-spline	51.39 (46.57, 56.48)	948.61 (943.52, 953.43)	8.83 (6.83, 10.84)	4.42 (2.94, 6.02)	66.67 (57.13, 75.7)	95.69 (95.21, 96.14)	17.19 (13.5, 20.76)	99.53 (99.37, 99.69)
9	7472	99	Nottingham-Cox-quant	39.88 (37.2, 43.09)	960.12 (956.91, 962.8)	8.57 (6.69, 10.57)	4.68 (3.08, 6.29)	64.65 (55.0, 74.03)	96.83 (96.53, 97.11)	21.48 (17.12, 25.9)	99.51 (99.34, 99.68)
10	7472	99	FIT-spline	47.38 (42.69, 52.19)	952.62 (947.81, 957.31)	8.57 (6.69, 10.71)	4.68 (3.21, 6.16)	64.65 (55.29, 74.03)	96.07 (95.62, 96.5)	18.08 (14.12, 21.98)	99.51 (99.35, 99.66)
10	7472	99	Nottingham-Cox-quant	37.07 (34.4, 40.02)	962.93 (959.98, 965.6)	8.3 (6.42, 10.17)	4.95 (3.35, 6.83)	62.63 (52.94, 71.58)	97.08 (96.79, 97.36)	22.38 (17.23, 27.11)	99.49 (99.29, 99.65)
**2022 H1 (2022/01 - 2022/06)**											
0.5	5972	88	FIT-spline	196.08 (186.03, 206.63)	803.92 (793.37, 813.97)	13.4 (10.72, 16.41)	1.34 (0.5, 2.34)	90.91 (84.61, 96.39)	81.46 (80.37, 82.49)	6.83 (5.51, 8.3)	99.83 (99.71, 99.94)
0.5	5972	88	Nottingham-Cox-quant	210.82 (203.78, 217.52)	789.18 (782.48, 796.22)	13.73 (11.05, 16.91)	1.0 (0.33, 1.84)	93.18 (87.21, 97.78)	80.0 (79.32, 80.72)	6.51 (5.29, 8.01)	99.87 (99.77, 99.96)
0.6	5972	88	FIT-spline	175.82 (166.11, 185.87)	824.18 (814.13, 833.89)	13.4 (10.72, 16.41)	1.34 (0.5, 2.34)	90.91 (84.61, 96.39)	83.51 (82.5, 84.47)	7.62 (6.13, 9.29)	99.84 (99.72, 99.94)
0.6	5972	88	Nottingham-Cox-quant	197.76 (191.73, 204.12)	802.24 (795.88, 808.27)	13.73 (11.05, 16.91)	1.0 (0.33, 1.84)	93.18 (87.21, 97.78)	81.32 (80.66, 81.93)	6.94 (5.63, 8.49)	99.87 (99.77, 99.96)
1	5972	88	FIT-spline	145.01 (136.97, 154.22)	854.99 (845.78, 863.03)	13.4 (10.72, 16.41)	1.34 (0.5, 2.34)	90.91 (84.61, 96.39)	86.64 (85.74, 87.47)	9.24 (7.43, 11.24)	99.84 (99.73, 99.94)
1	5972	88	Nottingham-Cox-quant	161.59 (157.74, 167.12)	838.41 (832.88, 842.26)	13.4 (10.72, 16.41)	1.34 (0.5, 2.34)	90.91 (84.78, 96.63)	84.96 (84.35, 85.41)	8.29 (6.62, 10.13)	99.84 (99.72, 99.94)
2	5972	88	FIT-spline	115.2 (107.84, 123.58)	884.8 (876.42, 892.16)	13.06 (10.38, 16.24)	1.67 (0.84, 2.68)	88.64 (81.7, 94.51)	89.63 (88.86, 90.37)	11.34 (9.04, 13.88)	99.81 (99.7, 99.91)
2	5972	88	Nottingham-Cox-quant	113.86 (111.19, 119.06)	886.14 (880.94, 888.81)	13.23 (10.55, 16.24)	1.51 (0.67, 2.51)	89.77 (82.72, 95.29)	89.79 (89.19, 90.15)	11.62 (9.17, 14.18)	99.83 (99.72, 99.92)
3	5972	88	FIT-spline	98.46 (91.43, 106.33)	901.54 (893.67, 908.57)	13.06 (10.38, 16.24)	1.67 (0.84, 2.68)	88.64 (81.7, 94.51)	91.33 (90.58, 91.99)	13.27 (10.56, 16.14)	99.81 (99.7, 99.91)
3	5972	88	Nottingham-Cox-quant	90.42 (85.4, 93.6)	909.58 (906.4, 914.6)	12.73 (10.05, 15.74)	2.01 (1.0, 3.18)	86.36 (78.79, 93.41)	92.11 (91.71, 92.78)	14.07 (11.29, 17.68)	99.78 (99.65, 99.89)
4	5972	88	FIT-spline	83.05 (76.19, 89.92)	916.95 (910.08, 923.81)	12.39 (9.71, 15.41)	2.34 (1.17, 3.68)	84.09 (75.67, 91.21)	92.83 (92.13, 93.48)	14.92 (11.81, 18.22)	99.74 (99.6, 99.87)
4	5972	88	Nottingham-Cox-quant	73.34 (69.99, 77.03)	926.66 (922.97, 930.01)	12.56 (9.88, 15.24)	2.18 (1.0, 3.52)	85.23 (76.62, 92.23)	93.83 (93.4, 94.22)	17.12 (13.54, 20.95)	99.77 (99.62, 99.89)
5	5972	88	FIT-spline	75.52 (68.99, 82.22)	924.48 (917.78, 931.01)	11.72 (9.04, 14.74)	3.01 (1.67, 4.52)	79.55 (70.82, 87.5)	93.52 (92.86, 94.14)	15.52 (12.3, 19.07)	99.67 (99.51, 99.82)
5	5972	88	Nottingham-Cox-quant	62.79 (59.11, 65.81)	937.21 (934.19, 940.89)	11.89 (9.38, 14.57)	2.85 (1.51, 4.35)	80.68 (71.79, 89.02)	94.83 (94.43, 95.25)	18.93 (14.95, 23.22)	99.7 (99.53, 99.84)
6	5972	88	FIT-spline	68.49 (62.29, 75.18)	931.51 (924.82, 937.71)	11.05 (8.54, 13.9)	3.68 (2.18, 5.19)	75.0 (65.47, 83.78)	94.17 (93.57, 94.74)	16.14 (12.66, 19.95)	99.6 (99.44, 99.77)
6	5972	88	Nottingham-Cox-quant	54.92 (51.07, 57.94)	945.08 (942.06, 948.93)	11.22 (8.7, 13.73)	3.52 (2.18, 5.36)	76.14 (64.91, 84.62)	95.56 (95.19, 95.98)	20.43 (15.82, 25.07)	99.63 (99.43, 99.77)
7	5972	88	FIT-spline	61.79 (55.93, 67.82)	938.21 (932.18, 944.07)	10.72 (8.2, 13.4)	4.02 (2.51, 5.53)	72.73 (62.76, 81.58)	94.82 (94.22, 95.34)	17.34 (13.53, 21.47)	99.57 (99.41, 99.73)
7	5972	88	Nottingham-Cox-quant	46.38 (43.03, 49.57)	953.62 (950.43, 956.97)	10.05 (7.87, 12.56)	4.69 (3.01, 6.53)	68.18 (58.54, 78.72)	96.31 (95.95, 96.7)	21.66 (16.83, 26.89)	99.51 (99.32, 99.69)
8	5972	88	FIT-spline	56.93 (51.57, 62.79)	943.07 (937.21, 948.43)	10.21 (7.7, 12.89)	4.52 (2.85, 6.2)	69.32 (59.77, 78.65)	95.26 (94.71, 95.75)	17.94 (13.88, 22.09)	99.52 (99.34, 99.7)
8	5972	88	Nottingham-Cox-quant	40.86 (37.68, 43.88)	959.14 (956.12, 962.32)	9.54 (7.54, 11.89)	5.19 (3.35, 7.2)	64.77 (54.54, 75.0)	96.82 (96.47, 97.17)	23.36 (18.01, 29.29)	99.46 (99.25, 99.65)
9	5972	88	FIT-spline	51.57 (46.55, 57.1)	948.43 (942.9, 953.45)	10.05 (7.54, 12.56)	4.69 (3.18, 6.36)	68.18 (58.44, 77.65)	95.79 (95.24, 96.28)	19.48 (15.13, 24.14)	99.51 (99.33, 99.66)
9	5972	88	Nottingham-Cox-quant	36.34 (32.99, 39.52)	963.66 (960.48, 967.01)	9.04 (6.87, 11.22)	5.69 (4.02, 7.87)	61.36 (50.6, 71.05)	97.23 (96.84, 97.58)	24.88 (18.5, 31.1)	99.41 (99.19, 99.58)
10	5972	88	FIT-spline	49.23 (44.21, 54.76)	950.77 (945.24, 955.79)	9.71 (7.37, 12.22)	5.02 (3.35, 6.7)	65.91 (55.78, 75.79)	95.99 (95.48, 96.47)	19.73 (15.18, 24.6)	99.47 (99.29, 99.65)
10	5972	88	Nottingham-Cox-quant	31.82 (29.3, 35.5)	968.18 (964.5, 970.7)	8.37 (6.2, 10.88)	6.36 (4.35, 8.37)	56.82 (46.66, 67.05)	97.62 (97.23, 97.89)	26.32 (19.48, 32.96)	99.34 (99.14, 99.55)
**2022 H2 (2022/07 - 2022/12)**											
0.5	7490	104	FIT-spline	203.74 (194.39, 212.82)	796.26 (787.18, 805.61)	13.08 (10.81, 15.89)	0.8 (0.27, 1.6)	94.23 (89.28, 98.11)	80.67 (79.71, 81.57)	6.42 (5.31, 7.7)	99.9 (99.8, 99.97)
0.5	7490	104	Nottingham-Cox-quant	212.82 (206.81, 218.43)	787.18 (781.57, 793.19)	13.08 (10.81, 15.89)	0.8 (0.27, 1.6)	94.23 (89.28, 98.11)	79.75 (79.16, 80.33)	6.15 (5.08, 7.38)	99.9 (99.8, 99.97)
0.6	7490	104	FIT-spline	203.6 (194.39, 212.69)	796.4 (787.31, 805.61)	13.08 (10.81, 15.89)	0.8 (0.27, 1.6)	94.23 (89.28, 98.11)	80.68 (79.74, 81.59)	6.43 (5.32, 7.7)	99.9 (99.8, 99.97)
0.6	7490	104	Nottingham-Cox-quant	197.86 (192.52, 202.54)	802.14 (797.46, 807.48)	13.08 (10.81, 15.89)	0.8 (0.27, 1.6)	94.23 (89.28, 98.11)	81.26 (80.76, 81.81)	6.61 (5.48, 7.97)	99.9 (99.8, 99.97)
1	7490	104	FIT-spline	165.69 (157.27, 174.1)	834.31 (825.9, 842.73)	12.82 (10.55, 15.49)	1.07 (0.4, 1.87)	92.31 (86.95, 96.97)	84.5 (83.65, 85.35)	7.74 (6.39, 9.26)	99.87 (99.78, 99.95)
1	7490	104	Nottingham-Cox-quant	158.21 (153.53, 162.08)	841.79 (837.92, 846.47)	12.95 (10.68, 15.62)	0.93 (0.4, 1.74)	93.27 (87.9, 97.48)	85.27 (84.87, 85.77)	8.19 (6.77, 9.88)	99.89 (99.79, 99.95)
2	7490	104	FIT-spline	124.57 (116.82, 131.78)	875.43 (868.22, 883.18)	12.02 (9.75, 14.69)	1.87 (0.93, 2.94)	86.54 (80.0, 93.14)	88.59 (87.85, 89.38)	9.65 (7.88, 11.66)	99.79 (99.67, 99.89)
2	7490	104	Nottingham-Cox-quant	111.08 (107.48, 115.09)	888.92 (884.91, 892.52)	12.02 (9.88, 14.55)	1.87 (0.93, 2.94)	86.54 (80.0, 92.93)	89.95 (89.47, 90.42)	10.82 (8.89, 13.28)	99.79 (99.67, 99.89)
3	7490	104	FIT-spline	107.21 (99.87, 114.29)	892.79 (885.71, 900.13)	11.35 (9.21, 13.89)	2.54 (1.47, 3.74)	81.73 (74.31, 88.98)	90.28 (89.55, 91.02)	10.59 (8.59, 12.72)	99.72 (99.58, 99.84)
3	7490	104	Nottingham-Cox-quant	87.58 (84.51, 91.19)	912.42 (908.81, 915.49)	11.48 (9.35, 14.02)	2.4 (1.34, 3.6)	82.69 (75.86, 90.0)	92.28 (91.87, 92.63)	13.11 (10.71, 15.96)	99.74 (99.61, 99.85)
4	7490	104	FIT-spline	90.79 (83.84, 97.46)	909.21 (902.54, 916.16)	10.55 (8.28, 12.95)	3.34 (2.14, 4.54)	75.96 (67.96, 83.79)	91.86 (91.23, 92.51)	11.62 (9.36, 14.16)	99.63 (99.5, 99.76)
4	7490	104	Nottingham-Cox-quant	72.76 (69.16, 76.23)	927.24 (923.77, 930.84)	11.08 (9.08, 13.48)	2.8 (1.6, 4.01)	79.81 (72.73, 87.64)	93.74 (93.37, 94.14)	15.23 (12.34, 18.57)	99.7 (99.57, 99.83)
5	7490	104	FIT-spline	78.77 (72.23, 85.05)	921.23 (914.95, 927.77)	10.15 (8.01, 12.55)	3.74 (2.53, 5.21)	73.08 (65.06, 81.37)	93.04 (92.45, 93.67)	12.88 (10.39, 15.66)	99.59 (99.44, 99.73)
5	7490	104	Nottingham-Cox-quant	60.48 (56.74, 63.42)	939.52 (936.58, 943.26)	10.68 (8.68, 13.08)	3.2 (2.0, 4.54)	76.92 (68.52, 84.75)	94.95 (94.64, 95.32)	17.66 (14.25, 21.57)	99.66 (99.52, 99.79)
6	7490	104	FIT-spline	68.49 (62.22, 74.63)	931.51 (925.37, 937.78)	9.35 (7.34, 11.62)	4.54 (3.07, 6.14)	67.31 (58.62, 76.19)	94.0 (93.44, 94.59)	13.65 (10.94, 16.73)	99.51 (99.34, 99.67)
6	7490	104	Nottingham-Cox-quant	51.94 (48.87, 55.14)	948.06 (944.86, 951.13)	9.88 (8.01, 12.15)	4.01 (2.53, 5.47)	71.15 (62.74, 80.61)	95.74 (95.38, 96.06)	19.02 (15.38, 23.14)	99.58 (99.42, 99.73)
7	7490	104	FIT-spline	61.15 (55.27, 66.76)	938.85 (933.24, 944.73)	8.81 (6.68, 11.08)	5.07 (3.6, 6.81)	63.46 (54.31, 72.44)	94.69 (94.18, 95.23)	14.41 (11.38, 17.74)	99.46 (99.28, 99.62)
7	7490	104	Nottingham-Cox-quant	45.26 (41.92, 47.93)	954.74 (952.07, 958.08)	9.21 (7.21, 11.35)	4.67 (3.34, 6.28)	66.35 (57.14, 74.16)	96.34 (96.04, 96.7)	20.35 (16.12, 25.0)	99.51 (99.34, 99.65)

8	7490	104	FIT-spline	56.74 (51.13, 62.22)	943.26 (937.78, 948.87)	8.81 (6.68, 11.08)	5.07 (3.6, 6.81)	63.46 (54.31, 72.44)	95.14 (94.67, 95.64)	15.53 (12.35, 19.11)	99.46 (99.28, 99.62)
8	7490	104	Nottingham-Cox-quant	38.32 (35.11, 40.86)	961.68 (959.14, 964.89)	8.95 (6.94, 10.95)	4.94 (3.6, 6.81)	64.42 (54.86, 71.91)	97.02 (96.73, 97.34)	23.34 (18.33, 28.42)	99.49 (99.29, 99.63)
9	7490	104	FIT-spline	52.2 (47.0, 57.28)	947.8 (942.72, 953.0)	8.81 (6.68, 11.08)	5.07 (3.6, 6.81)	63.46 (54.31, 72.44)	95.6 (95.16, 96.07)	16.88 (13.47, 20.63)	99.46 (99.28, 99.62)
9	7490	104	Nottingham-Cox-quant	32.98 (30.57, 36.18)	967.02 (963.82, 969.43)	8.54 (6.81, 10.68)	5.34 (3.74, 7.08)	61.54 (53.19, 70.3)	97.52 (97.24, 97.75)	25.91 (20.46, 31.5)	99.45 (99.27, 99.61)
10	7490	104	FIT-spline	48.06 (42.72, 52.87)	951.94 (947.13, 957.28)	8.41 (6.41, 10.55)	5.47 (3.87, 7.21)	60.58 (51.16, 69.17)	95.98 (95.56, 96.45)	17.5 (13.92, 21.57)	99.42 (99.24, 99.59)
10	7490	104	Nottingham-Cox-quant	30.31 (27.9, 33.11)	969.69 (966.89, 972.1)	7.88 (6.28, 9.88)	6.01 (4.27, 7.74)	56.73 (48.31, 66.35)	97.73 (97.45, 97.97)	25.99 (20.78, 31.7)	99.38 (99.2, 99.56)
**2023 H1 (2023/01 - 2023/06)**											
0.5	8320	91	FIT-spline	205.53 (197.48, 214.18)	794.47 (785.82, 802.52)	10.1 (7.81, 12.26)	0.84 (0.24, 1.44)	92.31 (86.87, 97.53)	80.24 (79.4, 81.03)	4.91 (3.87, 5.9)	99.89 (99.82, 99.97)
0.5	8320	91	Nottingham-Cox-quant	219.71 (213.7, 224.52)	780.29 (775.48, 786.3)	10.22 (8.05, 12.38)	0.72 (0.24, 1.32)	93.41 (88.24, 97.85)	78.82 (78.32, 79.42)	4.65 (3.71, 5.63)	99.91 (99.83, 99.97)
0.6	8320	91	FIT-spline	193.03 (185.1, 201.45)	806.97 (798.55, 814.9)	10.1 (7.81, 12.26)	0.84 (0.24, 1.44)	92.31 (86.87, 97.53)	81.5 (80.7, 82.28)	5.23 (4.12, 6.29)	99.9 (99.82, 99.97)
0.6	8320	91	Nottingham-Cox-quant	201.2 (196.63, 205.17)	798.8 (794.83, 803.37)	10.1 (7.93, 12.26)	0.84 (0.24, 1.56)	92.31 (86.6, 97.56)	80.68 (80.26, 81.16)	5.02 (3.95, 6.07)	99.89 (99.8, 99.97)
1	8320	91	FIT-spline	159.62 (152.28, 167.43)	840.38 (832.57, 847.72)	10.1 (7.81, 12.26)	0.84 (0.24, 1.44)	92.31 (86.87, 97.53)	84.88 (84.11, 85.59)	6.33 (5.0, 7.59)	99.9 (99.83, 99.97)
1	8320	91	Nottingham-Cox-quant	156.37 (152.64, 160.58)	843.63 (839.42, 847.36)	9.86 (7.69, 12.02)	1.08 (0.36, 1.8)	90.11 (84.09, 96.05)	85.19 (84.68, 85.61)	6.3 (4.89, 7.74)	99.87 (99.79, 99.96)
2	8320	91	FIT-spline	118.27 (111.78, 125.24)	881.73 (874.76, 888.22)	9.38 (7.33, 11.54)	1.56 (0.72, 2.4)	85.71 (78.38, 92.86)	88.99 (88.3, 89.63)	7.93 (6.21, 9.66)	99.82 (99.73, 99.92)
2	8320	91	Nottingham-Cox-quant	108.05 (103.97, 111.3)	891.95 (888.7, 896.03)	9.62 (7.69, 11.66)	1.32 (0.6, 2.04)	87.91 (81.71, 94.45)	90.05 (89.67, 90.51)	8.9 (7.07, 10.94)	99.85 (99.77, 99.93)
3	8320	91	FIT-spline	98.08 (92.06, 104.45)	901.92 (895.55, 907.94)	8.77 (6.85, 10.94)	2.16 (1.2, 3.12)	80.22 (72.11, 88.1)	90.97 (90.35, 91.56)	8.95 (7.0, 10.94)	99.76 (99.65, 99.87)
3	8320	91	Nottingham-Cox-quant	84.38 (81.37, 87.62)	915.62 (912.38, 918.63)	8.65 (6.85, 10.46)	2.28 (1.32, 3.49)	79.12 (70.0, 87.5)	92.34 (91.97, 92.67)	10.26 (7.97, 12.36)	99.75 (99.62, 99.86)
4	8320	91	FIT-spline	82.69 (76.68, 88.34)	917.31 (911.66, 923.32)	8.05 (6.25, 9.98)	2.88 (1.68, 3.97)	73.63 (64.58, 82.72)	92.45 (91.91, 93.01)	9.74 (7.68, 12.0)	99.69 (99.57, 99.82)
4	8320	91	Nottingham-Cox-quant	68.27 (65.38, 71.52)	931.73 (928.48, 934.62)	7.57 (5.77, 9.25)	3.37 (2.16, 4.69)	69.23 (60.0, 78.16)	93.86 (93.48, 94.17)	11.09 (8.41, 13.52)	99.64 (99.5, 99.77)
5	8320	91	FIT-spline	70.91 (65.26, 76.57)	929.09 (923.43, 934.74)	7.81 (6.01, 9.86)	3.12 (1.92, 4.33)	71.43 (62.79, 80.73)	93.62 (93.09, 94.15)	11.02 (8.63, 13.57)	99.66 (99.54, 99.79)
5	8320	91	Nottingham-Cox-quant	56.01 (53.12, 59.01)	943.99 (940.99, 946.88)	6.85 (5.17, 8.65)	4.09 (2.64, 5.53)	62.64 (53.48, 72.98)	95.03 (94.69, 95.37)	12.23 (9.25, 15.33)	99.57 (99.42, 99.72)
6	8320	91	FIT-spline	63.46 (58.29, 68.51)	936.54 (931.49, 941.71)	7.33 (5.65, 9.25)	3.61 (2.28, 4.93)	67.03 (58.0, 77.38)	94.32 (93.84, 94.85)	11.55 (8.96, 14.36)	99.61 (99.47, 99.76)
6	8320	91	Nottingham-Cox-quant	48.68 (45.91, 51.2)	951.32 (948.8, 954.09)	6.49 (4.93, 8.17)	4.45 (3.0, 5.77)	59.34 (49.53, 70.11)	95.73 (95.46, 96.06)	13.33 (10.1, 16.92)	99.53 (99.39, 99.68)
7	8320	91	FIT-spline	56.85 (51.92, 61.78)	943.15 (938.22, 948.08)	6.85 (5.17, 8.65)	4.09 (2.76, 5.53)	62.64 (53.16, 72.62)	94.94 (94.49, 95.42)	12.05 (9.21, 15.13)	99.57 (99.42, 99.71)
7	8320	91	Nottingham-Cox-quant	41.11 (38.94, 44.23)	958.89 (955.77, 961.06)	6.13 (4.68, 7.81)	4.81 (3.24, 6.25)	56.04 (46.53, 67.44)	96.46 (96.14, 96.73)	14.91 (11.27, 18.86)	99.5 (99.35, 99.66)
8	8320	91	FIT-spline	50.6 (45.91, 55.29)	949.4 (944.71, 954.09)	6.13 (4.57, 7.93)	4.81 (3.37, 6.25)	56.04 (46.57, 66.3)	95.5 (95.04, 95.96)	12.11 (9.15, 15.42)	99.49 (99.34, 99.65)
8	8320	91	Nottingham-Cox-quant	36.9 (33.77, 39.18)	963.1 (960.82, 966.23)	6.01 (4.45, 7.69)	4.93 (3.37, 6.49)	54.95 (45.45, 66.27)	96.88 (96.65, 97.2)	16.29 (12.33, 20.96)	99.49 (99.33, 99.65)
9	8320	91	FIT-spline	46.51 (42.18, 51.08)	953.49 (948.92, 957.82)	5.65 (4.09, 7.33)	5.29 (3.73, 6.85)	51.65 (41.3, 62.62)	95.87 (95.42, 96.29)	12.14 (8.91, 15.68)	99.45 (99.28, 99.61)
9	8320	91	Nottingham-Cox-quant	32.45 (29.45, 34.86)	967.55 (965.14, 970.55)	5.77 (4.21, 7.45)	5.17 (3.61, 6.73)	52.75 (43.27, 63.73)	97.3 (97.08, 97.59)	17.78 (13.67, 22.66)	99.47 (99.3, 99.63)
10	8320	91	FIT-spline	43.51 (39.06, 47.96)	956.49 (952.04, 960.94)	5.41 (3.85, 6.97)	5.53 (3.97, 7.21)	49.45 (38.77, 60.26)	96.15 (95.72, 96.57)	12.43 (8.99, 16.0)	99.42 (99.25, 99.59)
10	8320	91	Nottingham-Cox-quant	29.45 (26.8, 31.85)	970.55 (968.15, 973.2)	5.29 (3.97, 6.85)	5.65 (3.97, 7.21)	48.35 (39.32, 59.35)	97.56 (97.34, 97.82)	17.96 (13.71, 23.01)	99.42 (99.26, 99.59)
**All data (2017/01 - 2023/08)**											
0.5	51477	659	FIT-spline	195.04 (191.68, 198.42)	804.96 (801.58, 808.32)	608.0 (558.98, 655.02)	51.0 (38.0, 65.0)	92.26 (90.22, 94.2)	81.44 (81.11, 81.77)	6.06 (5.58, 6.48)	99.88 (99.84, 99.91)
0.5	51477	659	Nottingham-Cox-quant	210.09 (204.81, 211.59)	789.91 (788.41, 795.19)	616.0 (564.98, 662.0)	43.0 (31.98, 57.0)	93.47 (91.34, 95.15)	79.93 (79.76, 80.46)	5.7 (5.26, 6.11)	99.89 (99.86, 99.92)
0.6	51477	659	FIT-spline	175.46 (172.1, 178.7)	824.54 (821.3, 827.9)	603.0 (554.0, 650.0)	56.0 (42.0, 71.0)	91.5 (89.21, 93.44)	83.41 (83.1, 83.74)	6.68 (6.15, 7.15)	99.87 (99.83, 99.9)
0.6	51477	659	Nottingham-Cox-quant	197.14 (192.4, 198.54)	802.86 (801.46, 807.6)	614.0 (564.98, 659.0)	45.0 (33.0, 59.0)	93.17 (91.08, 94.91)	81.24 (81.08, 81.72)	6.05 (5.59, 6.52)	99.89 (99.86, 99.92)
1	51477	659	FIT-spline	148.24 (145.11, 151.39)	851.76 (848.61, 854.89)	592.0 (544.0, 638.0)	67.0 (51.0, 83.0)	89.83 (87.52, 92.06)	86.15 (85.83, 86.45)	7.76 (7.15, 8.31)	99.85 (99.81, 99.88)
1	51477	659	Nottingham-Cox-quant	160.79 (159.04, 162.68)	839.21 (837.32, 840.96)	605.0 (556.0, 652.0)	54.0 (40.0, 68.0)	91.81 (89.67, 93.82)	84.9 (84.71, 85.09)	7.31 (6.72, 7.86)	99.88 (99.84, 99.91)
2	51477	659	FIT-spline	111.86 (109.17, 114.65)	888.14 (885.35, 890.83)	566.0 (518.98, 610.0)	93.0 (73.97, 112.0)	85.89 (83.13, 88.66)	89.78 (89.51, 90.04)	9.83 (9.03, 10.53)	99.8 (99.76, 99.84)
2	51477	659	Nottingham-Cox-quant	113.74 (112.46, 115.47)	886.26 (884.53, 887.54)	579.0 (532.98, 624.0)	80.0 (63.0, 97.02)	87.86 (85.37, 90.36)	89.62 (89.43, 89.76)	9.89 (9.05, 10.61)	99.82 (99.79, 99.86)
3	51477	659	FIT-spline	94.24 (91.75, 96.74)	905.76 (903.26, 908.25)	541.0 (495.98, 583.05)	118.0 (98.0, 140.0)	82.09 (79.15, 84.96)	91.52 (91.27, 91.76)	11.15 (10.25, 11.97)	99.75 (99.7, 99.79)
3	51477	659	Nottingham-Cox-quant	89.15 (87.79, 90.49)	910.85 (909.51, 912.21)	551.0 (504.0, 593.02)	108.0 (88.0, 129.0)	83.61 (80.83, 86.3)	92.05 (91.89, 92.2)	12.01 (11.01, 12.92)	99.77 (99.72, 99.81)
4	51477	659	FIT-spline	81.65 (79.3, 83.94)	918.35 (916.06, 920.7)	520.0 (477.0, 563.0)	139.0 (117.0, 163.0)	78.91 (75.81, 82.02)	92.75 (92.53, 92.97)	12.37 (11.37, 13.34)	99.71 (99.66, 99.75)
4	51477	659	Nottingham-Cox-quant	72.65 (71.59, 74.05)	927.35 (925.95, 928.41)	517.0 (476.95, 561.0)	142.0 (116.0, 163.02)	78.45 (75.58, 82.02)	93.66 (93.5, 93.78)	13.82 (12.69, 14.97)	99.7 (99.66, 99.76)
5	51477	659	FIT-spline	72.63 (70.4, 74.85)	927.37 (925.15, 929.6)	493.0 (450.0, 536.0)	166.0 (141.0, 192.0)	74.81 (71.47, 77.97)	93.61 (93.4, 93.83)	13.19 (12.1, 14.23)	99.65 (99.6, 99.7)
5	51477	659	Nottingham-Cox-quant	61.37 (60.24, 62.55)	938.63 (937.45, 939.76)	491.0 (449.0, 533.0)	168.0 (141.0, 194.0)	74.51 (71.34, 78.02)	94.75 (94.61, 94.88)	15.54 (14.25, 16.87)	99.65 (99.6, 99.71)
6	51477	659	FIT-spline	64.55 (62.42, 66.69)	935.45 (933.31, 937.58)	473.0 (431.98, 516.02)	186.0 (160.0, 213.02)	71.78 (68.3, 75.0)	94.39 (94.2, 94.6)	14.23 (13.07, 15.4)	99.61 (99.56, 99.67)
6	51477	659	Nottingham-Cox-quant	52.57 (51.44, 53.6)	947.43 (946.4, 948.56)	466.0 (422.0, 503.02)	193.0 (166.0, 222.02)	70.71 (67.12, 73.88)	95.59 (95.47, 95.72)	17.22 (15.76, 18.7)	99.6 (99.54, 99.66)
7	51477	659	FIT-spline	57.5 (55.54, 59.54)	942.5 (940.46, 944.46)	449.0 (410.98, 489.0)	210.0 (184.0, 239.0)	68.13 (64.58, 71.52)	95.06 (94.88, 95.25)	15.17 (13.95, 16.38)	99.57 (99.51, 99.62)
7	51477	659	Nottingham-Cox-quant	45.61 (44.58, 46.72)	954.39 (953.28, 955.42)	437.0 (397.98, 476.0)	222.0 (193.0, 253.0)	66.31 (62.65, 70.03)	96.24 (96.12, 96.36)	18.61 (16.98, 20.32)	99.55 (99.49, 99.61)
8	51477	659	FIT-spline	51.89 (50.02, 53.77)	948.11 (946.23, 949.98)	424.0 (386.0, 464.0)	235.0 (208.0, 265.0)	64.34 (60.73, 67.8)	95.58 (95.4, 95.75)	15.87 (14.55, 17.19)	99.52 (99.46, 99.57)
8	51477	659	Nottingham-Cox-quant	39.96 (38.89, 40.99)	960.04 (959.01, 961.11)	414.0 (374.0, 451.0)	245.0 (214.0, 279.0)	62.82 (59.12, 66.67)	96.77 (96.65, 96.88)	20.13 (18.34, 21.94)	99.5 (99.44, 99.57)
9	51477	659	FIT-spline	47.48 (45.65, 49.36)	952.52 (950.64, 954.35)	408.0 (370.98, 447.02)	251.0 (223.0, 282.0)	61.91 (58.35, 65.42)	95.99 (95.83, 96.16)	16.69 (15.26, 18.08)	99.49 (99.43, 99.55)
9	51477	659	Nottingham-Cox-quant	35.9 (34.64, 36.77)	964.1 (963.23, 965.36)	393.0 (355.98, 430.0)	266.0 (233.0, 300.0)	59.64 (55.92, 63.27)	97.14 (97.04, 97.27)	21.27 (19.39, 23.35)	99.46 (99.4, 99.53)
10	51477	659	FIT-spline	43.75 (41.86, 45.48)	956.25 (954.52, 958.14)	393.0 (357.0, 432.0)	266.0 (237.0, 298.02)	59.64 (56.05, 63.24)	96.34 (96.18, 96.51)	17.45 (15.99, 19.01)	99.46 (99.39, 99.52)
10	51477	659	Nottingham-Cox-quant	32.13 (31.02, 33.1)	967.87 (966.9, 968.98)	379.0 (344.0, 416.0)	280.0 (246.0, 314.02)	57.51 (53.82, 61.32)	97.49 (97.39, 97.6)	22.91 (20.81, 25.09)	99.44 (99.37, 99.51)
