# Supplementary material for: External validation of the COLOFIT colorectal cancer risk prediction model in the Oxford-FIT dataset: the importance of population characteristics and clinically relevant evaluation metrics
Source: BMC Med. 2025 Aug 27;23:503. doi: 10.1186/s12916-025-04339-w (PMC12392603; doi:10.1186/s12916-025-04339-w)
Supplement: Supplementary file 14 — Additional File 14: Diagnostic metrics at selected levels of sensitivity: Table S14. Tab S14 – Performance metrics computed at selected levels of sensitivity for the COLOFIT model and Oxford FIT-spline models [file 12916_2025_4339_MOESM14_ESM.pdf]

S14. DIAGNOSTIC METRICS AT SELECTED LEVELS OF SENSITIVITY

Common diagnostic metrics of model performance at various levels of sensitivity are reported for the recalibrated COLOFIT model and the FIT test in Table S14. Table S14 is based on interpolated true and false positive counts: if the empirical classification curve of true and false positive counts did not have a point where the sensitivity was exactly equal to the desired value (such as 90%), then false positive counts were interpolated using a method of Davis and Goadrich [31], and all other diagnostic metrics (such as PPV, NPV, sensitivity, specificity) were derived from the true and false positive counts and the total number of cancers. Sensitivities corresponding to FIT ≥ 10 µg/g are shown in red, but please note that the actual FIT threshold reported in the table for these sensitivities was not always exactly 10, as multiple FIT thresholds near 10 can yield the same level of sensitivity. For direct comparison of models against FIT at threshold 10 µg/g, please refer to “reduction in referrals” section of the main text.

Table S14. Performance metrics computed at selected levels of sensitivity for the COLOFIT model (Nottingham-Cox) and Oxford FIT-spline models

Sensitivity (%)	Num patients	Num cancers	Model	Positive tests per 1000 tests	Negative tests per 1000 tests	Detected cancers	Missed cancers	Specificity (%)	PPV (%)	NPV (%)	Threshold approx (%)
**Pre-COVID (2017/01 - 2020/02)**											
80	10379	124	FIT test	61.93 (46.26, 89.91)	938.07 (910.09, 953.74)	99.2 (82.4, 116.0)	24.8 (20.6, 29.0)	94.7 (91.83, 96.27)	15.43 (10.41, 21.3)	99.75 (99.7, 99.79)	21.75 (9.05, 35.83)
80	10379	124	Nottingham-Cox	59.08 (46.4, 93.28)	940.92 (906.72, 953.6)	99.2 (82.4, 116.0)	24.8 (20.6, 29.0)	94.99 (91.5, 96.13)	16.18 (10.37, 21.06)	99.75 (99.7, 99.79)	1.64 (0.5, 2.83)
85	10379	124	FIT test	81.9 (56.74, 148.2)	918.1 (851.8, 943.26)	105.4 (87.55, 123.25)	18.6 (15.45, 21.75)	92.74 (86.15, 95.27)	12.4 (6.14, 18.22)	99.8 (99.77, 99.84)	11.95 (3.15, 26.45)
85	10379	124	Nottingham-Cox	76.54 (55.87, 173.35)	923.46 (826.65, 944.13)	105.4 (87.55, 123.25)	18.6 (15.45, 21.75)	93.28 (83.41, 95.35)	13.27 (5.54, 19.08)	99.81 (99.76, 99.84)	0.86 (0.27, 1.87)
86.29	10379	124	FIT test	86.14 (62.24, 198.09)	913.86 (801.91, 937.76)	107.0 (107.0, 107.0)	17.0 (17.0, 17.0)	92.33 (80.99, 94.74)	11.97 (5.21, 16.66)	99.82 (99.8, 99.83)	10.4 (1.5, 21.6)
86.29	10379	124	Nottingham-Cox	86.52 (57.19, 182.31)	913.48 (817.69, 942.81)	107.0 (107.0, 107.0)	17.0 (17.0, 17.0)	92.29 (82.59, 95.26)	11.92 (5.74, 18.05)	99.82 (99.8, 99.83)	0.65 (0.25, 1.61)
90	10379	124	FIT test	144.6 (74.26, 438.5)	855.4 (561.5, 925.74)	111.6 (92.7, 130.5)	12.4 (10.3, 14.5)	86.45 (56.7, 93.58)	7.44 (2.33, 14.42)	99.86 (99.78, 99.89)	3.15 (0.65, 14.35)
90	10379	124	Nottingham-Cox	166.26 (68.29, 375.8)	833.74 (624.2, 931.71)	111.6 (92.7, 130.5)	12.4 (10.3, 14.5)	84.26 (63.1, 94.21)	6.47 (2.72, 15.53)	99.86 (99.79, 99.89)	0.28 (0.17, 1.13)
95	10379	124	FIT test	557.9 (144.57, 719.25)	442.1 (280.75, 855.43)	117.8 (97.85, 137.75)	6.2 (5.15, 7.25)	44.68 (28.35, 86.51)	2.03 (1.45, 7.76)	99.86 (99.78, 99.93)	0.65 (0.65, 3.15)
95	10379	124	Nottingham-Cox	380.36 (166.98, 685.06)	619.64 (314.94, 833.02)	117.8 (97.85, 137.75)	6.2 (5.15, 7.25)	62.65 (31.81, 84.19)	2.98 (1.65, 7.12)	99.9 (99.8, 99.93)	0.17 (0.09, 0.28)
99	10379	124	FIT test	911.58 (807.14, 943.85)	88.42 (56.15, 192.86)	122.76 (101.97, 143.55)	1.24 (1.03, 1.45)	8.94 (5.67, 19.51)	1.3 (1.09, 1.56)	99.86 (99.78, 99.94)	0.65 (0.65, 0.65)
99	10379	124	Nottingham-Cox	790.9 (526.5, 820.19)	209.1 (179.81, 473.5)	122.76 (101.97, 143.55)	1.24 (1.03, 1.45)	21.15 (18.18, 47.91)	1.5 (1.25, 2.39)	99.94 (99.93, 99.98)	0.07 (0.07, 0.13)
**COVID (2020/03 - 2021/04)**											
80	8890	128	FIT test	77.44 (63.51, 98.14)	922.56 (901.86, 936.49)	102.4 (85.6, 120.02)	25.6 (21.4, 30.0)	93.31 (91.15, 94.69)	14.88 (11.29, 18.71)	99.69 (99.63, 99.74)	16.6 (9.05, 27.65)
80	8890	128	Nottingham-Cox	71.14 (60.04, 89.48)	928.86 (910.52, 939.96)	102.4 (85.6, 120.02)	25.6 (21.4, 30.0)	93.95 (92.04, 95.0)	16.19 (12.24, 19.68)	99.69 (99.63, 99.74)	1.41 (0.69, 2.06)
85	8890	128	FIT test	92.73 (73.13, 129.98)	907.27 (870.02, 926.87)	108.8 (90.95, 127.52)	19.2 (16.05, 22.5)	91.83 (88.03, 93.76)	13.2 (8.99, 17.21)	99.76 (99.72, 99.8)	11.05 (4.45, 18.85)
85	8890	128	Nottingham-Cox	86.7 (68.75, 127.34)	913.3 (872.66, 931.25)	108.8 (90.95, 127.52)	19.2 (16.05, 22.5)	92.44 (88.29, 94.22)	14.12 (9.46, 18.28)	99.76 (99.72, 99.8)	0.83 (0.4, 1.46)
85.94	8890	128	FIT test	95.95 (80.03, 145.85)	904.05 (854.15, 919.97)	110.0 (110.0, 110.0)	18.0 (18.0, 18.0)	91.52 (86.46, 93.14)	12.9 (8.54, 15.47)	99.78 (99.76, 99.78)	10.0 (3.44, 14.95)
85.94	8890	128	Nottingham-Cox	87.63 (79.13, 128.76)	912.37 (871.24, 920.87)	110.0 (110.0, 110.0)	18.0 (18.0, 18.0)	92.36 (88.19, 93.23)	14.12 (9.62, 15.69)	99.78 (99.77, 99.78)	0.78 (0.39, 0.98)
90	8890	128	FIT test	130.75 (87.75, 193.6)	869.25 (806.4, 912.52)	115.2 (96.3, 135.02)	12.8 (10.7, 15.0)	88.05 (81.64, 92.51)	9.91 (6.42, 15.0)	99.83 (99.8, 99.86)	4.45 (1.35, 13.05)
90	8890	128	Nottingham-Cox	127.47 (83.18, 291.42)	872.53 (708.58, 916.82)	115.2 (96.3, 135.02)	12.8 (10.7, 15.0)	88.38 (71.71, 92.87)	10.17 (4.47, 16.2)	99.83 (99.79, 99.86)	0.4 (0.22, 0.98)
95	8890	128	FIT test	195.39 (127.85, 552.19)	804.61 (447.81, 872.15)	121.6 (101.65, 142.52)	6.4 (5.35, 7.5)	81.56 (45.29, 88.41)	7.0 (2.47, 10.98)	99.91 (99.84, 99.93)	1.35 (0.65, 4.45)
95	8890	128	Nottingham-Cox	325.71 (123.16, 393.71)	674.29 (606.29, 876.84)	121.6 (101.65, 142.52)	6.4 (5.35, 7.5)	68.34 (61.46, 88.83)	4.2 (3.21, 11.26)	99.89 (99.87, 99.92)	0.2 (0.18, 0.4)
99	8890	128	FIT test	828.54 (462.18, 910.44)	171.46 (89.56, 537.82)	126.72 (105.93, 148.52)	1.28 (1.07, 1.5)	17.38 (9.06, 54.6)	1.72 (1.4, 3.25)	99.92 (99.84, 99.97)	0.65 (0.65, 0.65)
99	8890	128	Nottingham-Cox	728.76 (336.06, 879.27)	271.24 (120.73, 663.94)	126.72 (105.93, 148.52)	1.28 (1.07, 1.5)	27.51 (12.23, 67.33)	1.96 (1.45, 4.37)	99.95 (99.87, 99.98)	0.09 (0.06, 0.2)
**Post-COVID (2021/05 - 2021/12)**											
80	7472	99	FIT test	96.41 (68.93, 121.76)	903.59 (878.24, 931.07)	79.2 (64.8, 94.4)	19.8 (16.2, 23.6)	91.3 (88.71, 94.08)	10.99 (8.26, 15.48)	99.71 (99.65, 99.76)	11.5 (5.5, 24.51)
80	7472	99	Nottingham-Cox	90.36 (59.12, 142.89)	909.64 (857.11, 940.88)	79.2 (64.8, 94.4)	19.8 (16.2, 23.6)	91.92 (86.59, 94.99)	11.73 (6.93, 18.02)	99.71 (99.65, 99.76)	0.85 (0.38, 2.6)
82.83	7472	99	FIT test	102.11 (87.69, 120.85)	897.89 (879.15, 912.31)	82.0 (82.0, 82.0)	17.0 (17.0, 17.0)	90.76 (88.86, 92.23)	10.75 (9.09, 12.53)	99.75 (99.74, 99.75)	10.0 (7.0, 14.4)
82.83	7472	99	Nottingham-Cox	98.23 (69.86, 136.44)	901.77 (863.56, 930.14)	82.0 (82.0, 82.0)	17.0 (17.0, 17.0)	91.16 (87.29, 94.03)	11.17 (8.04, 15.85)	99.75 (99.74, 99.76)	0.68 (0.38, 1.72)
85	7472	99	FIT test	112.06 (91.02, 139.49)	887.94 (860.51, 908.98)	84.15 (68.85, 100.3)	14.85 (12.15, 17.7)	89.79 (87.01, 91.89)	10.05 (7.43, 13.27)	99.78 (99.73, 99.82)	7.5 (3.5, 13.5)
85	7472	99	Nottingham-Cox	134.92 (82.49, 243.78)	865.08 (756.22, 917.51)	84.15 (68.85, 100.3)	14.85 (12.15, 17.7)	87.47 (76.5, 92.76)	8.35 (4.37, 14.01)	99.77 (99.72, 99.82)	0.38 (0.25, 1.08)
90	7472	99	FIT test	129.19 (104.39, 354.92)	870.81 (645.08, 895.61)	89.1 (72.9, 106.2)	9.9 (8.1, 11.8)	88.12 (65.27, 90.62)	9.23 (3.47, 11.74)	99.85 (99.79, 99.88)	5.5 (0.65, 9.5)
90	7472	99	Nottingham-Cox	229.0 (97.87, 379.6)	771.0 (620.4, 902.13)	89.1 (72.9, 106.2)	9.9 (8.1, 11.8)	78.0 (62.75, 91.27)	5.21 (3.05, 12.13)	99.83 (99.78, 99.87)	0.25 (0.17, 0.68)
95	7472	99	FIT test	424.78 (127.55, 677.46)	575.22 (322.54, 872.45)	94.05 (76.95, 112.1)	4.95 (4.05, 5.9)	58.23 (32.64, 88.32)	2.96 (1.78, 10.06)	99.88 (99.79, 99.93)	0.65 (0.65, 5.5)
95	7472	99	Nottingham-Cox	392.4 (183.0, 632.77)	607.6 (367.23, 817.0)	94.05 (76.95, 112.1)	4.95 (4.05, 5.9)	61.51 (37.18, 82.71)	3.21 (1.79, 6.59)	99.89 (99.81, 99.92)	0.17 (0.11, 0.29)
99	7472	99	FIT test	884.96 (641.82, 935.49)	115.04 (64.51, 358.18)	98.01 (80.19, 116.82)	0.99 (0.81, 1.18)	11.65 (6.53, 36.23)	1.48 (1.21, 2.14)	99.88 (99.79, 99.97)	0.65 (0.65, 1.0)
99	7472	99	Nottingham-Cox	955.57 (382.45, 959.59)	44.43 (40.41, 617.55)	98.01 (80.19, 116.82)	0.99 (0.81, 1.18)	4.49 (4.08, 62.57)	1.37 (1.15, 3.66)	99.7 (99.65, 99.98)	0.03 (0.03, 0.17)
**2022 H1 (2022/01 - 2022/06)**											
80	5972	88	FIT test	76.39 (59.11, 98.13)	923.61 (901.87, 940.89)	70.4 (56.8, 85.6)	17.6 (14.2, 21.4)	93.44 (91.24, 95.18)	15.43 (10.9, 20.89)	99.68 (99.61, 99.74)	34.5 (19.5, 61.0)
80	5972	88	Nottingham-Cox	63.7 (50.63, 91.9)	936.3 (908.1, 949.37)	70.4 (56.8, 85.6)	17.6 (14.2, 21.4)	94.73 (91.88, 96.09)	18.51 (11.94, 24.62)	99.69 (99.62, 99.75)	3.06 (1.5, 4.68)
85	5972	88	FIT test	91.66 (71.02, 199.31)	908.34 (800.69, 928.98)	74.8 (60.35, 90.95)	13.2 (10.65, 16.05)	91.97 (80.98, 94.04)	13.66 (6.38, 18.93)	99.76 (99.7, 99.8)	22.5 (2.5, 37.5)
85	5972	88	Nottingham-Cox	83.02 (58.14, 130.65)	916.98 (869.35, 941.86)	74.8 (60.35, 90.95)	13.2 (10.65, 16.05)	92.85 (87.97, 95.35)	15.09 (9.35, 22.39)	99.76 (99.7, 99.81)	1.75 (0.5, 3.7)
89.77	5972	88	FIT test	121.73 (80.58, 231.51)	878.27 (768.49, 919.42)	79.0 (79.0, 79.0)	9.0 (9.0, 9.0)	88.99 (77.85, 93.16)	10.87 (5.71, 16.42)	99.83 (99.8, 99.84)	11.0 (2.0, 31.8)
89.77	5972	88	Nottingham-Cox	96.28 (75.32, 247.62)	903.72 (752.38, 924.68)	79.0 (79.0, 79.0)	9.0 (9.0, 9.0)	91.57 (76.21, 93.7)	13.74 (5.34, 17.82)	99.83 (99.8, 99.84)	1.28 (0.23, 2.33)
90	5972	88	FIT test	139.25 (78.63, 394.43)	860.75 (605.57, 921.37)	79.2 (63.9, 96.3)	8.8 (7.1, 10.7)	87.21 (61.3, 93.34)	9.52 (3.27, 17.0)	99.83 (99.76, 99.86)	7.4 (1.0, 31.5)
90	5972	88	Nottingham-Cox	103.68 (67.94, 325.09)	896.32 (674.91, 932.06)	79.2 (63.9, 96.3)	8.8 (7.1, 10.7)	90.82 (68.24, 94.41)	12.79 (3.76, 19.31)	99.84 (99.77, 99.87)	0.99 (0.2, 2.9)
95	5972	88	FIT test	435.26 (117.16, 697.22)	564.74 (302.78, 882.84)	83.6 (67.45, 101.65)	4.4 (3.55, 5.35)	57.24 (30.65, 89.47)	3.22 (1.87, 11.55)	99.87 (99.76, 99.92)	1.0 (1.0, 11.5)

95	5972	88	Nottingham-Cox	321.27 (96.68, 502.17)	678.73 (497.83, 903.32)	83.6 (67.45, 101.65)	4.4 (3.55, 5.35)	68.81 (50.47, 91.64)	4.36 (2.62, 14.81)	99.89 (99.85, 99.93)	0.2 (0.14, 1.29)
99	5972	88	FIT test	887.05 (652.4, 939.44)	112.95 (60.56, 347.6)	87.12 (70.29, 105.93)	0.88 (0.71, 1.07)	11.45 (6.13, 35.28)	1.64 (1.34, 2.32)	99.87 (99.76, 99.96)	1.0 (1.0, 1.0)
99	5972	88	Nottingham-Cox	666.3 (357.84, 676.86)	333.7 (323.14, 642.16)	87.12 (70.29, 105.93)	0.88 (0.71, 1.07)	33.85 (32.77, 65.13)	2.19 (1.83, 4.15)	99.96 (99.95, 99.98)	0.1 (0.1, 0.18)
**2022 H2 (2022/07 - 2022/12)**											
80	7490	104	FIT test	97.01 (76.79, 122.91)	902.99 (877.09, 923.21)	83.2 (68.0, 100.0)	20.8 (17.0, 25.0)	91.29 (88.7, 93.3)	11.45 (8.4, 15.0)	99.69 (99.63, 99.75)	25.5 (15.0, 41.5)
80	7490	104	Nottingham-Cox	84.27 (55.4, 109.24)	915.73 (890.76, 944.6)	83.2 (68.0, 100.0)	20.8 (17.0, 25.0)	92.58 (90.09, 95.47)	13.18 (9.94, 21.1)	99.7 (99.64, 99.75)	2.15 (0.99, 4.18)
85	7490	104	FIT test	115.14 (92.33, 151.59)	884.86 (848.41, 907.67)	88.4 (72.25, 106.25)	15.6 (12.75, 18.75)	89.52 (85.84, 91.9)	10.25 (7.06, 13.79)	99.76 (99.72, 99.81)	17.5 (9.4, 28.5)
85	7490	104	Nottingham-Cox	98.85 (66.0, 145.8)	901.15 (854.2, 934.0)	88.4 (72.25, 106.25)	15.6 (12.75, 18.75)	91.17 (86.44, 94.53)	11.94 (7.71, 18.49)	99.77 (99.72, 99.81)	1.44 (0.49, 3.34)
88.46	7490	104	FIT test	133.24 (104.47, 163.97)	866.76 (836.03, 895.53)	92.0 (75.9, 115.0)	12.0 (9.9, 15.0)	87.73 (84.62, 90.57)	9.22 (7.49, 12.78)	99.82 (99.77, 99.85)	12.0 (8.0, 22.5)
88.46	7490	104	Nottingham-Cox	118.96 (88.01, 187.27)	881.04 (812.73, 911.99)	92.0 (83.95, 92.0)	12.0 (10.95, 12.0)	89.18 (82.25, 92.32)	10.33 (6.45, 13.96)	99.82 (99.8, 99.84)	0.84 (0.32, 1.85)
90	7490	104	FIT test	147.68 (109.6, 325.91)	852.32 (674.09, 890.4)	93.6 (76.5, 112.5)	10.4 (8.5, 12.5)	86.29 (68.28, 90.11)	8.46 (3.75, 11.92)	99.84 (99.79, 99.87)	9.4 (1.0, 19.5)
90	7490	104	Nottingham-Cox	142.94 (89.25, 216.46)	857.06 (783.54, 910.75)	93.6 (76.5, 112.5)	10.4 (8.5, 12.5)	86.77 (79.32, 92.24)	8.74 (5.55, 14.15)	99.84 (99.8, 99.87)	0.51 (0.27, 1.84)
95	7490	104	FIT test	367.53 (146.51, 662.95)	632.47 (337.05, 853.49)	98.8 (80.75, 118.75)	5.2 (4.25, 6.25)	64.07 (34.14, 86.48)	3.59 (1.91, 9.55)	99.89 (99.78, 99.93)	1.0 (1.0, 9.4)
95	7490	104	Nottingham-Cox	217.86 (138.05, 428.2)	782.14 (571.8, 861.95)	98.8 (80.75, 118.75)	5.2 (4.25, 6.25)	79.24 (57.95, 87.29)	6.05 (2.85, 10.19)	99.91 (99.87, 99.93)	0.27 (0.16, 0.51)
99	7490	104	FIT test	873.51 (610.61, 932.59)	126.49 (67.41, 389.39)	102.96 (84.15, 123.75)	1.04 (0.85, 1.25)	12.81 (6.83, 39.48)	1.57 (1.29, 2.32)	99.89 (99.78, 99.96)	1.0 (1.0, 1.0)
99	7490	104	Nottingham-Cox	429.37 (327.42, 756.74)	570.63 (243.26, 672.58)	102.96 (84.15, 123.75)	1.04 (0.85, 1.25)	57.85 (24.65, 68.08)	3.2 (1.57, 4.59)	99.98 (99.94, 99.98)	0.16 (0.07, 0.19)
**2023 H1 (2023/01 - 2023/06)**											
80	8320	91	FIT test	97.76 (68.29, 124.53)	902.24 (875.47, 931.71)	72.8 (57.6, 87.22)	18.2 (14.4, 21.8)	91.0 (88.31, 93.94)	8.95 (6.58, 12.47)	99.76 (99.71, 99.81)	35.5 (18.5, 77.5)
80	8320	91	Nottingham-Cox	86.15 (69.42, 103.95)	913.85 (896.05, 930.58)	72.8 (57.6, 87.22)	18.2 (14.4, 21.8)	92.17 (90.4, 93.85)	10.16 (7.73, 13.4)	99.76 (99.71, 99.81)	2.53 (1.81, 3.85)
85	8320	91	FIT test	117.02 (89.98, 135.26)	882.98 (864.74, 910.02)	77.35 (61.2, 92.67)	13.65 (10.8, 16.35)	89.11 (87.26, 91.8)	7.94 (5.93, 11.0)	99.81 (99.78, 99.85)	22.5 (17.5, 44.5)
85	8320	91	Nottingham-Cox	100.28 (78.77, 156.11)	899.72 (843.89, 921.23)	77.35 (61.2, 92.67)	13.65 (10.8, 16.35)	90.8 (85.18, 93.0)	9.27 (5.9, 12.68)	99.82 (99.78, 99.86)	1.94 (0.59, 3.22)
90	8320	91	FIT test	130.98 (102.71, 344.17)	869.02 (655.83, 897.29)	81.9 (64.8, 98.12)	9.1 (7.2, 10.9)	87.75 (66.24, 90.55)	7.52 (2.96, 9.75)	99.87 (99.82, 99.9)	17.5 (1.0, 30.5)
90	8320	91	Nottingham-Cox	111.17 (89.41, 212.74)	888.83 (787.26, 910.59)	81.9 (64.8, 98.12)	9.1 (7.2, 10.9)	89.76 (79.47, 92.0)	8.86 (4.16, 11.48)	99.88 (99.84, 99.9)	1.48 (0.31, 2.43)
92.31	8320	91	FIT test	159.62 (105.11, 274.86)	840.38 (725.14, 894.89)	84.0 (67.5, 96.0)	7.0 (5.62, 8.0)	84.88 (73.23, 90.37)	6.33 (3.28, 9.59)	99.9 (99.88, 99.92)	11.0 (3.0, 29.0)
92.31	8320	91	Nottingham-Cox	170.31 (99.59, 216.3)	829.69 (783.7, 900.41)	84.0 (71.4, 96.0)	7.0 (5.95, 8.0)	83.8 (79.15, 90.95)	5.93 (4.27, 10.14)	99.9 (99.88, 99.92)	0.47 (0.31, 1.95)
95	8320	91	FIT test	389.8 (128.48, 672.09)	610.2 (327.91, 871.52)	86.45 (68.4, 103.57)	4.55 (3.6, 5.45)	61.64 (33.12, 88.06)	2.67 (1.43, 8.21)	99.91 (99.82, 99.94)	1.0 (1.0, 17.5)
95	8320	91	Nottingham-Cox	213.88 (109.42, 294.74)	786.12 (705.26, 890.58)	86.45 (68.4, 103.57)	4.55 (3.6, 5.45)	79.43 (71.24, 89.96)	4.86 (3.35, 9.28)	99.93 (99.91, 99.95)	0.31 (0.22, 1.62)
99	8320	91	FIT test	877.96 (452.0, 934.42)	122.04 (65.58, 548.0)	90.09 (71.28, 107.93)	0.91 (0.72, 1.09)	12.33 (6.62, 55.34)	1.23 (0.97, 2.16)	99.91 (99.82, 99.98)	1.0 (1.0, 1.0)
99	8320	91	Nottingham-Cox	363.95 (212.15, 372.71)	636.05 (627.29, 787.85)	90.09 (71.28, 107.93)	0.91 (0.72, 1.09)	64.3 (63.38, 79.6)	2.98 (2.43, 5.36)	99.98 (99.98, 99.99)	0.18 (0.18, 0.31)
**All data (2017/01 - 2023/08)**											
80	51477	659	FIT test	86.37 (77.26, 95.91)	913.63 (904.09, 922.74)	527.2 (488.0, 564.0)	131.8 (122.0, 141.0)	92.29 (91.32, 93.23)	11.86 (10.32, 13.52)	99.72 (99.7, 99.74)	21.75 (17.05, 27.06)
80	51477	659	Nottingham-Cox	76.29 (68.94, 85.01)	923.71 (914.99, 931.06)	527.2 (488.0, 564.0)	131.8 (122.0, 141.0)	93.31 (92.45, 94.03)	13.42 (11.78, 15.02)	99.72 (99.7, 99.74)	1.83 (1.41, 2.29)
85	51477	659	FIT test	106.81 (93.97, 120.2)	893.19 (879.8, 906.03)	560.15 (518.5, 599.25)	98.85 (91.5, 105.75)	90.28 (88.94, 91.59)	10.19 (8.87, 11.86)	99.79 (99.77, 99.8)	13.05 (10.05, 17.35)
85	51477	659	Nottingham-Cox	96.14 (82.78, 111.5)	903.86 (888.5, 917.22)	560.15 (518.5, 599.25)	98.85 (91.5, 105.75)	91.36 (89.82, 92.71)	11.32 (9.63, 13.4)	99.79 (99.77, 99.8)	0.99 (0.68, 1.48)
87.56	51477	659	FIT test	120.33 (120.42, 127.09)	879.67 (872.91, 879.58)	577.0 (577.0, 577.0)	82.0 (82.0, 82.0)	88.95 (88.26, 88.94)	9.32 (8.82, 9.31)	99.82 (99.82, 99.82)	10.0 (9.04, 10.36)
87.56	51477	659	Nottingham-Cox	111.29 (100.06, 126.65)	888.71 (873.35, 899.94)	577.0 (577.0, 577.0)	82.0 (82.0, 82.0)	89.86 (88.31, 91.0)	10.07 (8.86, 11.2)	99.82 (99.82, 99.82)	0.68 (0.51, 0.89)
90	51477	659	FIT test	150.91 (119.87, 188.59)	849.09 (811.41, 880.13)	593.1 (549.0, 634.5)	65.9 (61.0, 70.5)	85.88 (82.09, 89.03)	7.63 (6.1, 9.79)	99.85 (99.84, 99.86)	5.05 (3.05, 10.05)
90	51477	659	Nottingham-Cox	143.13 (111.38, 190.89)	856.87 (809.11, 888.62)	593.1 (549.0, 634.5)	65.9 (61.0, 70.5)	86.67 (81.84, 89.9)	8.05 (5.97, 10.48)	99.85 (99.84, 99.86)	0.4 (0.29, 0.68)
95	51477	659	FIT test	419.03 (240.14, 551.49)	580.97 (448.51, 759.86)	626.05 (579.5, 669.75)	32.95 (30.5, 35.25)	58.79 (45.39, 76.92)	2.9 (2.17, 5.14)	99.89 (99.86, 99.92)	0.65 (0.65, 1.35)
95	51477	659	Nottingham-Cox	323.19 (219.44, 373.17)	676.81 (626.83, 780.56)	626.05 (579.5, 669.75)	32.95 (30.5, 35.25)	68.49 (63.45, 78.97)	3.76 (3.22, 5.5)	99.91 (99.9, 99.92)	0.2 (0.18, 0.26)
99	51477	659	FIT test	883.81 (840.81, 910.3)	116.19 (89.7, 159.19)	652.41 (603.9, 697.95)	6.59 (6.1, 7.05)	11.76 (9.08, 16.11)	1.43 (1.32, 1.56)	99.89 (99.86, 99.92)	0.65 (0.65, 0.65)
99	51477	659	Nottingham-Cox	685.19 (563.2, 792.37)	314.81 (207.63, 436.8)	652.41 (605.04, 697.95)	6.59 (6.11, 7.05)	31.88 (21.02, 44.24)	1.85 (1.55, 2.28)	99.96 (99.94, 99.97)	0.09 (0.07, 0.12)
