# Supplementary material for: External validation of the COLOFIT colorectal cancer risk prediction model in the Oxford-FIT dataset: the importance of population characteristics and clinically relevant evaluation metrics
Source: BMC Med. 2025 Aug 27;23:503. doi: 10.1186/s12916-025-04339-w (PMC12392603; doi:10.1186/s12916-025-04339-w)
Supplement: Supplementary file 15 — Additional File 15: Reduction in referrals before and after buffer device adoption: Figure S15. Fig S15 – Percent reduction in the number of positive tests and percent cancers missed compared to FIT ≥ 10 µg/g before and after buffer device adoption [file 12916_2025_4339_MOESM15_ESM.pdf]

S15. REDUCTION IN REFERRALS BEFORE AND AFTER BUFFER DEVICE ADOPTION

Here we analyse reduction in referrals on three subsets of Oxford FIT data: data collected before the buffer device was adopted ("pre-buffer data"), and data collected after the buffer device was adopted ("buffer data from time", "buffer data from comment"). The "pre-buffer" data represents FIT tests done before the buffer devices were adopted (mostly stool pots). The "buffer data from time" refers to the period when the buffer device was assessed to have been adopted by the clinical biochemistry laboratory. The "buffer data from comment" refers to FIT tests known to have been done with the buffer device based on comments given in the FIT test result (comments were available only from April 2022 and not earlier).

Potential reduction in referrals relative to FIT ≥ 10 µg/g was -8.74% reduction (95% CI [-10.48, -6.97]) in pre-buffer data, -4.93% (95% CI [-6.11, -3.7]) in "buffer data from time", and -6.73% reduction (95% CI [-8.21, -5.42]) in "buffer data from comment" (Figure S15). There were no strong differences observed between these data subsets. However, please note that comparisons of pre- and post-buffer datasets are confounded by changes in patient population (see Section S7 above), so any differences between these datasets cannot be attributed to the faecal sampling device.

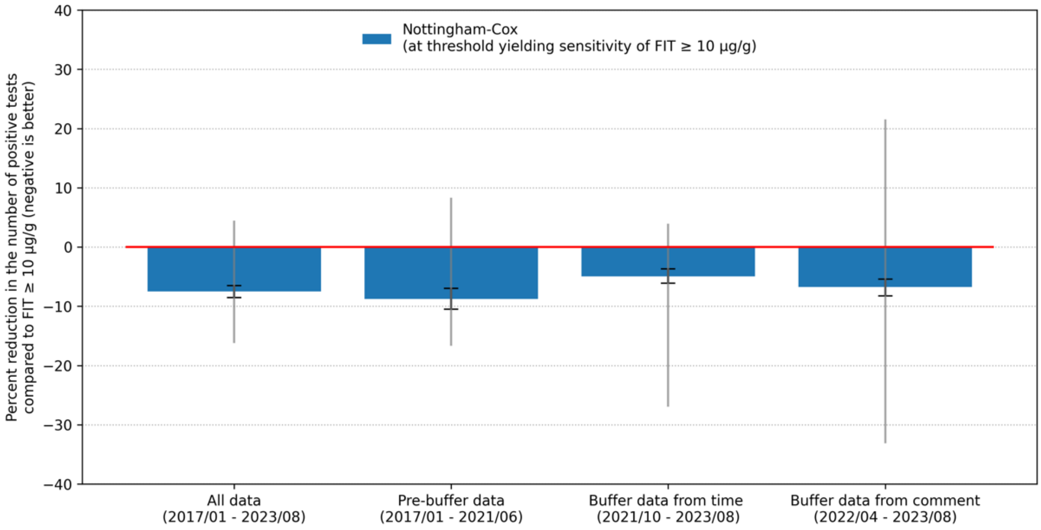

Figure S15. Percent reduction in the number of positive tests (referrals) and percent cancers missed compared to FIT ≥ 10 µg/g before and after buffer device adoption. The model was evaluated at a threshold that captured the same number of cancers as FIT ≥10 µg/g in all data, and before and after buffer device adoption. The "pre-buffer" data represents FIT tests done before the buffer devices were adopted (mostly stool pots). The "buffer data from time" refers to the period when the buffer device was assessed to have been adopted by the clinical biochemistry laboratory. The "buffer data from comment" refers to FIT tests known to have been done with the buffer device based on comments given in the FIT test result (comments were available only from April 2022). Values below zero indicate reduction in the number of tests: for example, -20 means 20% less patients test positive when the model is used. Cancers were detected using a 180-day follow-up. Wider grey confidence intervals are computed by re-evaluating the threshold in each bootstrap sample, and narrower black intervals use the threshold estimated on original data (this is closer to how the model is used in practice, as in practice a single threshold would be used).
