# Supplementary material for: External validation of the COLOFIT colorectal cancer risk prediction model in the Oxford-FIT dataset: the importance of population characteristics and clinically relevant evaluation metrics
Source: BMC Med. 2025 Aug 27;23:503. doi: 10.1186/s12916-025-04339-w (PMC12392603; doi:10.1186/s12916-025-04339-w)
Supplement: Supplementary file 16 — Additional File 16: Contributions of age, sex and bloods to reduction in referrals: Figure S16. Fig S16 – Percent reduction in the number of positive tests and percent cancers missed compared to FIT ≥ 10 µg/g over time in OUH-FIT data, for the full and reduced COLOFIT-Cox models [file 12916_2025_4339_MOESM16_ESM.pdf]

S16. CONTRIBUTIONS OF AGE, SEX AND BLOODS TO REDUCTION IN REFERRALS

We also explored how omitting some predictors influences model performance. We computed reduction in referrals for the full model and for reduced linear predictors that contained (a) FIT and age; (b) FIT, age and sex; (c) FIT and MCV; or (d) FIT, MCV and PLT. This revealed that the inclusion of age and sex was detrimental to performance during the first half of 2023: a linear predictor including FIT and bloods was associated with a -8.65% reduction in referrals; FIT, age and sex with a +33.02% increase; and the full model with a 2.16% increase (Figure S16). In contrast, the inclusion of bloods did not benefit the model during the eight post-COVID months: FIT and bloods were associated with a 0.66% increase in referrals; FIT, age and sex with a -4.98% reduction; and the full model with a -3.8% reduction (Figure S16).

Note: why is it valid to analyse the contribution of individual variables to the reduction in referrals by excluding them from the linear predictor? The COLOFIT Cox model predicts the probability that cancer will occur within a 1-year period by computing “1 – baseline_survival^exp(linear_predictor)”. This is a monotonic function of the linear predictor, so the ordering of patients based on their predicted probabilities of cancer is the same as the ordering based on their linear predictors, and the discrimination metrics computed from the predicted probabilities and linear predictors are also the same.

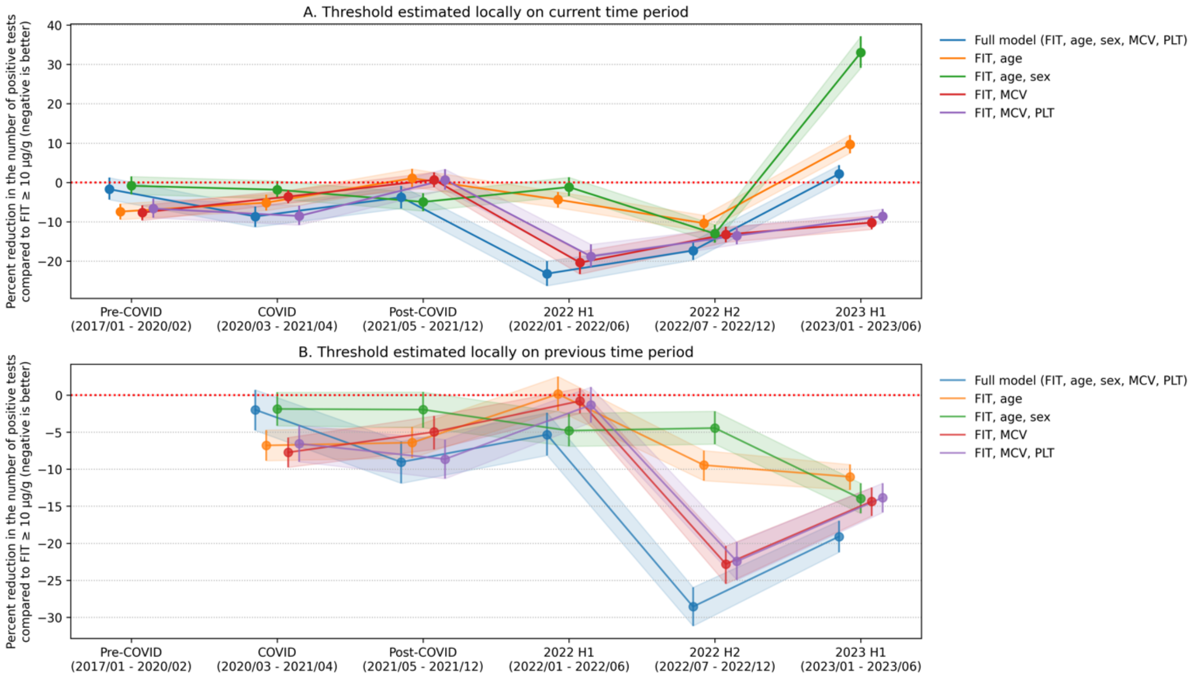

Figure S16. Percent reduction in the number of positive tests (referrals) and percent cancers missed compared to FIT ≥10 µg/g over time in OUH-FIT data, for the full and reduced COLOFIT-Cox models. The models were evaluated at a threshold that captured the same number of cancers as FIT ≥10 µg/g in the current Oxford data subset (upper panel) or in the previous Oxford data subset (lower panel). Reduction in referrals was computed for the full model (blue), and for reduced linear predictors that contained only some predictor variables: FIT and age (orange); FIT, age and sex (green); FIT and MCV (red); or FIT, MCV and PLT (purple). When percent reduction is negative, it means that the number of patients who would have tested positive according to the model was smaller than the number of patients who tested positive for FIT, and so there was potentially a reduction in referrals. Shaded areas show 95% percentile bootstrap confidence intervals. The points are slightly jittered on the x-axis to avoid overlap.
