# Supplementary material for: External validation of the COLOFIT colorectal cancer risk prediction model in the Oxford-FIT dataset: the importance of population characteristics and clinically relevant evaluation metrics
Source: BMC Med. 2025 Aug 27;23:503. doi: 10.1186/s12916-025-04339-w (PMC12392603; doi:10.1186/s12916-025-04339-w)
Supplement: Supplementary file 17 — Additional File 17: Evaluation of FIT-based risk prediction models in the literature: Table S17. Tab S17 – FIT-based colorectal cancer risk prediction models and their evaluation [file 12916_2025_4339_MOESM17_ESM.pdf]

S17. EVALUATION OF FIT-BASED RISK PREDICTION MODELS IN THE LITERATURE

FIT-based risk prediction models. Several colorectal cancer risk prediction models have been developed on the data of symptomatic patients, using the FIT test and other routinely collected variables as predictors (Table S17): seven studies describe the derivation of models [20, 26, 37, 40, 42–44] and three studies further validate models [38, 39, 41]. The studies listed in Table S17 have been included in a recent review by Hampton et al [19] with the exception of Lucocq et al [43] and Digby et al [44] probably because these are newer. Compared to Hampton et al [19], Table S17 does not include studies that incorporate less common laboratory tests such as the faecal calprotectin, that use gFOBT rather than FIT, or that only report the performance of a combination of predictors without a model.

Evaluation of FIT-based models. A variety of metrics have been used to evaluate FIT-based models (Table S17). Discrimination metrics include (1) *c*-statistic and ROC-curve; (2) basic diagnostic metrics such as sensitivity, specificity, positive predictive value, negative predictive value, percent positives, and other related metrics at various risk thresholds. Calibration metrics include the Hosmer-Lemeshow (H-L) test and curve based on deciles of predicted risk. Two studies also reported decision curves (net benefit statistics). Reduction in the number of patients referred compared to the FIT test alone or FIT and other criteria have also been reported.

Key performance metrics. The relevance of these metrics depends on the intended use of the model. The purpose of FIT-based models is to arguably reduce the number of patients that would have been referred to colonoscopy based on FIT alone. The model should also capture the same (or a very similar) number of cancers as FIT at the NICE-recommended threshold of ≥ 10 µg/g to be comparable to the current clinical practice. A key metric of model performance is then the reduction in the number of patients that test positive at a risk score threshold that captures the same number of cancers as FIT ≥ 10 µg/g, compared to FIT alone. Among the studies reviewed, this metric has only been reported in the COLOFIT study [37]. Six other studies [37–42] that developed models on referred patients reported the proportion of patients testing positive at high levels of sensitivity, but this was reported relative to referring patients based on FIT and other criteria, and does not show the potential reduction in referrals compared to FIT alone. Positive predictive value, when reported for the model and FIT at the same sensitivity as in Withrow et al [26], is closely related to reduction in the number of positive tests: the ratio of the number of positive tests of the model and FIT (pos_model/pos_fit) is equal to the ratio of the PPVs of FIT and model (ppv_fit/ppv_model) when the true positive count (sensitivity) is the same. The second important class of metrics is calibration, as practitioners may want to interpret risk scores as probabilities of cancer.

Other metrics. The other metrics are informative but less useful. The *c*-statistic summarises performance over all risk thresholds, whereas in real world the model would likely be used at a threshold that captures most cancers like FIT ≥ 10 µg/g. The ROC-curve displays sensitivity and false positive rate for each risk threshold, but when the prevalence of cancer is small, the false positive rates of different models can be very similar even though some models can have a much higher PPV at some risk thresholds; in that case the precision-recall curve can be more informative [31]. Basic diagnostic metrics (like PPV) are familiar to researchers, but they should be reported for the model and FIT at comparable risk thresholds, as otherwise it is not clear whether differences observed in the metrics would change if the model and/or FIT would simply be used at a different threshold.

Limitations of decision curves. Crooks et al [20] and Lucocq et al [43] have also reported decision curves. The key quantity in decision curve analysis (DCA) [34] is 'net benefit' which is proportional to the number of true positives minus the odds of cancers times the number of false positives. If the model is developed on all patients who did the FIT test and a positive risk score represents referral to colonoscopy, then a false positive is a colonoscopy where no cancer was detected. DCA assumes that the cost of a false positive is higher when the person has a high predicted risk of cancer according to a model, but this assumption may not hold in the real world. On one hand, people with high predicted risk may be more fragile, so a colonoscopy can be more harmful. On the other hand, if someone's cancer risk coincides with the risk of other conditions that can be discovered via additional testing, a colonoscopy where no cancer is detected can still be beneficial. It is not

possible to accurately assess the harms and benefits of additional testing only based on risk scores predicted by a model: additional data and clinical reasoning are needed. However, this is not incorporated into decision curve analysis, which is a major limitation. The same holds for ‘net intervention avoided’, which is sometimes computed in DCA as the true negative rate minus the odds of ‘no cancer’ times false negative rate. Again, only the modelled risk scores are used to assign weight to false negatives. The motivation for DCA is well-founded—quantifying the benefits and harms for every individual—but its implementation may be oversimplified, and the results are not as clear as reporting the total reduction in colonoscopy referrals that the model would produce relative to the FIT test.

Conclusion. FIT-based models have been evaluated using a variety of statistics, but only the COLOFIT study [20] has reported the potential reduction in referrals when using the model compared to the FIT test alone.

Table S17. FIT-based colorectal cancer risk prediction models and their evaluation

Study	Year	Study type	Model name	Predictor variables	Model type	Population	Sample size	Evaluation metrics	Region	Percent FIT ≥ 10 µg/g
Cubiella et al [37]	2017	Derivation and external validation	FAST	FIT, age, sex	logistic	Symptomatic patients referred to colonoscopy from primary and secondary care	1,572 (254 cancers) in derivation, 3976 (about 207 cancers) in validation.	• *c*-statistic and ROC curve for the FAST score.<br>• Sensitivity, specificity, PPV, NPV, PLR, NLR, and OR for the FAST score at thresholds that yielded 90% and 99% sensitivities in derivation data.<br>• PPV, NPV, NNS and percent patients in low/high/intermediate risk groups according to the FAST score.	Spain (derivation), Scotland (validation)	44.4% in derivation[41], not reported for validation data.
Digby et al [38]	2019	External validation	FAST	FIT, age, sex	logistic	Symptomatic patients with GP-requested FITs who completed colonoscopy (group A) or were not immediately referred to colonoscopy (group B).	1,447 (95 cancers) in group A, 2,521 (4 cancers) in group B.	• Reduction in colonoscopy referrals, and number of missed cancers, if FAST score would be used at a threshold that yielded 99% sensitivity in derivation data, compared to patients already referred.<br>• Sensitivity, specificity, PPV, NPV for FAST score at a threshold that yielded 99% sensitivity in derivation dataset for patients referred to colonoscopy.	Scotland (validation)	53.9% in group A, 4.8% in group B.
Cama et al [39]	2022	External validation	FAST	FIT, age, sex	logistic	Symptomatic patients with GP-requested FITs who completed colonoscopy (group A) or were not immediately referred to colonoscopy (group B).	924 (56 cancers) in group A, 3190 (0 cancers) in group B.	• Reduction in colonoscopy referrals, and number of missed cancers, if FAST score would be used at a threshold that yielded 99% sensitivity in derivation data, compared to patients already referred.<br>• Sensitivity, specificity, PPV, NPV for FAST score at a threshold that yielded 99% sensitivity in derivation dataset for patients referred to colonoscopy.	England (validation)	67.9% in group A, 4.7% in group B.
Cubiella et al [40]	2016	Derivation and external validation	COLON-PREDICT	FIT, age, sex, haemoglobin, aspirin use, history of colonoscopy, symptoms (rectal mass, rectal bleeding, change in bowel habit), benign anorectal lesion	logistic	Symptomatic patients referred to colonoscopy from primary and secondary care	1,572 (254 cancers) in derivation, 1,481 (136 cancers) in validation.	• *c*-statistic and ROC curve for the model and the NICE CG27 symptom-based referral criteria.<br>• Sensitivity, specificity, PPV, NPV, percent positives, PLR, NLR, and OR – for the model at thresholds that yielded 90% and 99% sensitivities in derivation data, and for the NICE CG27 symptom-based referral criteria.<br>• Sensitivity, specificity, PPV, NPV, and percent positives for the model at thresholds that yielded 50% sensitivity and 90% specificity.<br>• PPV, NPV, NNS, percent patients in low/high/intermediate risk groups.<br>• Calibration curve from Hosmer-Lemeshow test.	Spain (derivation), Spain (validation)	44.4% in[41], not reported for validation data.
Herrero et al [41]	2018	Post-hoc analysis	COLON-PREDICT	Same as in Cubiella et al [40]	logistic	Symptomatic patients referred to colonoscopy from primary and secondary care	1,572 (254 cancers) in derivation data.	• *c*-statistic and ROC curve for the COLONPREDICT model, for FAST score, for NICE NG12 and CG27 symptom-based referral criteria, and for the FIT test.<br>• Sensitivity, specificity, PPV, NPV, PLR, NLR, OR, and percent patients testing positive – for the model and FAST score at thresholds that yielded 90% and 99% sensitivity; for NICE NG12 and CG27 symptom-based referral criteria; and for the FIT test at 10 and 20 µg/g thresholds.	Spain (derivation)	44.4%
Fernández-Bañares et al [42]	2019	Derivation and external validation	COLONO-FIT	Max FIT value over 3 samples, num samples with FIT > 4 over 3 samples, age, smoking status, history of colonoscopy	logistic	Patients with high-risk symptoms referred to fast-track colonoscopy and symptomatic patients with a negative FIT.	867 (67 cancers) in derivation, 628 (49 cancers) in validation.	• *c*-statistic and ROC curve for the COLONOFIT and FAST scores.<br>• Hosmer-Lemeshow test for calibration.<br>• Sensitivity, specificity, PPV, NPV, PLR, NLR, OR, number and percent of positive tests (percent of colonoscopies to prioritise), and number of missed cancers, at 96% sensitivity for the COLONOFIT score.<br>• Proportion of colonoscopies that can be prioritised to detect 98% of cancers.	Spain (derivation and validation)	Not reported
Lucocq et al [43]	2024	Derivation*	-	FIT, age, sex, iron deficiency anaemia, symptoms (abdominal pain, altered bowel habit, rectal	logistic and machine learning models	Symptomatic patients referred to colonoscopy from primary care.	3,776 (217 cancers)	• *c*-statistic and accuracy for multivariable and FIT-only models.<br>• ROC curves for multivariable models.<br>• Sensitivity, specificity, PPV, and NPV for multivariable and FIT-only models at thresholds that maximize the sum of sensitivity and specificity.<br>• Net benefit curves for multivariable prediction models.<br>• Net benefit and net reduction curves for random forest model, and for testing strategies based on random forest risk scores: colonoscopy for all, for high-risk only, for intermediate and high-risk.	Scotland (derivation)	57.7%

				bleeding, weight loss, other)						
Withrow et al [26]	2022	Derivation	-	Model A: FIT, age, sex, ferritin, platelets, c-reactive protein. Models B and C: FIT, sex, low mean cell volume.	logistic	Patients with GP-requested FITs	16,604 (139 cancers)	• *c*-statistic for FAST score<br>• Specificity, PPV, NPV, NNS, cancer miss rate per 10.000 negative tests – for the models at the same level of sensitivity as FIT ≥ 10 µg/g	England (derivation)	9.2%
Crooks et al [20]	2022	Derivation and internal validation	COLOFIT	FIT, age, sex, mean cell volume, platelet count.	Cox ph	Patients with GP-requested FITs	34,231 (516 cancers) in derivation, 16,735 (206 cancers) in validation.	• *c*-statistic for the COLOFIT model<br>• Sensitivity, specificity, PPV, NPV; number of positive tests (colonoscopies), true positives (detected cancers), false negatives (missed cancers), and false positives (normal colonoscopies) – for the COLOFIT and local FIT-age-sex models at the 0.64%, 1%, 2% and 3% thresholds of predicted risk; and for FIT test at thresholds 10 and 40. (0.64% was the predicted risk of cancer for FIT test at threshold 10).<br>• Net benefit curves for the COLOFIT model, for a FIT-only model, and for FIT test at thresholds 10 and 40.<br>• Calibration curves based on deciles of predicted risk for the COLOFIT model.	England (derivation and validation)	Not reported
Digby et al [44]	2024	Derivation and internal validation	-	FIT, age, sex, iron deficiency anaemia, systemic inflammation index (from platelet, neutrophil, and lymphocyte counts).	logistic	Patients with GP-requested FITs	9,374 (155 cancers) in derivation, 9,431 (142 cancers) in validation.	• Number of positive tests, number of detected cancers, sensitivity, specificity, PPV, NPV, NNS – for the model at 90% and 99% sensitivity thresholds, for the FIT test at threshold 10, for the model at a threshold that yielded the same number of positive tests as FIT test at threshold 10, and for a combined testing strategy that applied a model to patients with FIT between 10 and 20 and otherwise referred patients with FIT > 20.	Scotland (derivation and validation)	25% in derivation, 24.9% in validation.

Note. *Lucocq et al used cross-validation for building prediction models which can be considered a form of internal-external validation, but it is not clear whether metrics of model performance were reported on held-out cross-validation folds, or on all data, and that is why it is marked as ‘derivation’ only in this table. Abbreviations: Cox ph – Cox proportional hazards; FAST – “FIT, age, and sex test”; OV – overall value; OR – odds ratio; PLR – positive likelihood ratio; PPV – positive predictive value; NLR – negative likelihood ratio; NNS – number needed to scope; NPV – negative predictive value.
