# Supplementary material for: External validation of the COLOFIT colorectal cancer risk prediction model in the Oxford-FIT dataset: the importance of population characteristics and clinically relevant evaluation metrics
Source: BMC Med. 2025 Aug 27;23:503. doi: 10.1186/s12916-025-04339-w (PMC12392603; doi:10.1186/s12916-025-04339-w)
Supplement: Supplementary file 18 — Additional File 18: Reduction in referrals produced by FIT-based risk prediction models in the literature [file 12916_2025_4339_MOESM18_ESM.pdf]

S18. REDUCTION IN REFERRALS PRODUCED BY FIT-BASED RISK PREDICTION MODELS IN THE LITERATURE

Compared to existing FIT-based CRC risk prediction models, COLOFIT was derived on the largest dataset at the time (34,231 patients) and is one of three studies conducted on a sample of all primary care FIT testing patients (a pre-referral population) (Supplementary Table S17). Withrow et al [26] derived models that included FIT, demographics and bloods on an older cut of OUH-FIT data (before January 2021), and did not observe improved PPV for their models at the same sensitivity as FIT ≥ 10 μg/g, consistent with less than 2% reduction in referrals seen for COLOFIT in the pre-COVID period in the current Oxford data. Digby et al [44] derived models using FIT, age, sex, iron deficiency anaemia and systemic inflammation index on Scottish data and found that the models would have detected 3 additional cancers compared to FIT for the same number of colonoscopies, and concluded from this and other analyses that the models provided no significant benefit.

The other FIT-based models have been developed primarily on the data of patients referred to colonoscopy. The FAST (FIT, age and sex test) score [37] would have led to 9% [39] or 21% [38] reduction in referrals with one missed cancer, when applied to UK primary care patients referred based on FIT and other criteria, but it is not clear whether FIT alone would have led to a similar reduction when used at a threshold with the same sensitivity as FAST. The COLONPREDICT [40] and COLONOFIT [42] scores were developed on patients referred from both primary and secondary care and may not represent a relevant target population. Lucocq et al [43] developed models on referred primary care patients using FIT, demographics, iron deficiency anaemia and symptoms, and claimed that at 1-3% risk score thresholds 30-33% colonoscopies would be saved. However, they did not report reduction in colonoscopies for a FIT only model for comparison; and may have computed it using decision curve analysis, which uses a weighted sum of true and false negatives not the number of colonoscopies not done.

Overall the literature does not consistently demonstrate that models can reduce referrals while capturing as many cancers as FIT.
